# Health-Related Physical Fitness Evaluation in HIV-Diagnosed Children and Adolescents: A Scoping Review

**DOI:** 10.3390/ijerph21050541

**Published:** 2024-04-25

**Authors:** João Antônio Chula de Castro, Tiago Rodrigues de Lima, Diego Augusto Santos Silva

**Affiliations:** 1 Graduate Program of Physical Education, Sports Center, Federal University of Santa Catarina, Florianopolis 88040-900, SC, Brazil; joaoantoniochula@gmail.com (J.A.C.d.C.); tiagopersonaltrainer@gmail.com (T.R.d.L.); 2 Graduate Program in Human Movement Sciences, University of the State of Santa Catarina, Florianopolis 88080-350, SC, Brazil

**Keywords:** acquired immunodeficiency syndrome, chronic disease, physical activity assessment

## Abstract

Background: Health-related physical fitness has been widely used to investigate the adverse effects of HIV infection/ART in children and adolescents. However, methods/protocols and cut-points applied for investigating health-related physical fitness are not clear. The aim of this scoping review was to map the literature to identify gaps in knowledge regarding the methods/protocols and cut-points. Methods: A scoping review, following the Joana Briggs Institute (JBI) guidelines, was conducted through ten major databases. Search followed the PCC strategy to construct block of terms related to population (children and adolescents), concept (health-related physical fitness components) and context (HIV infection). Results: The search resulted in 7545 studies. After duplicate removal, titles and abstracts reading and full text assessment, 246 studies were included in the scoping review. Body composition was the most investigated component (*n* = 244), followed by muscular strength/endurance (*n* = 23), cardiorespiratory fitness (*n* = 15) and flexibility (*n* = 4). The World Health Organization growth curves, and nationals’ surveys were the most reference values applied to classify body composition (*n* = 149), followed by internal cut-points (*n* = 30) and cut-points developed through small populations (*n* = 16). Cardiorespiratory fitness was classified through cut-points from three different assessment batteries, as well as cut-points developed through studies with small populations, muscular strength/endurance and flexibility were classified through the same cut-points from five different assessment batteries. Conclusions: The research on muscular strength/endurance, cardiorespiratory fitness and flexibility has been scarcely explored. The lack of studies that investigated method usability as well as reference values was evidenced.

## 1. Introduction

In the 1980s, period when the first cases were recorded, the human immunodeficiency virus (HIV) infection with was highly lethal [1,2]. However, in the 1990s, with the introduction of combined antiretroviral therapy (ART), the characterization of the HIV infection was changed from highly lethal to a controllable chronic disease [1,2]. Thus, substantially increasing the life expectancy and quality of life of HIV-infected children and adolescents [1,2,3]. ART’s main objectives are to suppress HIV replication, rebuild immune function and reduce drug resistance, as well as toxicity, allowing for adequate growth and development [1]. Despite being the main way in the treatment of HIV infection, continuous use of ART can lead to adverse effects such as complications of the cardiovascular and nervous systems, dyslipidemia, insulin resistance and changes in body composition, in addition to mitochondrial and renal toxicity [3,4,5]. Furthermore, in 2021, around 160,000 new cases of HIV infection in children and adolescents and 97,500 deaths of HIV-diagnosed children and adolescents under the age of 15 were recorded [6], and this high mortality rate over the number of new cases may be related to the adverse effects of prolonged use of ART [6]. These facts highlight the relevance of continued investigation of the HIV infection mechanism and ART’s possible adverse effects.

The investigation of health-related physical fitness in children and adolescents has been widely used to identify and understand those possible adverse effects of HIV infection/ART use such as changes in body composition (alterations in body fat distribution, reduction in bone mineral content and fat-free mass) [7,8,9,10,11,12,13,14,15,16,17,18,19,20,21]; reduced cardiorespiratory fitness [22,23,24,25,26]; reduced muscular strength/endurance [26,27,28,29,30]; and reduced flexibility [24,26,31]. However, studies that investigated HIV-diagnosed children and adolescents ended up developing their own methods/protocols for evaluating the health-related physical fitness [32,33,34,35] and also used empirical cut-points for physical fitness tests to classify the participants [19,36,37,38,39,40,41,42]. This lack of standardization limits the comparison between studies and the direction of future studies.

Furthermore, the investigation of possible effects of HIV infection/ART use in relation to the different health-related physical fitness components has been carried out in isolation, that is, with studies focusing mainly on one of the components such as body composition without observing other components such as cardiorespiratory fitness [26,30,43,44,45,46,47]. This approach makes sense when the aim of the study is to investigate possible effects of HIV infection/ART use in a specific component. However, previous studies have showed that: (I) muscular strength/endurance is directly associated with the number of muscle fibers, where the greater the number of muscle fibers, the greater the muscular strength/endurance values [48,49]; (II) cardiorespiratory fitness is directly associated with the amount of type I (oxidative) muscle fibers, where the greater the number of type I muscle fibers, the greater the cardiorespiratory fitness [48,49]. Thus, focusing mainly on only one specific component limits the understanding of whether the changes observed in the investigated component were explained by changes in another component, or furthermore, whether these changes result in changes in other components as a ripple effect [28,33,50,51,52].

In addition to the relationship between the components, it is known that improvements in health-related physical fitness components (body composition, cardiorespiratory fitness, muscular strength/endurance and flexibility) can be the result of improvements in physical activity [48,49,53]. Thus, physical activity can moderate the relationship between HIV infection/ART use and the health-related physical fitness components, as well the differences between HIV-diagnosed children and adolescents and their peers without HIV infection diagnosis [48,49,53]. Consequently, the results of studies that investigated health-related physical fitness to understand the relationship with HIV infection/ART use, or to compare HIV-diagnosed children and adolescents with their peers without HIV infection diagnosis, may be misinterpreted due to do not considering physical activity.

Thus, the aim of this scoping review was to map the available literature that investigated the health-related physical fitness in HIV-diagnosed children and adolescents, and through that to identify gaps in knowledge regarding the methods/protocols and cut-points applied. In addition to that, studies that simultaneously researched the health-related physical fitness components and physical activity were identified.

## 2. Materials and Methods

### 2.1. Preliminary Search

The preliminary search was conducted in January 2024, through the search tools of the Medical Literature Analysis and Retrieval System Online (MEDLINE) (via PubMed), Cochrane Library, Joana Briggs Institute (JBI) Evidence Synthesis and Open Science Framework (OSF) to identify scoping reviews or systematic reviews that mapped the health-related physical fitness of HIV-diagnosed children and adolescents. Two systematic reviews with meta-analysis were found, which aimed to investigate muscle function and aerobic capacity in HIV-diagnosed patients [54], and to investigate the aerobic capacity, muscle strength and body composition of HIV-diagnosed adolescents [55]. However, those reviews included only studies that compared results of HIV-diagnosed participants with comparison groups of participants without HIV diagnoses, thus excluding studies that investigated only HIV-diagnosed children and adolescents.

### 2.2. Review Questions

This review aimed to map, compile and disseminate the knowledge regarding the health-related physical fitness of HIV-diagnosed children and adolescents, based on the following questions: which health-related physical fitness components have been investigated in HIV-diagnosed children and adolescents? Which methods/protocols and cut-points were applied to investigate the health-related physical fitness of HIV-diagnosed children and adolescents?

Considering the assumption that the physical activity level is directly related to the health-related physical fitness, in which higher level of physical activity is related to improvements in the health-related physical fitness [48,49,53]. Studies that researched health-related physical fitness and physical activity were investigated to identify the approach that has been given to physical activity and whether the physical activity level has influenced the results found regarding the health-related physical fitness.

### 2.3. Protocol and Checklist

This scoping review (design, conduction and report) was developed following the JBI guidelines [56] and the Preferred Reporting Items for Systematic Reviews and Meta-Analyses Extension for Scoping Reviews (PRISMA-ScR) checklist and explanation [57]. The final protocol of this scoping review was previously registered in the Open Science Framework (OSF) (https://osf.io/gqje7/, accessed on 14 April 2024).

### 2.4. Inclusion Criteria

#### 2.4.1. Participants

Studies that included children and adolescents aged 2 to 19 years and/or with mean age of up to 19 years, diagnosed with HIV infection (with or without comparison groups).

#### 2.4.2. Concept

Studies that investigated the health-related physical fitness components (body composition, cardiorespiratory fitness, muscle strength/endurance and flexibility) through laboratory and/or field tests were included in the review.

#### 2.4.3. Context

There was no context restriction regarding sex, geographic location, ethnicity, viral load and ART use, or physical activity level. However, studies that did not investigate participants with HIV infection diagnosis or did not present the method/protocol to assess the health-related physical fitness component investigated were excluded. Moreover, there was no restriction regarding the time of study publication.

### 2.5. Types of Evidence Sources

Original cross-sectional studies, longitudinal studies, cohort studies, case–control designs and controlled trials studies that were published in peer-review journals, with primary data and explicit description of the methods were included. Dissertations, theses, book chapters, conference abstracts and presentations, point of view or opinion articles, methodological and review articles were excluded.

### 2.6. Text Access

Studies that presented unavailability of access to the full text, even after attempts to contact authors and journals, were excluded.

### 2.7. Search Strategy

Descriptors and their respective entry terms were searched through the Medical Subject Headings (MeSH) and the Health Sciences Descriptors (DECS). The descriptors definition was made in English, Portuguese and Spanish. Boolean operators (“AND” and “OR”) were used to construct blocks of terms and to combine them. The PCC (population, concept, and context) strategy was applied to block of terms construction in which terms related to the population (children and adolescents), concept (health-related physical fitness components) and context (HIV infection) were combined. The option to apply only terms related to the health-related physical fitness components (as concept) was made to avoid studies that only investigated the physical activity level.

The ten following databases were used, independently by two researchers, to identify the articles to the scoping review: Cochrane Library, MEDLINE (via PubMed), Excerpta Medica dataBASE EMBASE (via Ovid), Web of Science, SPORTDiscus (via EBSCOhost), Latin American and Caribbean Health Sciences Literature (LILACS) (via BVS), Scopus, Scientific Electronic Library Online (SciELO) and Science Direct and Cumulative Index to Nursing and Allied Health Literature (CINAHL) (via EBSCOhost). Additional information regarding the search strategy can be found in the Supplementary Materials.

### 2.8. Evidence Screening and Selection

The search results from each database were imported into the EndNote^TM^ 21 reference manager, version 21.2 (Clarivate^TM^, Philadelphia, PA, USA) where duplicate references identified by the software were excluded. The screening process involved two levels conducted independently by two researchers. At the first level (screening), the titles and abstracts were read to identify possible eligible studies according to the inclusion criteria. In the second level (eligibility), the entire texts were read to identify the studies to be included in the scoping review. The discrepancies between the two researchers, regarding the eligibility and inclusion of the articles, were resolved by a third researcher when necessary.

### 2.9. Data Extraction

The data of the articles were extracted into an Excel^®^ (Microsoft©, Redmond, WA, USA) spreadsheet, by two independent reviewers, within a standardized model specific to the study and the following data were extracted: (1) year of publication, first author and country; (2) study design and purpose; (3) groups (HIV infection, and comparison groups if investigated), sample size, sex and mean age; (4) health-related physical fitness component(s) investigated, method/protocol and cut-points for classification; (5) approach applied to physical activity (e.g., moderator to group differences or to association with health-related physical fitness components).

### 2.10. Data Analysis

The data from the Excel^®^ (Microsoft©, Redmond, WA, USA) spreadsheet was extracted to the R© 4.2.1 (The R Foundation for Statistical Computing, Vienna, Austria) software and coded to tabulate the results and to perform the data analysis. Data was organized first to describe the group of studies through the identification of the year, first author and country were the division proposed by the United Nations Children’s Foundation (UNICEF) [58] was applied to identify the investigated populations. Then, the study design and purpose were identified and the information regarding the sample characteristics such as groups investigated, sample size, sex and age group were analyzed. After that, the health-related physical fitness components, method/protocol, and cut-point applied were investigated. Then, studies that researched health-related physical fitness and physical activity were surveyed to identify the approach that has been given to physical activity and to verify the influence of the level of physical activity on the results found in relation to health-related physical fitness.

### 2.11. Presentation of Results

The process of identification and inclusion of the studies was presented in a flowchart following the JBI and PRISMA-ScR recommendations [56,57]. The results were organized to describe which health-related physical fitness components were investigated along the timeline of investigation, as well as the methods/protocols and cut-points applied, and to identify if the physical activity level has influenced the results found regarding the health-related physical fitness.

## 3. Results

### 3.1. Search

**Figure 1:**
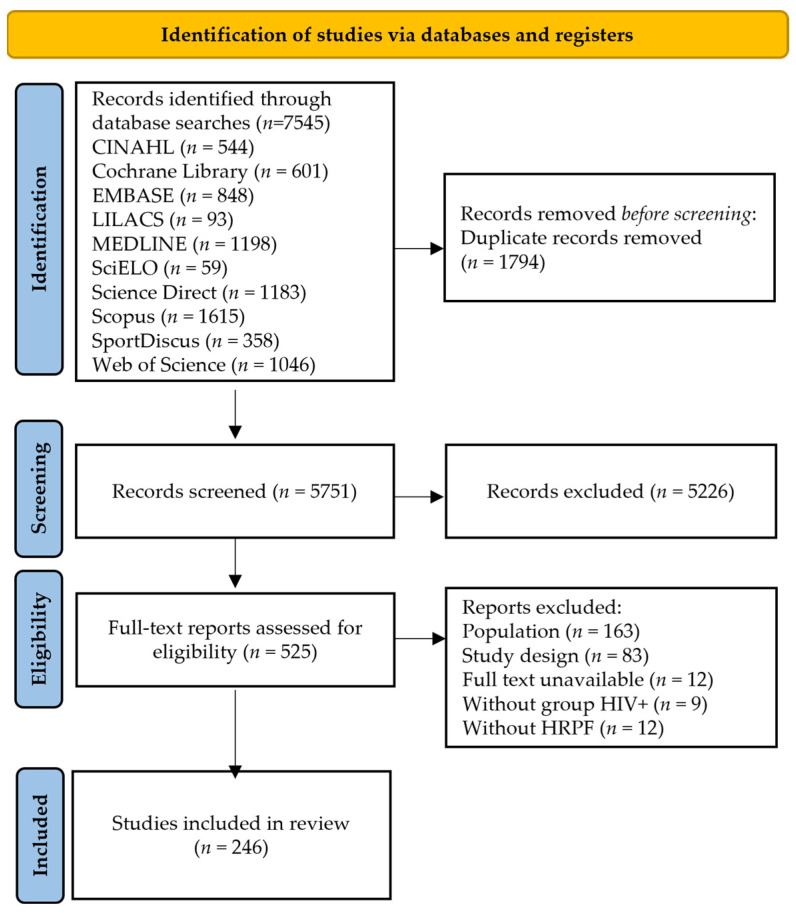
Results of database search and flowchart of the process of studies selection and inclusion. HIV+ group: diagnosed with HIV infection group; HRPF: health-related physical fitness.

The initial database search resulted in 7545 studies. Following the removal of duplicate studies (1794), the titles and abstracts of 5751 studies were read. Among these, 5226 studies were excluded due to not meeting the inclusion criteria. Therefore, the full text of 525 studies were assessed. Among these, 163 studies were excluded for investigating a sample with mean age greater than 19 years, while 83 studies were excluded due to the study design (reviews, conference abstracts, theses and dissertations). Additionally, nine studies were excluded as they did not involve samples with a diagnosis of HIV infection, and 12 were excluded because they did not investigate health-related physical fitness components. Furthermore, 12 studies were excluded due to unavailability of access to the full text. As a result, a total of 246 studies were included in the scoping review (Figure 1).

### 3.2. Characteristic of Studies (Period of Publication, Regions, Design, Studies Purpose, and Samples)

Regarding the publication period of the studies, the first study that investigated the topic of interest in the scoping review was published in 1995. The number of publications over the years showed increasing behavior with the publication of eight studies between 1995 and 1999 (less than two studies per year), 80 studies between the years 2000 and 2009 (eight studies per year), 104 studies between 2010 and 2019 (average of 10.4 studies per year) and 54 studies from 2020 to 2023 (average of 13.5 studies per year) (Table 1).

Populations from North America and Western and Central Europe, Latin America and the Caribbean, Sub-Saharan Africa, Asia and the Pacific were represented among the 246 studies. The North America and Central and Western Europe were the most represented populations among the studies, represented in 132 studies (50.0%), of which 71 studies represented the United States population and 34 represented the Italian population. Populations from Latin America and the Caribbean were represented in 65 studies (24.6%), with the Brazilian population being represented in 48 studies. Sub-Saharan African populations were represented by 50 studies (18.9%), of which 30 studies represented the South African population. Asian and Pacific populations were represented in 17 studies (6.4%), with the Thai population represented in 11 of these studies (Table 1).

Regarding the study design, 154 analytic studies and 92 descriptive studies (62.6% and 37.4%, respectively) were identified. Of those, 56.5% (*n* = 139 studies) aimed to investigate associations related to at least one of the health-related physical fitness components, 17.91% (*n* = 44 studies) investigated differences between HIV-diagnosed children and adolescents and their peers without a diagnosis of HIV infection, 15.0% (*n* = 37 studies) investigated the effects of medications ART-related or therapies complementary to ART, 5.3% (*n* = 13 studies) investigated the validation of methods to investigate the health-related physical fitness components, 3.7% (*n* = 9 studies) investigated prevalences related to at least one of the health-related physical fitness components and 1.6% (*n* = 4 studies) investigated the effects of interventions on health-related physical fitness components and/or on the physical activity level of HIV-diagnosed children and adolescents (Table 1).

Regarding the sample size of participants diagnosed with HIV infection, the study with the smallest sample investigated six participants and the study with the largest sample investigated 826 participants. Most of the studies investigated children and adolescents aged two to nineteen of both sexes (male and female), with only one study investigating only females. However, twelve studies did not report the sex of the participants (Supplementary Table S1, Supplementary Materials).

### 3.3. Health-Related Physical Fitness Components

Body composition was the health-related physical fitness component most investigated, being present in 99.2% of the studies (*n* = 244) and was mostly investigated through anthropometry (weight, height and body mass index) (*n* = 235). However, studies that used laboratory methods such as dual emission X-ray absorptiometry (DXA) (*n* = 127), computed tomography (*n* = 7), ultrasonography (*n* = 5), deuterium dilution (*n* = 4) and air displacement plethysmography (*n* = 3) were also identified. Muscular strength/endurance, the second most investigated component, was assessed in 9.3% of the studies (*n* = 23) through isokinetic isometry, vertical or horizontal jump tests and maximum repetition tests (e.g., abdominal resistance test and elbow flexion and extension test). Cardiorespiratory fitness was investigated in 6.1% of the studies (*n* = 15), through laboratory tests of maximal and submaximal effort (on treadmill or cycle ergometer tests) with breath-to-breath gas exchange analysis, as well as field tests (running or walking tests) for subsequent estimation of cardiorespiratory fitness. Flexibility was investigated in 2.4% of the studies (*n* = 4), through the sit-to-reach test and in two studies through the modified sit-to-reach test (Table 2).

Regarding the cut-points applied to classify the body composition, the use of international reference values, such as the World Health Organization (WHO) growth curves and national surveys, was observed in 149 studies. However, the use of internal cut-points (e.g., standard deviations) was observed in 30 studies. Moreover, 16 studies used cut-points developed through studies with small populations without HIV infection diagnosis. Additionally, one study developed a new cut-point for identifying lipodystrophy in HIV-diagnosed children and adolescents through anthropometry and four studies did not describe the applied cut-point (Table 3 and Table S2).

Regarding the cardiorespiratory fitness, the use of cut-points from three different assessment batteries for large populations was observed, as well as cut-points developed through two studies with small populations without HIV infection diagnosis (Table 3).

Concerning muscular strength/endurance and flexibility, the use of the same cut-points from five different assessment batteries for large populations was observed. However, one of the studies used the cut-points of three batteries without identifying which cut-point of each battery was applied to each component (Table 3).

### 3.4. Investigation of Physical Activity

Physical activity was also investigated in 20.3% of all studies (*n* = 50), in which the first study was published in 2002, through structured questionnaires (*n* = 33), accelerometers (*n* = 10) and pedometers (*n* = 2). However, five studies did not report the method. Regarding physical activity level, the WHO physical activity recommendations were applied as reference value in ten studies for classify the physical activity level. Internals cut-points, such as the use of standard deviations, were applied in 12 studies. Furthermore, six studies did not report the applied cut-point and 20 studies did not classify the physical activity level. Physical activity was investigated through different purposes such as group comparisons (*n* = 21), associations with health-related physical fitness components (*n* = 12) and models adjustments (*n* = 8). Where six studies observe no association between physical activity level and body fat and bone mass parameters. However, four studies observe negative association between physical activity level and fat mass parameters, two studies observe positive association between physical activity level and bone mass parameters, one study observes positive association between physical activity level and fat-free mass and one study observes positive association between physical activity level and muscle strength/endurance. In addition to that, the results of eight studies that adjusted models’ analysis through physical activity level did not change (Supplementary Materials Table S3).

## 4. Discussion

The relationship between body composition and the HIV infection/ART use has been the studies main focus, since the beginning of the health-related physical fitness components investigation in HIV-diagnosed children and adolescents, primarily to understand alterations in growth pattern and nutritional status [7,8,9,10,11,12,13,14,15,16,17] and most recently to investigate modifications in body composition such as changes in fat mass distribution [18], alterations in bone mass [19,20] and reduction in muscle mass [21]. In the early 2000s, research began on the cardiorespiratory fitness [22,23], followed by the beginning of the research into muscular strength/endurance in the mid-2000s [27,28] and the beginning of the research into flexibility in 2010 [28], with the aim to investigate the relationship between the HIV infection/ART use and reduce cardiorespiratory fitness [22,23], low muscular strength/endurance [27,28] and flexibility [28]. In addition to the investigation of different health-related physical fitness components over time, the number of publications over the years has shown increasing behavior for the four health-related physical fitness components. These facts demonstrate changes in the investigation of the health-related physical fitness components with new perspectives over the years, and increased interest in investigating the different health-related physical fitness components in HIV-diagnosed children and adolescents. However, when observed the proportions of studies that investigate muscular strength/endurance, cardiorespiratory fitness, and flexibility in comparison with proportion of studies that investigate body composition, it shows that the investigation of those health-related physical fitness components has been scarcely explored in HIV-diagnosed children and adolescents.

Regarding the methods/protocols and cut-points applied to investigate the health-related physical fitness components in HIV-diagnosed children and adolescents, the use of reference methods to investigate the different health-related physical fitness components was highlighted [53,59,60,61], as well as alternatives field tests that are more accessible for use in epidemiological studies [53,62,63]. However, only 13 studies that aimed to validate methods for investigating the health-related physical fitness components were identified [32,34,35,64,65,66,67,68,69,70,71,72,73], of which 12 studies investigated the validation of methods for assessing body composition [32,35,64,65,66,67,68,69,70,71,72,73]. Furthermore, studies that proposed reference values for evaluating health-related physical fitness were not identified. Considering that the method validation process aims to identify their usability to evaluate the investigated variables ensuring the accuracy of the collected measurements, as well as suitability for the investigated population [74,75] and that the process of proposing reference values aims to elucidate parameters related to factors such as health indicators [76,77]. Despite the use of different pre-established reference values, such as the WHO growth curves [78], and the use of reference values from protocols which were developed aiming at global health parameters [79,80]. Studies that aimed to investigate the relationship between reference values and specific health indicators for the population of HIV-infected children and adolescents, such as viral load, CD4 and CD8 lymphocyte counts and immunosuppression status [81,82] were not identified. Furthermore, the use of reference values still in the consolidation phase was observed [83]. Thus, the lack of validation studies that aim to identify the usability of methods/protocols, as well as studies that aim to propose reference values and/or to verify the suitability of pre-established reference values for the investigation of the health-related physical fitness components of HIV-diagnosed children and adolescents was evidenced.

Observing the aim of the studies, the results of the present scoping review reflect a broad descriptive investigation of differences between HIV-diagnosed populations and HIV non diagnosed peers [11,19,21,23,24,25,26,29,30,38,51,73,84,85,86,87,88,89,90,91,92,93,94,95,96,97,98,99,100,101,102,103,104,105,106,107,108,109,110,111,112,113,114,115,116], prevalences related to health-related physical fitness components [12,117,118,119,120,121,122,123,124] and different associations [7,8,10,14,15,18,20,22,27,31,33,36,37,39,40,41,42,43,45,47,125,126,127,128,129,130,131,132,133,134,135,136,137,138,139,140,141,142,143,144,145,146,147,148,149,150,151,152,153,154,155,156,157,158,159,160,161,162,163,164,165,166,167,168,169,170,171,172,173,174,175,176,177,178,179,180,181,182,183,184,185,186,187,188,189,190,191,192,193,194,195,196,197,198,199,200,201,202,203,204,205,206,207,208,209,210,211,212,213,214,215,216,217,218,219,220,221,222,223,224,225,226,227,228,229,230,231,232,233,234,235,236,237,238,239,240,241,242,243] between health-related physical fitness components and variables such as the HIV infection status (viral load, CD4 and CD8 lymphocyte count and immunosuppression status) [8,45,134,150] and use of different ART regimens [19,124,194,244,245]. Moreover, the proportions of studies demonstrated a lack of studies to possibly under-stand the cause of changes in the health-related physical fitness components in HIV-diagnosed children and adolescents where it is observed that only 15.0% of the studies aimed to investigate possible changes in the health-related physical fitness components related to the ART use, or ART related therapies [9,13,16,17,38,44,46,244,245,246,247,248,249,250,251,252,253,254,255,256,257,258,259,260,261,262,263,264,265,266,267,268,269,270,271,272,273] and 1.7% of the studies aimed to investigate effects related to interventions that aimed to improve health-related physical fitness components and/or the physical activity level in HIV-diagnosed children and adolescents [28,50,52,274].

In addition to that, studies that aimed to investigate the relationship between HIV infection/ART use and the health-related physical fitness components showed changes in the body fat distribution [91,104,194,264], reduction in the bone mass [15,84,108,124,209,262] and fat-free mass [8,102]. However, only 12.2% (*n* = 30) of the studies investigated more than one the health-related physical fitness component [21,22,23,24,25,26,27,28,29,30,31,33,34,41,47,50,52,106,110,112,116,192,196,221,222,223,226,230,233,238] and no study that investigated the relationship between the health-related physical fitness components was found. Muscular strength/endurance is directly associated with the amount of muscle fibers—that is, related to the amount of fat-free mass, specifically muscle tissue, as well as cardiorespiratory fitness—is directly associated with the amount of type I (oxidative) muscle fibers [48,49]. Investigating a certain health-related physical fitness component without investigating other related component may be neglecting relevant moderating factors of the relationships investigated, such as the interdependent relationships between the different components. A lack of knowledge was shown in terms of the relationship between the health-related physical components, such as an example how much changes in body composition represent in reduction in muscular strength/endurance, cardiorespiratory fitness and flexibility.

Regarding the regions in which the studies where developed, the estimate of HIV-diagnosed children and adolescents in 2022 was approximately 2.6 million, of which 84.6% (2.2 million) were from the Sub-Saharan Africa, 3.8% (99,000) from Asia and the Pacific and 3.3% (85,000) from the Latin America and the Caribbean [58]. Although all the regions across the globe were represented [58], the proportion of populations investigated in the studies does not reflect the most representative populations of HIV-diagnosed children and adolescents across the globe when its observed that the regions that presented the highest proportion of studies (North America and Western and Central Europe, with 50.0% of the studies) and the regions that presented the lowest proportion of studies (Sub-Saharan Africa and Asia and the Pacific, with 18.9% and 6.4%, respectively).

Physical activity was investigated through different aims such as to investigate differences between HIV-diagnosed group compared to non-diagnosed groups [19,21,25,29,31,40,51,87,88,95,98,99,100,106,108,109,112,115,143,198,204,234], to investigate the association between health-related physical fitness and physical activity level [18,29,33,42,95,99,100,124,197,210,227,238] and to adjust models through physical activity [18,36,37,42,230,231,233,238]. In addition to the assumption that improvements in physical activity level can result in improvements in health-related physical fitness components [48,49,53], it follows that physical activity can moderate the relationship between HIV infection/ART use and the health-related physical fitness components, as well the differences between HIV-diagnosed children and adolescents and their peers without IV infection diagnosis [48,49,53]. The twelve studies that investigated the association between health-related physical fitness and physical activity presented divergent results, either indicating association [18,29,33,95,99,124,227,238], or showing the absence of association [42,99,100,197,210,227,239]. Moreover, studies that used the physical activity level to adjust models observe that if physical activity was moderating the investigated relationships, no difference was shown in the results after the adjustments through physical activity [18,36,37,42,230,231,233,238]. Those inconclusive results regarding the relationship between health-related physical fitness components and physical activity can be related to the use of different methods to investigate physical activity. Where the use of direct methods [25,31,238], considered reference methods to investigate the physical activity level [53,62,63], as well as indirect methods [21,112,115], considered as alternatives methods that are more accessible for epidemiological studies [53,62,63], was observed. Moreover, physical activity was classified using different reference values and cut-points. Where the WHO Guidelines on Physical Activity and Sedentary Behavior was applied in 20% (*n* = 10) of the studies, however, 24% (*n* = 12) applied internal cut-points, 12% (*n* = 6) did not report the cut-point applied. In addition to that, the physical activity level investigation through structured questionaries that did not report usability was observe in most of the studies, and only one study investigated the usability of structed questionaries and proposed cut-points to estimate the physical activity level in HIV-diagnosed children and adolescents [275]. In addition to the lack of validation studies that aim to identify the usability of methods/protocols, the lack of standardization between studies in terms of investigating the level of physical activity was also observed. Thus, it is difficult to make a comparison between these studies and interpret their findings in the literature. Another fact that can be related to the inconclusive results regarding the relationship between health-related physical fitness components and physical activity is the participants physical activity level itself. It can be observed that although almost half of the studies showed no difference between HIV-diagnosed participant and HIV-non diagnosed participants and the other half showed lower physical activity level form HIV-diagnosed participants, one fact in common to the studies is the participants low physical activity level [29,31,36,112,115]. Thus, the physical activity level can be insufficient for in improvements in health-related physical fitness components, and will not work as a moderator of the relationship between health-related physical fitness components and the investigated variables [48,49,53].

Concerning the studies that aimed to investigate differences between HIV-diagnosed children and adolescents and their peers without diagnosis, part of these studies adopted matching strategies such as matching by sex and age to ensure data quality [25,26]. The importance of adopting matching strategies lies in the fact that the differences between HIV-diagnosed children and adolescents when compared to their peers without HIV infection diagnosis, as well as the relationships between the health-related physical fitness components with the investigated variables, could be explained by variables that can moderate these differences and relationships such as sex and the physical activity level [53,59,60,61]. The relevance of adopting these matching strategies was evidenced in this scoping review considering that females presented higher values of fat mass percentage, body mass component [32] and in the flexibility test [31] when compared to males, and that males presented higher values of cardiorespiratory fitness [25], muscular strength/endurance [31] and physical activity level [31,275] when compared to females. However, the results from these scoping reviews show the lack of knowledge in terms of understanding the importance of the physical activity level as a moderating factor in the relationship between HIV infection/ART use and the health-related physical fitness components in HIV-diagnosed children and adolescents in terms of the physical activity level investigation [48,49,53]. Another limitation of the interpretation of these studies is the difference between HIV-diagnosed children and adolescents when compared whit their peers without a diagnosis of HIV infection [54,55].

Despite this, this scoping review has limitations, such as the fact that the literature search did not include the gray literature, such as theses and dissertations, as it was not part of the search and inclusion process. The adoption of this strategy was due to the fact that, although gray literature can highlight the findings of emerging research and reflect the updated panorama of the topic investigated, it can, in terms of quality, present methodological flaws which can interfere in the reliability and interpretation of the results found [276,277].

## 5. Conclusions

Through the results of this scoping review, it is concluded that the relationship between body composition and the HIV infection and use of ART has been the primarily focus of most studies. However, research on muscular strength/endurance, cardiorespiratory fitness and flexibility has been scarcely explored. Regarding the methods/protocols and cut-points applied to investigate the health-related physical fitness components in HIV-diagnosed children and adolescents, the lack of studies that investigated methods usability as well as reference values was evidenced. Moreover, a lack of studies to possibly understand the causal relationship of alterations on the health-related physical fitness components in HIV-diagnosed children and adolescents was observed. Additionally, the regions with the highest prevalence of HIV-diagnosed children and adolescents were the least investigated. Therefore, the results found may not be reflecting the population across the globe in a generalized way, but rather reflecting small populations. Regarding the studies that investigated the health-related physical fitness components and the physical activity level, it is not clear if the physical activity level should be investigated as a moderator factor to the investigated relationships between health-related physical fitness and the research variables. It was also evident that the investigation of differences between HIV-diagnosed children and adolescents and their peers must be associated with matching strategies that aim to mitigate the influence of variables that moderate the prevalence and relationships investigated. Thus, thought the scoping review finds the following need for future research directions is presented: (I) to develop studies to investigate cardiorespiratory fitness, muscular strength/endurance and flexibility; (II) to develop studies to investigate methods usability as well as reference values for this population; (III) to develop studies to possibly understand the causal relationship of alterations on the health-related physical fitness components in HIV-diagnosed children and adolescents; (IV) to develop studies in the regions with the highest prevalence of HIV-diagnosed children and adolescents; (V) to develop studies to possibly understand if physical activity level can be investigated as a moderator factor to the relationships between health-related physical fitness and the research variables.

## Figures and Tables

**Table 1:** Characteristics of the included studies (period of publication, regions, design and studies purpose).

**Period of Publication**	**Total of Studies (*n* = 246)**	**Average Studies per Year**
1995 to 1999	8	1.6
2000 to 2009	80	8.0
2010 to 2019	104	10.4
2020 to May 2023	54	13.5
**Region (UNICEF division)**	**Total of studies per region**	**% of total studies per region (*n* = 264)**
North America and Western and Central Europe	132	50.0
Latin America and the Caribbean	65	24.6
Sub-Saharan Africa	50	18.9
Asia and the Pacific	17	6.4
**Study design**	**Total of studies**	**% of total studies** **(*n* = 246)**
Descriptive studies	92	37.4
Analytic studies	154	62.6
**Propose of studies (to investigate)**	**Total of studies**	**% of total studies** **(*n* = 246)**
Associations	139	56.5
Differences between HIV+ and HIV−	44	17.9
ART-related effects (with or without controls)	37	15.0
Method validity	13	5.3
Prevalences	9	3.7
Interventions effects	4	1.6

UNICEF: United Nations Children’s Foundation; %: percentage; HIV+: HIV-diagnosed; HIV−: without HIV infection diagnosis; ART: antiretroviral therapy.

**Table 2:** Protocols/tests applied to investigate health-related physical fitness components.

Component (Method/Protocol)	Total of Studies	% of Total Studies (*n* = 246)
Body composition	*n* = 244	99.2%
Anthropometry	235	
Dual emission X-ray absorptiometry	127	
Bioelectrical impedance analysis	21	
Computed tomography	7	
Ultrasonography	5	
Deuterium dilution	4	
Visual inspection	2	
Air Displacement Plethysmography	3	
X-ray	1	
Muscular strength/endurance	*n* = 23	9.3%
Handgrip strength	11	
Abdominal resistance test	3	
Horizontal jump test	3	
Vertical jump test	3	
Handheld dynamometer	2	
Isokinetic isometry	1	
1-maximum repetition	1	
Sit-up	1	
Hand hang resistance test	1	
Respiratory strength	1	
Push-ups test	1	
Cardiorespiratory fitness	*n* = 15	6.1%
Maximal effort treadmill test	4	
Maximal effort cycle ergometer tests	4	
Submaximal effort treadmill test	1	
Six-minutes walking test	3	
20 m shuttle run test	2	
Incremental waling test	1	
Flexibility	*n* = 6	2.4%
Sit-to-reach test	4	
Modified sit-to-reach test	2	

%: percentage.

**Table 3:** Reference values and cut-points applied to investigate health-related physical fitness components.

**Body Composition**	***n* = 244**	% *
NCHS/WHO growth curves	88	36.1%
Nationals’ growth curves	34	13.9%
Z-scores	22	9.0%
Previous study (HIV-sample)	16	6.6%
NHANES	14	5.7%
Percentiles	7	2.7%
Ten-State Nutrition Survey	4	1.6%
Not reported	4	1.6%
Osteoporosis WHO taskforce	3	1.2%
United States BMD in Childhood Study	3	1.2%
International Society for Clinical Densitometry	3	1.2%
New cut-point	1	0.4%
Terciles	1	0.4%
Not applied	42	17.2%
**Muscular strength/endurance**	***n* = 23**	
Z-scores	3	13.0%
Previous study (HIV-sample)	1	4.3%
PROESP-BR	1	4.3%
National Presidential Fitness Program	1	4.3%
Different batteries *	1	4.3%
Not reported	2	8.7%
Not applied	11	47.8%
**Cardiorespiratory fitness**	***n* = 15**	
Previous study (HIV-sample)	2	13.3%
ACSM guidelines	1	6.6%
National Presidential Fitness Program	1	6.6%
American Thoracic Society	1	6.6%
Not applied	8	53.3%
**Flexibility**	***n* = 6**	
PROESP-BR	1	16.7%
National Presidential Fitness Program	1	16.7%
Different batteries **	1	16.7%
Not applied	3	50.0%

%: percentage; NCHS: National Center for Health Statistics; WHO: World Health Organization; HIV−: without human immunodeficiency virus infection diagnosis; NHANES: National Health and Nutrition Examination Survey; BMD: bone mass density; PROESP-BR: Projeto Esporte Brasil; ACSM: American College of Sports Medicine. * Percentage of studies regarding each health-related physical fitness component. ** Use of three batteries without identifying which cut-point of each battery was applied to each component.

## Data Availability

No new data were created or analyzed in this study. Data sharing is not applicable to this article.

## Supplementary Materials

The following supporting information can be downloaded at: https://www.mdpi.com/article/10.3390/ijerph21050541/s1, Supplementary Table S1: Publication year, first author, country, design, purpose, and participants of the studies included in the scoping review; Supplementary Table S2: Health-related physical fitness components investigated and physical activity level (protocols/tests and cut-points applied); Supplementary Table S3: Physical activity investigation: methods/protocols, reference values and cut-points, aims and outcomes. Supplementary Table S4: References included in the scoping review.
