# Supplementary material for: Health-Related Physical Fitness Evaluation in HIV-Diagnosed Children and Adolescents: A Scoping Review
Source: Int J Environ Res Public Health. 2024 Apr 25;21(5):541. doi: 10.3390/ijerph21050541 (PMC11121308; doi:10.3390/ijerph21050541)
Supplement: Supplementary file 1 [file ijerph-21-00541-s001.zip › ijerph-2940752-supplementary.pdf]

SEARCH STRATEGY

DATABASES
1 - Cochrane
2 - MEDLINE (via PubMed)
3 - EMBASE (via Ovid)
4 - Web of Science
5 - SportDiscus (via EBSCOhost)
6 - LILACS (via BVS)
7 - Scopus
8 - SciELO
9 - Science Direct
10 – CINAHL

DESCRIPTORS
First block (concept) – Health-related physical fitness components (cardiorespiratory fitness; muscle strength; body composition and flexibility).

Cardiorespiratory fitness
“cardiorespiratory fitness”; “oxygen consumption”; “aerobic capacity”; “aerobic fitness”; “cardiorespiratory capacity”; “cardiovascular fitness”; “aerobic power”; “aerobic endurance”; “cardiorespiratory endurance”; “maximum oxygen consumption”; “maximal oxygen uptake”; “VO2 maximal”;

Muscle strength
"muscle strength"; "muscle contraction"; "isometric contraction"; "isotonic contraction"; "resistance training"; "muscular power"; “endurance training”; "upper limb strength"; "lower limb strength"; "muscular endurance"; "musculoskeletal fitness"; "muscular fitness"; “explosive strength”;

Body composition
“body composition”; “body composition analysis”; “adipose tissue”; “bone density”; “bone mass”; “mineral mass”; “fat mass”; “fat free mass”; “lean body mass”; “functional body composition”;

Flexibility
“flexibility”; “range of motion”; “muscle stretching exercises”;

Second block (context) - HIV infection diagnosis.

“HIV infections”; “HIV”; “human immunodeficiency virus”; “HIV seropositivity”; “HIV testing”; “acquired immunodeficiency syndrome”; “AIDS”;

Third block (population) - Children and adolescents (5 to 19 years old).

“child”; “children”; “adolescent”; “adolescents”; “youth”; “young”; “teen”; “teenager”; “preschool child”; “boys”; “girls”; “pediatric”;

Search strategy for each database

1 - Cochrane (https://www.cochranelibrary.com/advanced-search)

- Start: 05/01/2024 End: 05/01/2024
- Total: 601 (Trials)

Filters: All Text; Trials

((“physical fitness” OR “health-related physical fitness” OR “cardiorespiratory fitness” OR “oxygen consumption” OR “aerobic capacity” OR “aerobic fitness” OR “cardiorespiratory capacity” OR “cardiovascular fitness” OR “aerobic power” OR “aerobic endurance” OR “cardiorespiratory endurance” OR “maximum oxygen consumption” OR “maximal oxygen uptake” OR “VO2 maximal” OR "muscle strength" OR "muscle contraction" OR "isometric contraction" OR "isotonic contraction" OR "resistance training" OR "muscular power" OR “endurance training” OR "upper limb strength" OR "lower limb strength" OR "muscular endurance" OR "musculoskeletal fitness" OR "muscular fitness" OR “explosive strength” OR “body composition” OR “body composition analysis” OR “adipose tissue” OR “bone density” OR “bone mass” OR “mineral mass” OR “fat mass” OR “fat free mass” OR “lean body mass” OR “functional body composition” OR flexibility OR “range of motion” OR “muscle stretching exercises”)) AND ((“HIV infections” OR HIV OR “human immunodeficiency virus” OR “HIV seropositivity” OR “HIV testing” OR “acquired immunodeficiency syndrome” OR AIDS)) AND ((child OR children OR adolescent OR adolescents OR youth OR young OR teen OR teenager OR “preschool child” OR boys OR girls OR pediatric))

2 - MEDLINE (via PubMed) (https://pubmed.ncbi.nlm.nih.gov/advanced/)

- Start: 05/01/2024   End: 05/01/2024
- Total: 1.198

Filters: None.

((“physical fitness” OR “health-related physical fitness” OR “cardiorespiratory fitness” OR “oxygen consumption” OR “aerobic capacity” OR “aerobic fitness” OR “cardiorespiratory capacity” OR “cardiovascular fitness” OR “aerobic power” OR “aerobic endurance” OR “cardiorespiratory endurance” OR “maximum oxygen consumption” OR “maximal oxygen uptake” OR “VO2 maximal” OR "muscle strength" OR "muscle contraction" OR "isometric contraction" OR "isotonic contraction" OR "resistance training" OR "muscular power" OR “endurance training” OR "upper limb strength" OR "lower limb strength" OR "muscular endurance" OR "musculoskeletal fitness" OR "muscular fitness" OR “explosive strength” OR “body composition” OR “body composition analysis” OR “adipose tissue” OR “bone density” OR “bone mass” OR “mineral mass” OR “fat mass” OR “fat free mass” OR “lean body mass” OR “functional body composition” OR “flexibility” OR “range of motion” OR “muscle stretching exercises”) AND (“HIV infections” OR “HIV” OR “human immunodeficiency virus” OR “HIV seropositivity” OR “HIV testing” OR “acquired immunodeficiency syndrome” OR “AIDS”)) AND (“child” OR “children” OR “adolescent” OR “adolescents” OR “youth” OR “young” OR “teen” OR “teenager” OR “preschool child” OR “boys” OR “girls” OR “pediatric”)

3 - EMBASE (via Ovid) (https://www.embase.com/#advancedSearch/default)

- Start: 05/01/2024 End: 05/01/2024
- Total: 848

Filters: [embase]/lim NOT ([embase]/lim AND [medline]/lim).

('physical fitness'/exp OR 'physical fitness' OR 'health-related physical fitness' OR 'cardiorespiratory fitness'/exp OR 'cardiorespiratory fitness' OR 'oxygen consumption'/exp OR 'oxygen consumption' OR 'aerobic capacity'/exp OR 'aerobic capacity' OR 'aerobic fitness'/exp OR 'aerobic fitness' OR 'cardiorespiratory capacity' OR 'cardiovascular fitness'/exp OR 'cardiovascular fitness' OR 'aerobic power'/exp OR 'aerobic power' OR 'aerobic endurance' OR 'cardiorespiratory endurance'/exp OR 'cardiorespiratory endurance' OR 'maximum oxygen consumption'/exp OR 'maximum oxygen consumption' OR 'maximal oxygen uptake'/exp OR 'maximal oxygen uptake' OR 'vo2 maximal' OR 'muscle strength'/exp OR 'muscle strength' OR 'muscle contraction'/exp OR 'muscle contraction' OR 'isometric contraction'/exp OR 'isometric contraction' OR 'isotonic contraction'/exp OR 'isotonic contraction' OR 'resistance training'/exp OR 'resistance training' OR 'muscular power'/exp OR 'muscular power' OR 'endurance training'/exp OR 'endurance training' OR 'upper limb strength' OR 'lower limb strength'/exp OR 'lower limb strength' OR 'muscular endurance' OR 'musculoskeletal fitness' OR 'muscular fitness'/exp OR 'muscular fitness' OR 'explosive strength' OR 'body composition'/exp OR 'body composition' OR 'body composition analysis' OR 'adipose tissue'/exp OR 'adipose tissue' OR 'bone density'/exp OR 'bone density' OR 'bone mass'/exp OR 'bone mass' OR 'mineral mass' OR 'fat mass'/exp OR 'fat mass' OR 'fat free mass'/exp OR 'fat free mass' OR 'lean body mass'/exp OR 'lean body mass' OR 'functional body composition' OR 'flexibility'/exp OR 'flexibility' OR 'range of motion'/exp OR 'range of motion' OR 'muscle stretching exercises'/exp OR 'muscle stretching exercises') AND ('hiv infections' OR 'hiv' OR 'human immunodeficiency virus' OR 'hiv seropositivity' OR 'hiv testing' OR 'acquired immunodeficiency syndrome' OR 'aids') AND ('child' OR 'children' OR 'adolescent' OR 'adolescents' OR 'youth' OR 'young' OR 'teen' OR 'teenager' OR 'preschool child' OR 'boys' OR 'girls' OR 'pediatric') AND [embase]/lim NOT ([embase]/lim AND [medline]/lim)

4 - Web of Science
(https://www-webofscience.ez46.periodicos.capes.gov.br/wos/woscc/basic-search)

- Start: 05/01/2024 End: 05/01/2024
- Total: 1046

Filters: None.

((ALL=(“physical fitness” OR “health-related physical fitness” OR “cardiorespiratory fitness” OR “oxygen consumption” OR “aerobic capacity” OR “aerobic fitness” OR “cardiorespiratory capacity” OR “cardiovascular fitness” OR “aerobic power” OR “aerobic endurance” OR “cardiorespiratory endurance” OR “maximum oxygen consumption” OR “maximal oxygen uptake” OR “VO2 maximal” OR "muscle strength" OR "muscle contraction" OR "isometric contraction" OR "isotonic contraction" OR "resistance training" OR "muscular power" OR “endurance training” OR "upper limb strength" OR "lower limb strength" OR "muscular endurance" OR "musculoskeletal fitness" OR "muscular fitness" OR “explosive strength” OR “body composition” OR “body composition analysis” OR “adipose tissue” OR “bone density” OR “bone mass” OR “mineral mass” OR “fat mass” OR “fat free mass” OR “lean body mass” OR “functional body composition” OR “flexibility” OR “range of motion” OR “muscle stretching exercises”)) AND ALL=(“HIV infections” OR “HIV” OR “human immunodeficiency virus” OR “HIV seropositivity” OR “HIV testing” OR “acquired immunodeficiency syndrome” OR “AIDS”)) AND ALL=(“child” OR “children” OR “adolescent” OR “adolescents” OR “youth” OR “young” OR “teen” OR “teenager” OR “preschool child” OR “boys” OR “girls” OR “pediatric”)

5 - SportDiscus (via EBSCOhost)
(https://web-p-ebscohost.ez46.periodicos.capes.gov.br/ehost/search/basic?vid=0&sid=7f93a08b-1892-43f3-bac9-0e762a94d9ef%40redis)

- Start: 05/01/2024 End: 05/01/2024
- Total: 358

Filters: (*Expanders - Apply equivalent subjects; Search Modes - Boolean/Phrase).*

TX ((“physical fitness”) OR (“health-related physical fitness”) OR (“cardiorespiratory fitness”) OR (“oxygen consumption”) OR (“aerobic capacity”) OR (“aerobic fitness”) OR (“cardiorespiratory capacity”) OR (“cardiovascular fitness”) OR (“aerobic power”) OR (“aerobic endurance”) OR (“cardiorespiratory endurance”) OR (“maximum oxygen consumption”) OR (“maximal oxygen uptake”) OR (“VO2 maximal”) OR ("muscle strength") OR ("muscle contraction") OR ("isometric contraction") OR ("isotonic contraction") OR ("resistance training") OR ("muscular power") OR (“endurance training”) OR ("upper limb strength") OR ("lower limb strength") OR ("muscular endurance") OR ("musculoskeletal fitness") OR ("muscular fitness") OR (“explosive strength”) OR (“body composition”) OR (“body composition analysis”) OR (“adipose tissue”) OR (“bone density”) OR (“bone mass”) OR (“mineral mass”) OR (“fat mass”) OR (“fat free mass”) OR (“lean body mass”) OR (“functional body composition”) OR (“flexibility”) OR (“range of motion”) OR (“muscle stretching exercises”)) AND ((“HIV infections”) OR (“HIV”) OR (“human immunodeficiency virus”) OR (“HIV seropositivity”) OR (“HIV testing”) OR (“acquired immunodeficiency syndrome”) OR (“AIDS”)) AND ((“child”) OR (“children”) OR (“adolescent”) OR (“adolescents”) OR (“youth”) OR (“young”) OR (“teen”) OR (“teenager”) OR (“preschool child”) OR (“boys”) OR (“girls”) OR (“pediatric”))

6 - LILACS (via BVS) https://bvsalud.org/

- Start: 05/01/2024   End: 05/01/2024
- Total: 93

Filters: Title, Summary, Subject; LILACS database.

English

((“physical fitness” OR “health-related physical fitness” OR “cardiorespiratory fitness” OR “oxygen consumption” OR “aerobic capacity” OR “aerobic fitness” OR “cardiorespiratory capacity” OR “cardiovascular fitness” OR “aerobic power” OR “aerobic endurance” OR “cardiorespiratory endurance” OR “maximum oxygen consumption” OR “maximal oxygen uptake” OR “VO2 maximal”) OR ("muscle strength" OR "muscle contraction" OR "isometric contraction" OR "isotonic contraction" OR "resistance training" OR "muscular power" OR “endurance training” OR "upper limb strength" OR "lower limb strength" OR "muscular endurance" OR "musculoskeletal fitness" OR "muscular fitness" OR “explosive strength”) OR (“body composition” OR “body composition analysis” OR “adipose tissue” OR “bone density” OR “bone mass” OR “mineral mass” OR “fat mass” OR “fat free mass” OR “lean body mass” OR “functional body composition”) OR (flexibility OR “range of motion” OR “muscle stretching exercises”)) AND (“HIV infections” OR HIV OR “human immunodeficiency virus” OR “HIV seropositivity” OR “HIV testing” OR “acquired immunodeficiency syndrome” OR AIDS) AND (child OR children OR adolescent OR adolescents OR youth OR young OR teen OR teenager OR “preschool child” OR boys OR girls OR pediatric)

Portuguese

(((“aptidão física”) OR (“aptidão física relacionada a saúde”) OR (“aptidão cardiorrespiratória”) OR (“consumo de oxigênio”) OR (“capacidade aeróbica”) OR (“aptidão aeróbica”) OR (“capacidade cardiorrespiratória”) OR (“aptidão cardiovascular”) OR (“potência aeróbica”) OR (“resistência aeróbica”) OR (“resistência cardiovascular”) OR (“consumo máximo de oxigênio”) OR (“absorção máxima de oxigênio”) OR (“VO2 máximo”)) OR (("força muscular") OR ("contração muscular") OR ("contração isométrica") OR ("contração isotônica") OR ("treinamento resistido") OR ("potência muscular") OR (“treinamento resistido”) OR ("força de membros superiores") OR ("força de membros inferiores") OR ("resistência muscular") OR ("aptidão musculoesquelética") OR ("aptidão muscular") OR (“força explosiva”)) OR ((“composição corporal”) OR (“análise da composição corporal”) OR (“tecido adiposo”) OR (“densidade óssea”) OR (“massa óssea”) OR (“massa mineral óssea”) OR (“gordura corporal”) OR (“massa livre de gordura”) OR (“massa magra”) OR (“composição corporal funcional”)) OR ((flexibilidade) OR (“amplitude de movimento”) OR (“exercícios de alongamento muscular”))) AND ((“infecção por HIV”) OR (HIV) OR (“vírus da imunodeficiência humana”) OR (“HIV soropositivo”) OR (“diagnóstico de HIV”) OR (“síndrome da imunodeficiência adquirida”) OR (AIDS)) AND ((criança) OR (crianças) OR (adolescente) OR (adolescentes) OR (jovens) OR (jovem) OR (“pré-escolares”) OR (meninos) OR (meninas) OR (população pediátrica))

Spanish

((("aptitud física") OR ("aptitud física relacionada con la salud") OR ("aptitud cardiorrespiratoria") OR ("consumo de oxígeno") OR ("capacidad aeróbica") OR ("aptitud aeróbica") OR ("capacidad cardiorrespiratoria") OR ("capacidad cardiovascular") OR ("potencia aeróbica") OR ("resistencia aeróbica") OR ("resistencia cardiovascular") OR ("consumo máximo de oxígeno") OR ("consumo máximo de oxígeno") OR (" VO2 max")) OR (("fuerza muscular") OR ("contracción muscular") OR ("contracción isométrica") OR ("contracción isotónica") OR ("entrenamiento de resistencia") OR ("fuerza muscular") OR ("entrenamiento de resistencia") OR ("fuerza de miembros superiores") OR ("fuerza de miembros inferiores") OR ("resistencia muscular") OR ("aptitud musculo esquelética") OR ("aptitud muscular") OR ("fuerza explosiva")) OR (("composición corporal") OR ("análisis de la

composición corporal") OR ("tejido adiposo") OR ("densidad ósea") OR ("masa ósea") OR ("masa mineral ósea") OR ("grasa corporal") OR (“masa sin grasa”) OR (“masa magra”) OR (“composición corporal funcional”)) OR ((flexibilidad) OR (“rango de movimiento”) OR (“ejercicios de estiramiento muscular”))) AND ((“infección por VIH”) OR (VIH) OR (“virus de la inmunodeficiencia humana”) OR (“VIH seropositivo”) OR (“diagnóstico de VIH”) OR (“síndrome de inmunodeficiencia adquirida”) OR (SIDA)) AND ((niño) OR (niños) OR (adolescente) OR (adolescentes) OR (jóvenes) OR (“preescolares”) OR (niños) OR (niñas) OR (población pediátrica))

7 - Scopus
(https://www-scopus.ez46.periodicos.capes.gov.br/search/form.uri?display=basic#basic)

- Start: 05/01/2024 End: 05/01/2024
- Total: 1615

Filters: TITLE-ABS-KEY.

TITLE-ABS-KEY (("physical fitness" OR "health-related physical fitness" OR "cardiorespiratory fitness" OR "oxygen consumption" OR "aerobic capacity" OR "aerobic fitness" OR "cardiorespiratory capacity" OR "cardiovascular fitness" OR "aerobic power" OR "aerobic endurance" OR "cardiorespiratory endurance" OR "maximum oxygen consumption" OR "maximal oxygen uptake" OR "VO2 maximal" OR "muscle strength" OR "muscle contraction" OR "isometric contraction" OR "isotonic contraction" OR "resistance training" OR "muscular power" OR "endurance training" OR "upper limb strength" OR "lower limb strength" OR "muscular endurance" OR "musculoskeletal fitness" OR "muscular fitness" OR "explosive strength" OR "body composition" OR "body composition analysis" OR "adipose tissue" OR "bone density" OR "bone mass" OR "mineral mass" OR "fat mass" OR "fat free mass" OR "lean body mass" OR "functional body composition" OR flexibility OR "range of motion" OR "muscle stretching exercises") AND ("HIV infections" OR hiv OR "human immunodeficiency virus" OR "HIV seropositivity" OR "HIV testing" OR "acquired immunodeficiency syndrome" OR aids) AND (child OR children OR adolescent OR adolescents OR youth OR young OR teen OR teenager OR "preschool child" OR boys OR girls OR pediatric))

8 - SciELO (https://scielo.org/)

- Start: 05/01/2024 End: 05/01/2024
- Total: 59

Filters: None.

English

((physical fitness) OR (health-related physical fitness) OR (cardiorespiratory fitness) OR (oxygen consumption) OR (aerobic capacity) OR (aerobic fitness) OR (cardiorespiratory capacity) OR (cardiovascular fitness) OR (aerobic power) OR (aerobic endurance) OR (cardiorespiratory endurance) OR (maximum oxygen consumption) OR (maximal oxygen uptake) OR (VO2 maximal) OR (muscle strength) OR (muscle contraction) OR (isometric contraction) OR (isotonic contraction) OR (resistance training) OR (muscular power) OR (endurance training) OR (upper limb strength) OR (lower limb strength) OR (muscular endurance) OR (musculoskeletal fitness) OR (muscular fitness) OR (explosive strength) OR (body composition) OR (body composition analysis) OR (adipose tissue) OR (bone density) OR (bone mass) OR (mineral mass) OR (fat mass) OR (fat free mass) OR (lean body mass) OR (functional body composition) OR (flexibility) OR (range of motion) OR (muscle stretching exercises)) AND ((HIV infections) OR (HIV) OR (human immunodeficiency virus) OR (HIV seropositivity) OR (HIV testing) OR (acquired immunodeficiency syndrome) OR (AIDS)) AND ((child) OR (children) OR (adolescent) OR (adolescents) OR (youth) OR (young) OR (teen) OR (teenager) OR (preschool child) OR (boys) OR (girls) OR (pediatric))

Portuguese

((aptidão física) OR (aptidão física relacionada a saúde) OR (aptidão cardiorrespiratória) OR (consumo de oxigênio) OR (capacidade aeróbica) OR (aptidão aeróbica) OR (capacidade cardiorrespiratória) OR (aptidão cardiovascular) OR (potência aeróbica) OR (resistência aeróbica) OR (resistência cardiovascular) OR (consumo máximo de oxigênio) OR (absorção máxima de oxigênio) OR (VO2 máximo) OR (força muscular) OR (contração muscular) OR (contração isométrica) OR (contração isotônica) OR (treinamento resistido) OR (potência muscular) OR (treinamento resistido) OR (força de membros superiores) OR (força de membros inferiores) OR (resistência muscular) OR (aptidão musculoesquelética) OR (aptidão muscular) OR (força explosiva) OR (composição corporal) OR (análise da composição corporal) OR (tecido adiposo) OR (densidade óssea) OR (massa óssea) OR (massa mineral óssea) OR (gordura corporal) OR (massa livre de gordura) OR (massa magra) OR (composição corporal funcional) OR (flexibilidade) OR (amplitude de movimento) OR (exercícios de alongamento muscular)) AND ((infecção por HIV) OR (HIV) OR (vírus da imunodeficiência humana) OR (HIV soropositivo) OR (diagnóstico de HIV) OR (síndrome da imunodeficiência adquirida) OR (AIDS)) AND ((criança) OR (crianças) OR (adolescente) OR (adolescentes) OR (jovens) OR (jovem) OR (pré-escolares) OR (meninos) OR (meninas) OR (população pediátrica))

Spanish

((aptitud física relacionada con la salud) OR (aptitud física) OR (aptitud cardiorrespiratoria) OR (consumo de oxígeno) OR (capacidad aeróbica) OR (aptitud aeróbica) OR (capacidad cardiorrespiratoria) OR (capacidad cardiovascular) OR (potencia aeróbica) OR (resistencia aeróbica) OR (resistencia cardiovascular) OR (consumo máximo de oxígeno) OR (consumo máximo de oxígeno) OR (VO2 max) OR (fuerza muscular) OR (contracción muscular) OR (contracción isométrica) OR (contracción isotónica) OR (entrenamiento de resistencia) OR (fuerza muscular) OR (entrenamiento de resistencia) OR (fuerza de miembros superiores) OR (fuerza de miembros inferiores) OR (resistencia muscular) OR (aptitud musculo esquelética) OR (aptitud muscular) OR (fuerza explosiva) OR (composición corporal) OR (análisis de la composición corporal) OR (tejido

adiposo) OR (densidad ósea) OR (masa ósea) OR (masa mineral ósea) OR (grasa corporal) OR (masa sin grasa) OR (masa magra) OR (composición corporal funcional) OR (flexibilidad) OR (rango de movimiento) OR (ejercicios de estiramiento muscular)) AND ((infección por VIH) OR (VIH) OR (virus de la inmunodeficiencia adquirida) OR (VIH seropositivo) OR (diagnóstico de VIH) OR (síndrome de inmunodeficiencia adquirida) OR (SIDA)) AND ((niño) OR (niños) OR (adolescente) OR (adolescentes) OR (jóvenes) OR (preescolares) OR (niños) OR (niñas) OR (población pediátrica))

9 - Science Direct (https://www-sciencedirect.ez46.periodicos.capes.gov.br/search)

- Start: 05/01/2024 End: 05/01/2024
- Total: 1183

Filters: “Research articles”

("health related physical fitness” OR “cardiorespiratory fitness” OR "muscle strength" OR “body composition analysis” OR “Range of Motion", Articular”) AND (“HIV” OR “AIDS”) AND ("children" OR "adolescents")

10 - CINAHL
(https://web-s-ebscohost.ez46.periodicos.capes.gov.br/ehost/search/advanced?vid=24&sid=b165b3b0-01a4-4f7f-8c7d-91d98f0906a8%40redis)

- Start: 05/01/2024 End: 05/01/2024
- Total: 544

Filters: (*Expanders - Apply equivalent subjects; Search Modes - Boolean/Phrase)*

TX ((“physical fitness”) OR (“health-related physical fitness”) OR (“cardiorespiratory fitness”) OR (“oxygen consumption”) OR (“aerobic capacity”) OR (“aerobic fitness”) OR (“cardiorespiratory capacity”) OR (“cardiovascular fitness”) OR (“aerobic power”) OR (“aerobic endurance”) OR (“cardiorespiratory endurance”) OR (“maximum oxygen consumption”) OR (“maximal oxygen uptake”) OR (“VO2 maximal”) OR ("muscle strength") OR ("muscle contraction") OR ("isometric contraction") OR ("isotonic contraction") OR ("resistance training") OR ("muscular power") OR (“endurance training”) OR ("upper limb strength") OR ("lower limb strength") OR ("muscular endurance") OR ("musculoskeletal fitness") OR ("muscular fitness") OR (“explosive strength”) OR (“body composition”) OR (“body composition analysis”) OR (“adipose tissue”) OR (“bone density”) OR (“bone mass”) OR (“mineral mass”) OR (“fat mass”) OR (“fat free mass”) OR (“lean body mass”) OR (“functional body composition”) OR (“flexibility”) OR (“range of motion”) OR (“muscle stretching exercises”)) AND ((“HIV infections”) OR (“HIV”) OR (“human immunodeficiency virus”) OR (“HIV seropositivity”) OR (“HIV testing”) OR (“acquired immunodeficiency syndrome”) OR (“AIDS”)) AND ((“child”) OR (“children”) OR (“adolescent”) OR (“adolescents”) OR (“youth”) OR (“young”) OR (“teen”) OR (“teenager”) OR (“preschool child”) OR (“boys”) OR (“girls”) OR (“pediatric”))

Supplementary Table S1. Publication year, first author, country, design, purpose, and participants of the studies included in the scoping review.

Nº	Year	First Author	Country	Study design	Study purpose	Groups	Sample size	Sex	Age in years (SD)
1	1995	Miller	United States	Analytic Intervention	To determine whether enteral supplementation with gastrostomy tubes improved weight, height, lean body mass, fat mass, immunologic parameters, length of hospital stay, and survival of HIV-infected children	HIV+	23 HIV+	14 F<br>25 M	2.6 (0.38)
2	1995	Saavedra	United States	Analytic Case-control	To describe and to evaluate the longitudinal growth of children born to mothers with HIV	HIV+<br>HIV-	59 HIV+<br><br>50 HIV-	33 F<br>26 M<br>25 F<br>25 M	>2.0 (NR)
3	1996	Arpadi	United States	Descriptive Method Validity	To evaluate the ability of standard BIA equations to predict total body water and fat free mass in children with HIV	HIV+	20 HIV+	11 F<br>9 M	6.5 (2.3)
4	1997	Miller	United States	Descriptive Correlational	To analyze the relation of nutritional status to cardiac muscle mass and function in HIV-infected children	HIV+	36 HIV+	17 F<br>19 M	2.8 (NR)
5	1998	Arapadi	United States	Analytic Case-control	To characterize the body composition of children with HIV	HIV+<br>HIV-	34 HIV+<br><br>52 HIV-	17 F<br>17 M<br>30 F<br>22 M	6.8 (2.2)<br>6.8 (2.4)<br>7.9 (1.7)<br>8.4 (2.9)
6	1998	Henderson	United States	Analytic Case-control	To determine whether alterations in body composition, resting energy expenditure and dietary intake are associated with growth retardation in HIV-infected children	HIV+<br>HIV-	32 HIV+<br><br>10 HIV-	15 F<br>17 M<br>4 F<br>6 M	6.5 (1.7)<br>6.7 (2.3)<br>7.1 (2.8)
7	1999	Fontana	Italy	Analytic Case-control	To study FFM in a large group of HIV-infected children and its correlation with the different stages of illness and with survival	HIV+<br>HIV-	86 HIV+<br><br>113 HIV-	50 F<br>36 M<br>63 F<br>50 M	6.9 (3.1)<br><br>7.7 (3.3)
8	1999	Fox-Wheeler	United States	Analytic Open label Clinical trial	To determine the safety and efficacy of anabolic therapy to prevent or reverse wasting and malnutrition in HIV-infected pediatric patients	HIV+	10 HIV+	4 F<br>6 M	(4 to14)<br>(7 to12)
9	2000	Arpadi	United States	Descriptive Correlational	To assess the relationships among HIV replication, energy balance, body composition and growth in children with HIV-associated growth failure	HIV+	42 HIV+	24 F<br>18 M	8.3 (2.4)
10	2000	Fiore	Italy	Analytic Observational	To determine changes in nutritional status based on body weight, height and nutritional habits, of HIV-infected children receiving ART	HIV+	25 HIV+	11 F<br>14 M	7.7 (3.8)
11	2000	Heller	United States	Descriptive Method Validity	To produce a simple and effective instrument to evaluate and monitor the nutritional risk of children HIV infected	HIV+	39 HIV+	20 F<br>19 M	8 (NR)
12	2000	Jansen	Brazil	Analytic Case-control	To evaluate the nutritional status of children with HIV	HIV+<br>HIV-	36 HIV+<br><br>36 HIV-	14 F<br>22 M<br>NR	1.5-5.0<br><br>1.5-5.0
13	2000	Jaquet	France	Descriptive Correlational	To investigate body fat distribution and glucose and lipid metabolism in HIV infected children with	HIV+	39 HIV+	20 F<br>19 M	9.1 (4.0)
14	2000	Keyser	United States	Descriptive Correlational	To determine the degree to which cardiorespiratory insufficiency limited physical performance of adolescents who were seropositive for HIV	HIV+	17 HIV+	12 F<br>5 M	18 (2.0)
15	2000	Mismer	United States	Analytic Observational	To determine which nutritional, anthropometric, clinical, and social factors are predictive of a change in functional status	HIV+	35 HIV+	14 F<br>21 M	5.3 (2.5)
16	2001	Arpadi	United States	Analytic	To characterize the change in regional fat over time in a sample of	HIV+	28 HIV+	18 F	7.5 (2.3)

				Observational	HIV-infected children			10 M	
17	2001	Brambilla	Italy	Analytic<br>Case-control	To verify changes in fat distribution and of increased metabolic risk in HIV-infected children	HIV+<br>HIV-	34 HIV+	18 F<br>16 M	11.9 (3.0)
							34 HIV-	18 F<br>16 M	11.9 (2.9)
18	2001	Dreimane	United States	Descriptive<br>Pilot study	To study the effects of protease inhibitors on the height and weight of HIV-1-infected children	HIV+	27 HIV+	11 F<br>16 M	6.54 (3.3)
19	2001	Ellis	United States	Descriptive<br>Method Validity	To develop an anthropometry-based prediction model for the assessment of bone mineral content in children	HIV+<br>HIV-	14 HIV+	6 F<br>8 M	7.7 (2.2)<br>10.3 (1.9)
							982 HIV-	537 F<br>445 M	11.63 (3.8)<br>11.17 (3.73)
20	2001	Melvin	United States	Descriptive<br>Correlational	To determine blood lipid levels, insulin sensitivity, and body composition in HIV-1-infected children	HIV+	35 HIV+	23 F<br>12 M	9.0 (4–18)
21	2001	Miller	United States	Analytic<br>Observational	To determine the effect of protease inhibitors on growth and body composition in children with human immunodeficiency virus type 1 infection	HIV+	67 HIV+	34 F<br>33 M	6.8 (3.7)
22	2001	Mora	Italy	Analytic<br>Case-control	To evaluate the occurrence and define the aetiology of osteopenia in children receiving highly active antiretroviral therapy	HIV+<br>HIV-	40 HIV+	22 F<br>18 M	11.5 (1.97)
							314 HIV-	140 F<br>174 M	(4.9 to 18.5)
23	2001	O'Brien	United States	Descriptive<br>Correlational	To examine the effect of HIV infection on calcium status and bone growth in children	HIV+	19 HIV+	19 F	9.2 (2.6)
24	2001	Tan	United States	Analytic<br>Case-control	To measured serum osteocalcin levels in prepubertal children with human immunodeficiency virus	HIV+<br>HIV-	42 HIV+	29 F<br>13 M	7.0 (2.8)
							36 HIV-	15 F<br>11 M	6.8 (2.6)
25	2002	Amaya	United States	Descriptive<br>Correlational	To determine the prevalence of fat redistribution, hyperlipidemia and insulin resistance in HIV-infected children	HIV+	40 HIV+	22 F<br>18 M	9.1 (2.9)
26	2002	Arpadi	United States	Analytic<br>Case-control	To evaluate the effect of HIV infection on bone mineral content in children	HIV+<br>HIV-	51 HIV+	25 F<br>26 M	8.2 (2.6)
							262 HIV-	129 F<br>135 M	8.4 (1.6)
27	2002	Cade	United States	Analytic<br>Case-control	To determine if aerobic capacity was diminished in late adolescents infected with HIV compared to controls	HIV+<br>HIV-	15 HIV+	11 F<br>4 M	18.3 (0.03)
							15 HIV-	11 F<br>4 M	18.3 (0.03)
28	2002	Cossarizza	Italy	Analytic<br>Case-control	To verify the association between mitochondrial toxicity and the lipodystrophy syndrome in pediatric patients	HIV+<br>HIV-	18 HIV+	10 F<br>8 M	(4 to 16)
							6 HIV-	4 F<br>2 M	11.7 (6.4)
29	2002	Horlick	United States	Descriptive<br>Method Validity	To evaluate the performance of 13 published pediatric BIA-based predictive equations for total body water and fat-free mass and to refit the best performing model	HIV+<br>HIV-	54 HIV+	30 F<br>24 M	(4 to 15)
							1247 HIV-	597 F<br>640 M	(4 to 18)
30	2002	Nachman	United States<br>Puerto Rico	Analytic<br>Clinical trial	To determine if age- and sex-adjustment growth z-scores correlative with HIV-1 RNA level	HIV+	197 HIV+	106 F<br>91 M	7.2 (NR)
31	2002	Rondanelli	Italy	Analytic	To better characterize the somatotropic axis by measuring	HIV+	15 HIV+	7 F	9.1 (1.4)

				Case-control	spontaneous 24-hr secretion of GH and spontaneous and stimulated IGF-I and IGF-binding protein 3 (IGFBP-3) secretion in children with HIV infection	HIV-	16 HIV-	8 M<br>6 F<br>10 M	8.6 (1.3)
32	2002	Verweel	Netherlands	Descriptive<br>Correlational	To evaluate the effect of highly active antiretroviral therapy on growth in children with HIV-1 infection	HIV+	24 HIV+	13 F<br>11 M	5.2 (0.4-16.3)*
33	2003	Beregszaszi	France	Analytic<br>Case-control	To assess in situ the insulin sensitivity of the lipohypertrophic subcutaneous abdominal adipose tissue using the microdialysis technique in HIV-infected children	HIV+<br>HIV-	14 HIV+<br>6 HIV-	5 F<br>9 M<br>2 F<br>4 M	12.6 (4.2)<br>10.7 (1.5)
34	2003	Bitnun	Canada	Descriptive<br>Correlational	To determine the extent and degree of abnormalities of serum lipids, glucose homeostasis and abdominal adipose tissue distribution in PI-treated and PI-naive HIV-infected children	HIV+	50 HIV+	23F<br>27 M	8.6 (4.4)
35	2003	Bockhorst	United States	Descriptive<br>Correlational	To examine the relationships between lipodystrophy, PI use, lipid abnormalities and markers of insulin sensitivity children in the pediatric HIV	HIV+	26 HIV+	NR	9.7 (4.6-14.9)*
36	2003	McComsey	United States	Analytic<br>Open label<br>Clinical trial	To evaluate the virologic and immunologic effect of PI substitution with efavirenz in HIV-infected children	HIV+	17 HIV+	10 F<br>7 M	10.0 (NR)
37	2003	Vigano	Italy	Descriptive<br>Correlational	To characterize the growth hormone profile and the insulin-like growth factor system in treated HIV adolescents with and without excess visceral fat accumulation	HIV+	25 HIV+	14 F<br>11 M	13.8 (3.1)
38	2003	Vigano	Italy	Analytic<br>Case-control	To assess body composition changes in HIV-infected children receiving highly active antiretroviral therapy	HIV+<br>HIV-	37 HIV+<br>54 HIV-	20 F<br>17 M<br>30 F<br>24 M	12.2 (2.9)<br>11.8 (2.8)
39	2003	Zamboni	Italy	Descriptive<br>Correlational	To identify possible bone alterations in HIV-infected children	HIV+	13 HIV+	9 F<br>4 M	7.8 (2.9)
40	2004	Ghaffari	United States	Analytic<br>Clinical Trial	To evaluate 96-week clinical and immune outcomes to protease inhibitor– containing antiretroviral therapy	HIV+	40 HIV+	16 F<br>24 M	7.1 (NR)
41	2004	Hardin	United States	Analytic<br>Case-control	To measure protein turnover in HIV-infected children and to compare these values to those obtained from age and weight-matched healthy children	HIV+<br>HIV-	8 HIV+<br>8 HIV-	5 F<br>3 M<br>5 F<br>3 M	4.9 (1.7)<br>4.7 (1.4)
42	2004	Mora	Italy	Analytic<br>Case-control	To monitor the changes of BMD and bone metabolism over a period of 12 months	HIV+<br>HIV-	32 HIV+<br>381 HIV-	15 F<br>17 M<br>172 F<br>209 M	12.4 (0.5)<br>(5.7 to 19.2)
43	2004	Panamonta	Thailand	Descriptive<br>Correlational	To assess the endocrine function of Thai children infected with HIV	HIV+	36 HIV+	24 F<br>12 M	7.0 (2.0)
44	2004	Rojo	Spain	Descriptive<br>Correlational	To evaluate the prevalence of alterations in bone mineral density and possible associated factors in a cohort of HIV-infected children	HIV+	50 HIV+	25 F<br>25 M	10.6 (5.3)
45	2004	Stagi	Italy	Analytic<br>Case-control	To evaluate the bone quality and assess the role of the insulin growth factor system in the bone metabolism and skeletal status of HIV-1 perinatally infected children	HIV+<br>HIV-	44 HIV+<br>55 HIV-	26 F<br>18 M<br>23 F<br>22 M	8.4 (2.1)<br>8.4 (2.1)
46	2004	Taylor	United States	Analytic<br>Clinical Trial	To characterize the type and frequency of biochemical lipid abnormalities and physical changes in body composition associated	HIV+	94 HIV+	41 F<br>53 M	(0.5 to 17.9)

					with the use of PI-containing antiretroviral therapy among HIV – infected children treated for up to 6 years				
47	2004	Thorne	Italy<br>Spain<br>Belgium<br>Germany<br>Sweden<br>United Kington<br>Netherlands<br>Denmark<br>Poland<br>Switzerland	Descriptive<br>Correlational	To estimate prevalence of body fat redistribution and dyslipidemia in HIV-infected children	HIV+	477 HIV+	250 F<br>22 M	9.78 (3-18)*
48	2005	Aldámiz-Echevarría	Spain	Analytic<br>Case-control	To study plasma fatty acid composition in HIV-infected children	HIV+<br>HIV-	17 HIV+<br><br>112 HIV-	9 F<br>8 M<br>NR	6.7 (4.1)<br><br>NR
49	2005	Bitnun	Canada	Descriptive<br>Correlational	To define more precisely the impact of PI therapy on glucose homeostasis in HIV-infected children	HIV+	48 HIV+	21 F<br>27 M	9.1 (4.3)
50	2005	Giacomet	Italy	Analytic<br>Clinical Trial<br>Case-control	To assess whether the substitution of stavudine with tenofovir would result in decreased bone mineral content and bone mineral density accrual in HIV-infected children	HIV+<br>HIV-	16 HIV+<br>166 HIV-	NR	13.3 (6.4-17-9)*<br>13.1 (5.7-19.9)*
51	2005	Hardin	United States	Analytic<br>Clinical Trial	To explore the effect of growth hormone on protein catabolism in HIV-infected children	HIV+	6 HIV+	2 F<br>4 M	9.2 (NR)
52	2005	Hazra	United States	Analytic<br>Open label<br>Clinical trial	To provide preliminary pediatric safety and dosing information on tenofovir disoproxil fumarate	HIV+	18 HIV+	7 F<br>11 M	12.0 (2.5)
53	2005	Jacobson	United States	Analytic<br>Case-control	To compare bone mineral density among HIV-infected children with population norms	HIV+<br>HIV-	37 HIV+<br><br>9 HIV-	19 F<br>18 M<br>3 F<br>9 M	16.6 (9.6-13.8)*<br><br>10.4 (7.8-11.4)*
54	2005	Mora	Italy	Analytic<br>Case-control	To describe the bone mass measurements of a group of horizontally HIV-infected youth who were not receiving ART	HIV+<br>HIV-	16 HIV+<br><br>119 HIV-	10 F<br>6 M<br>63 F<br>56 M	9.33 (3.96)<br><br>9.74 (3.33)
55	2005	Pitukcheewanont	United States	Analytic<br>Case-control	To evaluate bone measurements in HIV-1 infected children and adolescents	HIV+<br>HIV-	58 HIV+<br><br>58 HIV-	32 F<br>26 M<br>32 F<br>26 M	12.03 (3.88)<br><br>12.1 (3.86)
56	2005	Rosso	Italy	Descriptive<br>Correlational	To examine the effects of disease and therapy-associated factors on bone mass	HIV+<br>HIV-	44 HIV+<br><br>1227 HIV-	23 F<br>21 M<br>568 F<br>641 M	10.8 (4.0)<br>10.0 (4.0)<br>(3 to 16)
57	2005	Vigano	Italy	Analytic<br>Open label<br>Clinical trial	To assess the efficacy of recombinant human growth hormone treatment on lipodystrophy in HIV-infected adolescents	HIV+<br>HIV-	8 HIV+<br><br>97 HIV-	5 F<br>3 M<br>46 F<br>51 M	15.7 (13.7-18.5)*<br><br>14.5 (3.0)

58	2006	Barros	Brazil	Descriptive<br>Correlational	To describe physical fitness variables of children and adolescents HIV-infected	HIV+	33 HIV+	11 F<br>22 M	8.9 (1.9)<br>8.6 (1.6)
59	2006	Ergun-Longmire	United States	Descriptive<br>Correlational	To evaluate the effects of PI as ART in comparison with other ART non-PI medications on glucose tolerance, lipid metabolism, and body fat distribution in HIV-infected young patients	HIV+	21 HIV+	15 F<br>6 M	11.9 (NR)
60	2006	Gafni	United States	Analytic<br>Open label<br>Clinical trial	To test the association of tenofovir disoproxil fumarate with normal skeletal growth	HIV+	15 HIV+	5 F<br>10 M	12.0 (2.0)
61	2006	Gutiérrez	Uruguay	Descriptive<br>Correlational	To describe the lipodystrophy prevalence in HIV-infected children	HIV+	60 HIV+	NR	6.8 (3.3)
62	2006	Haroun	United Kingdom	Analytic<br>Case-control	To assess growth and obesity status in outpatients characterized by diseases traditionally associated with poor growth or under-nutrition	HIV+<br>HIV-	49 HIV+<br><br>57 HIV-	24 F<br>25 M<br>25 F<br>32 M	10.0 (4.3)<br><br>10.8 (4.0)
63	2006	Hartman	Netherlands	Descriptive<br>Correlational	To obtain an objective case definition of the lipodystrophy syndrome	HIV+	32 HIV+	14 F<br>18 M	(0.7 to 17.1)
64	2006	Moscocki	United States	Analytic<br>Case-control	To investigate whether factors influencing body composition may be unique for male and female adolescents with horizontal transmission of HIV	HIV+<br>HIV-	326 HIV+<br><br>193 HIV-	236 F<br>90 M<br>146 F<br>47 M	16.8 (1.1)<br>17.2 (0.9)<br>16.5 (1.3)<br>16.8 (1.2)
65	2006	Verkauskiene	France	Descriptive<br>Correlational	To investigate fat redistribution and metabolic abnormalities in HIV-infected children	HIV+	130 HIV+	66 F<br>64 M	10.0 (2.0-18.0)*
66	2006	Weidle	Romania<br>Uganda<br>Botswana<br>Cote d'Ivoire<br>Kenya<br>Mozambique<br>Rwanda<br>South Africa<br>Zambia	Analytic<br>Clinical Trial	To compares the precision of weight-based doses for zidovudine and didanosine as compared with body surface area-based doses using height, weight and age information in HIV-infected children	HIV+	826 HV+	395 F<br>429 M	9.6 (NR)
67	2007	Chantry	United States<br>Puerto Rico	Analytic<br>Case-control	To investigated endocrine differences between perinatally HIV-infected and HIV-exposed, uninfected control children	HIV+<br>HIV-	21 HIV+<br><br>46 HIV-	9 F<br>12 M<br>26 F<br>20 M	4.58 (NR)<br><br>4.53(NR)
68	2007	Dzwonek	United Kingdom	Descriptive<br>Correlational	To determine whether there is an association of leptin with lipodystrophy in HIV-infected children	HIV+	104 HIV+	53 F<br>51 M	9.5 (3.6)
69	2007	Ene	Belgium	Descriptive<br>Correlational	To assess the prevalence of the lipodystrophy syndrome in our cohort of HIV-1 infected children	HIV+	88 HIV+	52 F<br>36 M	11.1 (NR)
70	2007	Kim	United States	Descriptive<br>Correlational	To compare growth, lipids and adipocytokines in HIV-positive children with and without lipoatrophy	HIV+	33 HIV+	15 F<br>18 M	11.8 (6.5-18.6)*<br>12.1 (6.6-20.6)*
71	2007	McComsey	United States	Analytic<br>Case-control	To assess carotid intima media thickness and cardiac biomarkers in HIV infected children	HIV+<br>HIV-	31 HIV+<br><br>31 HIV-	20 F<br>11 M<br>21 F<br>10 M	9.0 (2-20)<br><br>9.0 (2-21)
72	2007	Mora	Italy	Analytic<br>Case-control	To quantify the serum concentrations of osteoprotegerin and nuclear factor kappa B ligand in a cohort of HIV-infected children	HIV+<br>HIV-	27 HIV+	14 F<br>13 M	12.6 (0.7)

							336 HIV-	162 F<br>173 M	(4.8 to 17.9)
73	2007	Papaevangelou	Greece	Analytic<br>Case-control	To study leptin levels in serial serum samples of HIV-infected children before and after ART	HIV+<br>HIV-	8 HIV+	6 F<br>2 M	3.5 (NR)
							3 HIV-	3 F	7.0 (NR)
74	2007	Tremechin	Brazil	Analytic<br>Case-control	To compare the nutritional status and the 24-hour urine excretion of N^1-methylnicotinamideamong HIV-positive and HIV-negative children	HIV+<br>HIV-	20 HIV+	8 F<br>12 M	7.85 (1.7)
							20 HIV-	8 F<br>12 M	8.35 (1.5)
75	2007	Vigano	Italy	Analytic<br>Open label<br>Clinical trial	To assess the effect on body composition parameters of replacing stavudine with tenofovir and PI efavirenz in pediatric patients	HIV+<br>HIV-	24 HIV+	12 F<br>12 M	12.4 (3.9)
							143 HIV-	NR	(4.9 to 20.0)
76	2007	Vigano	Italy	Analytic<br>Open label<br>Clinical trial	To assess renal safety and glomerular filtration rate changes as estimated by the Schwartz and Cockcroft-Gault equations in HIV infected children treated with tenofovir for 96 weeks	HIV+<br>HIV-	27 HIV+	14 F<br>13 M	12.4 (3.9)
							143 HIV-	67 F<br>76 M	12.3 (4.4)
77	2008	Chantry	United States	Analytic<br>Case-control	To describe insulin-like growth factor-1 and insulin-like growth factor-1–binding protein-1 and insulin-like growth factor-3–binding protein-3 in HIV+ children before and after initiating or changing ART	HIV+<br>HIV-	97 HIV+	52 F<br>45 M	5.9 (3.6)
							NR	NR	NR
78	2008	Gonzales-Tome	Spain	Analytic<br>Experimental	To describe the effects in metabolic abnormalities in seven HIV-infected children, previously treated with PI after switching to nevirapine	HIV+	7 HIV+	2 F<br>5 M	11.1 (8.3)
79	2008	Miller	United States	Analytic<br>Case-control	To determine risk factors for cardiovascular disease in children infected with HIV	HIV+<br>HIV-	42 HIV+	27 F<br>15 M	10.1 (NR)
							4437 HIV-	2219 F<br>2218 M	8.8 (NR)
80	2008	Purdy	United States	Analytic<br>Open label<br>Clinical trial	To characterize the change in BMD during and after treatment with tenofovir disoproxil fumarate in a separate cohort of children and adolescents infected with HIV	HIV+	6 HIV+	2 F<br>4 M	12.8 (NR)
81	2008	Sharma	United States	Analytic<br>Observational	To analyzed dietary macronutrient intake in HIV-infected children	HIV+	116 HIV+	67 F<br>49 M	6.6 (3.1-18.5)*<br>8.5 (3.1-19.0)*
82	2008	Spagnoulo	Italy	Analytic<br>Case-control	To determine whether or not serum levels of resistin are marker of fat redistribution in HIV-infected children	HIV+<br>HIV-	18 HIV+<br>14 HIV-	NR	10.3 (3.2)<br>10.7 (3.4)
83	2009	Aldrovandi	United States<br>Puerto Rico	Analytic<br>Case-control	To compare the distribution of lipid and glucose abnormalities and altered fat distribution among vertically HIV-infected subjects and controls	HIV+<br>HIV-	240 HIV+	113 F<br>127 M	12.6 (NR)
							146 HIV-	62 F<br>84 M	11.9 (NR)
84	2009	Arpadi	United states	Analytic<br>Case-control	To compare changes in regional fat distribution in HIV-infected and healthy children and adolescents	HIV+<br>HIV-	64 HIV+	33 F<br>31 M	10.3 (3.7)
							147 HIV-	74 F<br>73 M	11.6 (2.8)
85	2009	Lopez	Colombia	Analytic<br>Case-control	To describe metabolic alterations in HIV-infected children	HIV+<br>HIV-	35 HIV+<br>35 HIV-	NR<br>NR	8.37 (NR)<br>8.31 (NR)
86	2009	Mora	Italy	Descriptive<br>Method Validity	To assess applicability of quantitative ultrasonography for bone health assessment in HIV-infected youths	HIV+	88 HIV+	45 F<br>43 M	(4.8 to 22.1)
87	2009	Sarni	Brazil	Descriptive<br>Correlational	To evaluate the presence of clinical lipodystrophy in HIV-infected children	HIV+	30 HIV+	16 F<br>14 M	9.1 (2.5)

88	2009	Vigano	Italy	Analytic Observational	To describe a 4-year course of glucose homeostasis in a cohort of HIV-infected children and adolescents	HIV+	37 HIV+	20 F<br>17 M	12 (7-12)*
89	2010	Cervia	United States	Analytic Observational	To address the dearth of knowledge regarding associations of pro-inflammatory cytokines with measures of disease progression, growth, body composition and metabolism in HIV-infected children	HIV+	49 HIV+	28 F<br>21 M	7.0 (3.7)
90	2010	Chantry	United States	Analytic Observational	To describe growth and body composition changes in HIV-positive children	HIV+	97 HIV+	52 F<br>45 M	5.9 (3.6)
91	2010	Jacobson	United States	Analytic Case-control	To characterize total body BMC and total body and spinal BMD in perinatally HIV-infected and uninfected children	HIV+<br>HIV-	236 HIV+	112 F<br>124 M	12.6 (NR)
							143 HIV-	60 F<br>83 M	11.9 (NR)
92	2010	Miller	United States	Analytic Case-control	To compared biomarkers of vascular dysfunction among HIV-infected children to a demographically similar group of uninfected children	HIV+<br>HIV-	106 HIV+	59 F<br>47 M	14.8 (4.3)
							55 HIV-	24 F<br>26 M	12.3 (3.8)
93	2010	Miller	United States	Analytic Experimental	To determine if a structured training program in HIV-infected children is feasible and safe, improves fitness and strength, and changes body composition	HIV+	17 HIV+	8 F<br>9 M	15.0 (6.0-22.6)*
94	2010	Stagi	Italy	Analytic Observational	To present the auxological data up to final height in a cohort of patients with perinatal HIV infection	HIV+	95 HIV+	57 F<br>38 M	17.5 (13.7-23.2)*
95	2010	Vigano	Italy	Analytic Experimental	To assess the safety of a tenofovir disoproxil fumarare-containing ART on BMD in pediatric patients	HIV+	21 HIV+	11 F<br>10 M	12.1 (4.9-17.9)*
96	2010	Werner	Brazil	Descriptive Correlational	To describe lipid profile, body shape changes, and cardiovascular risk factors in HIV- infected children and adolescents	HIV+	43 HIV+	21 F<br>22 M	9.6 (1.9)
97	2010	Zuccotti	Italy	Analytic Case-control	To assess the role of different antiretroviral treatments on skeletal health in a cohort of HIV-infected children and adolescents	HIV+<br>HIV-	86 HIV+	47 F<br>36 M	14.3 (0.8)<br>15.1 (0.8)
							194 HIV-	90 F<br>104 M	13.0 (0.5)<br>13.0 (0.4)
98	2011	Contri	Brazil	Analytic Observational	To describe nutritional status, body composition and lipid profile in children and adolescents receiving protease inhibitors	HIV+	59 HIV+	30 F<br>29 M	9.5 (4.5-16.3)*<br>9.54 (5.0-15.8)*
99	2011	da Silva	Brazil	Descriptive Correlational	To verify the presence of body and metabolic alterations as well as body satisfaction in children and teenagers undergoing ART	HIV+	38 HIV+	22 F<br>16 M	9.9 (3.0)
100	2011	Dimock	United States	Analytic Observational	To characterize metabolic disturbances both cross-sectionally and over time in a group of HIV-infected adolescents and young adults	HIV+	39 HIV+	19 F<br>20 M	17.5 (3.7)
101	2011	Geffner	United States<br>Puerto Rico	Descriptive] Correlational	To determine the prevalence of insulin resistance in HIV-infect children and adolescents	HIV+	402 HIV+	214 F<br>188 M	12.4 (2.3)
102	2011	Jacobson	United States	Analytic Case-control	To compare total body fat and its distribution in perinatally HIV-infected and HIV-exposed uninfected	HIV+<br>HIV-	369 HIV+	196 F<br>173 M	12.2 (2.6)
							89 HIV-	87 F<br>89 M	10.9 (2.3)
103	2011	Mohd	Malaysia	Descriptive Correlational	To determine the nutritional status of children living with HIV currently receiving ART	HIV+	95 HIV+	41 F<br>54 M	8.4 (3.9)
104	2011	Morén	Spain	Analytic Case-control	To determine the mitochondrial status of a group of HIV- infected children	HIV+<br>HIV-	69 HIV+	35 F<br>34 M	12.4 (1.2)
							24 HIV-	8 F<br>16 M	10.9 (1.2)
105	2011	Ramalho	Brazil	Analytic	To compare growth, nutritional status and body composition outcomes	HIV+	94 HIV+	39 F	12.72 (7.7-19.8)*

				Case-control	between HIV-infected children and adolescents on ART and healthy controls	HIV-	 364 HIV-	55 M<br>186 F<br>178 M	 12.1 (8.1-18.3)*
106	2011	Resino	Spain	Analytic<br>Observational	To evaluate the evolution of plasma adipokines and lipodystrophy in protease inhibitor-naive vertically HIV-infected children	HIV+	27 HIV+	19 F<br>8 M	9.4 (4.5-12.6)*
107	2011	Spoulou	Greece	Analytic<br>Case-control	To evaluate body composition by DXA in a cohort of Greek HIV-1 infected children and adolescents on ART	HIV+<br>HIV-	17 HIV+<br><br>159 HIV-	10 F<br>7 M<br>79 F<br>84 M	12.5 (4.0)<br><br>12.7 (4.9)
108	2011	Tremeschin	Brazil	Analytic<br>Case-control	To report longitudinal clinical data for pediatric patients continuously exposed to ART regimens and healthy controls groups	HIV+<br>HIV-	17 HIV+<br>16 HIV-	NR	10.5 (5.0-16.2)*
109	2011	Vigano	Italy	Analytic<br>Case-control	To investigated serum adiponectin concentration in a cohort of HIV-infected youths	HIV+<br>HIV-	36 HIV+<br><br>171 HIV-	20 F<br>16 M<br>91 F<br>80 M	13.3 (3.8)<br><br>(4.9 to 17.9)
110	2012	Alam	Belgium<br>Italy<br>Poland	Descriptive<br>Correlational	To estimate the prevalence of, and identify risk factors for Lipodystrophy Syndrome and body fat abnormality in a population of HIV-infected children and<br>adolescents	HIV+	426 HIV+	214 F<br>201 M	12.2 (NR)
111	2012	Arpadi	United States	Analytic<br>Clinical trial	To evaluate the effect of vitamin D and calcium supplementation on bone mass accrual in HIV-infected youth	HIV+	59 HIV+	33 F<br>26 M	10.2 (NR)
112	2012	Bhargav	India	Descriptive<br>Correlational	To determine the incidence of maternal to child transmission of HIV infection in the Belgaum district of Karnataka State, India	HIV+	44 HIV+	14 F<br>30 M	10.8 (3.1)
113	2012	Innes	South Africa	Descriptive<br>Correlational	To explore the prevalence and risk factors for lipoatrophy in a group of pre-pubertal South African children on ART	HIV+	100 HIV+	48 F<br>52 M	7.4 (5.9-9.3)*<br>5.9 (4.2-7.7)*
114	2012	Lindsey	United States<br>Puerto Rico	Analytic<br>Case-control	To investigate relationships between body composition, lipid levels and glucose metabolism in HIV-infected and HIV-uninfected children and young adults using data from a cross-sectional study	HIV+<br>HIV-	236 HIV+<br>143 HIV-	171 F<br>208 M	12.4 (7-24)*
115	2012	Miller	United States	Analytic<br>Case-control	To compare levels of biomarkers of vascular dysfunction in HIV-infected children (with and without hyperlipidemia) with those in HIV-exposed, uninfected children enrolled in the Pediatric HIV/AIDS Cohort Study	HIV+<br>HIV-	226 HIV+<br><br>140 HIV-	127 F<br>99 M<br>65 F<br>75 M	12.3 (10.4-14.0)*<br><br>10.1 (8.2-12.3)*
116	2012	Negra	United States<br>Brazil<br>Panama	Analytic<br>Clinical trial	To present the 48-week data on the efficacy and safety of tenofovir disoproxil fumarate in combination with an optimized background regimen in treatment-experienced HIV-1 infected adolescents with viremia despite ART	HIV+	87 HIV+	NR	(12 to 18)
117	2012	Puthanakit	Thailand	Analytic<br>Case-control	To assess the prevalence and predictors of low BMD among HIV-infected Thai adolescents receiving ART	HIV+<br>HIV-	101 HIV+<br><br>199 HIV-	49 F<br>51 M<br>NR	14.3 (1.3-15.7)*<br><br>NR
118	2012	Ramos	Puerto Rico	Analytic<br>Case-control	To determine the anaerobic power and muscle strength of preadolescents with human immunodeficiency virus	HIV+<br>HIV-	15 HIV+<br><br>15 HIV-	8 F<br>7 M<br>8 F<br>7 M	11 (7-14)*<br><br>11 (7-14)*
119	2012	Schtscherbyna	Brazil	Descriptive<br>Correlational	To assess the prevalence and factors associated with low BMD in HIV-infected adolescents	HIV+	74 HIV+	41 F<br>33 M	17.3 (1.8)
120	2013	Arpadi	South Africa	Descriptive<br>Correlational	To examine the lipid profiles, insulin sensitivity, markers of inflammation, and regional fat distribution of HIV-infected children in	HIV+	156 HIV+	75 F<br>81 M	5.1 (0.8)

					South Africa who were initiated on a protease inhibitors-based regimen prior to age 2 years				
121	2013	Bunders	Netherlands	Analytic Observational	To present longitudinal data on BMD obtained by DXA in combination antiretroviral therapy treated HIV-infected participants of the Pediatric Amsterdam HIV Cohort	HIV+	66 HIV+	36 F<br>30 M	6.7 (4.5-10.3)*
122	2013	Chokephaibulkit	Thailand	Descriptive Correlational	To assess the prevalence of vitamin D deficiency among 101 perinatally HIV-infected Thai adolescents receiving ART	HIV+	101 HIV+	50 F<br>51 M	14.3 (13.0-15.7)*
123	2013	DiMeglio	United States<br>Puerto Rico	Analytic Case-control	To estimate prevalence of low bone mineral density in perinatally HIV-infected and HIV-exposed but uninfected children, and to determine predictors of BMD	HIV+<br>HIV-	350 HIV+<br><br>160 HIV-	189 F<br>161 M<br>84 F<br>76 M	12.6 (10.2-14.4)*<br><br>10.7 (8.9-12.6)*
124	2013	dos Santos	Brazil	Descriptive Correlational	To identify the immunological and virological characteristics and flexibility and abdominal resistance strength variables of HIV-infected children and adolescents using ART	HIV+	63 HIV+	37 F<br>26 M	(7 to 17)
125	2013	Fabiano	Italy	Analytic Experimental	To describe the long-term (8-year) changes in growth, fat content and distribution, bone mass and metabolic parameters occurring in a series of 24 HIV-infected children who were switched from lamivudine + stavudine to tenofovir and from PI to efavirenz	HIV+	24HIV+	NR	12 (NR)
126	2013	Innes	South Africa	Descriptive Method Validity	To develop an anthropometric screening tool to detect lipoatrophy in prepubertal HIV- infected children	HIV+	100 HIV+	48 F<br>52 M	7.4 (5.9-9.3)*
127	2013	Lima	Brazil	Descriptive Correlational	To describe BMD and BMC in children and adolescents infected with the human immunodeficiency virus	HIV+	48 HIV+	24 F<br>24 M	12.5 (3.1)<br>12.9 (2.4)
128	2013	Macdonald	Canada	Analytic Observational	To determine if bone health is compromised in perinatally HIV-infected youth	HIV+	31 HIV+	12 F<br>19 M	13.6 (11.6-16.0)*
129	2013	Palchetti	Brazil	Descriptive Correlational	To identify lipodystrophy in prepubertal HIV-infected children using anthropometric parameters and body composition assessment	HIV+	40 HIV+	20 F<br>20 M	9.8 (1.2)
130	2013	Palchetti	Brazil	Descriptive Method Validity	To compare bioelectrical impedance analysis equations developed for healthy pediatric populations and for HIV-infected children using dual-energy X-ray absorptiometry as the gold standard	HIV+	40 HIV+	20 F<br>20 M	9.8 (1.2)
131	2013	Sharma	United States	Descriptive Correlational	To evaluate differences in mitochondrial DNA copy number and mitochondrial oxidative phosphorylation NADH dehydrogenase [Complex 1 (C1)] and cytochrome c oxidase [Complex 4 (C4)] enzyme activities among HIV-infected children with and without insulin resistance	HIV+	42 HIV+	22 F<br>20 M	13.45 (8.5-16.0)*<br>13.54 (8.7-16.2)*
132	2013	Somarriba	United States	Analytic Case-control	To compare VO2 peak, maximal strength and endurance, and flexibility of HIV-infected and uninfected children to determine if clinical and HIV-specific factors are associated with physical fitness	HIV+<br>HIV-	45 HIV+<br><br>36 HIV-	21 F<br>24 M<br>14 F<br>22 M	16.1 (2.7)<br><br>13.5 (3.0)
133	2014	Agustinho	Argentina	Analytic Case-control	To investigate the prevalence of risk factors for early cardiovascular disease in HIV-infected children and adolescents	HIV+<br>HIV-	77 HIV+<br><br>60 HIV-	27 F<br>50 M<br>25 F<br>35 M	12.2 (3.0-18.3)*<br><br>9.9 (4.8-16.9)*
134	2014	Dejkhamron	Thailand	Analytic Observational	To determine the prevalence of insulin resistance, dyslipidemia, and their inter-relationships in HIV-infected Thai children	HIV+	28 HIV+	10 F<br>18 M	5.5 (2.2-7.4)*
135	2014	Foissac	France	Analytic Observational	To investigate the population pharmacokinetics of 25-hydroxycholecalciferol D in HIV-1-infected children and adolescents	HIV+	91 HIV+	44 F<br>47 M	15.0 (11-17)*<br>14.0 (10-17)*
136	2014	Hillesheim	Brazil	Descriptive	To investigate the nutritional status and dietary intake of HIV-infected	HIV+	49 HIV+	24 F	12.4 (3.0)

				Correlational	children			25 M	12.8 (2.3)
137	2014	Humphries	South Africa	Descriptive<br>Correlational	To compare the muscle strength of children infected with HIV who had been receiving ART to that of children infected with HIV not receiving ART	HIV+	32 HIV+	NR	6.0 (1.2)
138	2014	Mussime	Uganda<br>Zambia	Analytic<br>Case-control	To compare anthropometric measurements and lipid profile in HIV-infected children and HIV-uninfected children	HIV+<br>HIV-	408 HIV+<br><br>88 HIV-	205 F<br>203 M<br>49 F<br>39 M	2.5 (1.5-4.0)*<br><br>2.2 (1.5-3.0)*
139	2014	Theeodoridou	Greece	Analytic<br>Case-control	To investigate the non-traditional adipokines Retinol-binding-Protein-4, neutrophil-gelatinase-associated-lipocalin, a-Fatty-Acid-Binding-Protein and YKL-40 in HIV-infected children on ART	HIV+<br>HIV-	17 HIV+<br><br>20 HIV-	10 F<br>7 M<br>NR	12.5 (3.98)<br>(6.0 to 15.0)
140	2014	Vreeman	Kenya	Analytic<br>Observational	To assess prospective nevirapine pharmacokinetics parameters in HIV-infected Kenyan children and to use mixed-effects modeling to assess sources of variation in prospective nevirapine pharmacokinetics parameters and drug exposure, focusing on body composition and adherence to ART	HIV+	21 HIV+	12 F<br>9 M	4.4 (NR)
141	2015	Aurpibul	Thailand	Analytic<br>Open label<br>Clinical trial	To evaluate the efficacy, safety and pharmacokinetics of tenofovir disoproxil fumarate in treatment-experienced children during 96 weeks	HIV+	80 HIV+	45 F<br>35 M	11.5 (3.5)
142	2015	Cohen	Netherlands<br>South Africa	Analytic<br>Case-control	To assess changes over time in regional fat mass in combination ART-treated, HIV-infected children on two continents	HIV+<br>HIV-	175 HIV+<br><br>43 HIV-	85 F<br>90 M<br>20 F<br>23 M	7.4 (5.1-10.2)*<br><br>5.2 (5.0-5.7)*
143	2015	Della Negra	Brazil<br>Panama	Analytic<br>Open label<br>Clinical trial	To present final results from the open-label tenofovir disoproxil fumarate extension following the randomized, placebo-controlled, double-blind phase of GS-US-104-0321 (Study 321)	HIV+	81 HIV+	46 F<br>35 M	14.0 (13.0-16.0)*
144	2015	dos Reis	Brazil	Descriptive<br>Correlational	To investigate the relationship between anthropometric parameters and body composition of perinatally HIV-infected children and adolescents under ART, according to use and non-use of PI	HIV+	115 HIV+	60 F<br>55 M	11.8 (2.9)
145	2015	Mora	Italy	Analytic<br>Case-control	To investigate the relationship between measurements of sclerostin and Dickkopf1 with bone formation and metabolism in HIV-infected and control subjects	HIV+<br>HIV-	54 HIV+<br><br>105 HIV-	35 F<br>19 M<br>57 F<br>48 M	13.6 (5.6-19.4)*<br><br>11.5 (4.5-17.7)*
146	2015	Palchetti	Brazil	Analytic<br>Observational	To evaluate bone mass accrual and determine the influence of clinical, anthropometric, dietary, and biochemical parameters on bone mass	HIV+	35 HIV+	18 F<br>17 M	9.6 (1.1)
147	2015	Swetha	India	Analytic<br>Observational	To assess the growth and morbidity status of HIV infected children over a period of one year in a city in southern India	HIV+	77 HIV+	40 F<br>37 M	9.3 (0.5)<br>9.0 (0.6)
148	2016	Arpadi	South Africa	Analytic<br>Case-control	To compare bone mass of South African HIV-infected children initiated on ART early in life with an HIV-uninfected control group	HIV+<br>HIV-	219 HIV+<br><br>219 HIV-	112 F<br>107 M<br>99 F<br>120 M	6.4 (1.2)<br><br>7.0 (1.5)
149	2016	Gaur	South Africa<br>Uganda<br>Thailand<br>United States	Analytic<br>Open label<br>Clinical trial	To assess safety, pharmacokinetics, and efficacy of this single-tablet, fixed-dose combination of elvitegravir, cobicistat, emtricitabine, and tenofovir alafenamide in HIV-infected, treatment-naive adolescents	HIV+	50 HIV+	28 F<br>22 M	15 (12-17)*
150	2016	Lima	Brazil	Descriptive<br>Method Validity	To develop predictive equations for bone mineral content and bone mineral density in children and adolescents living with HIV based on	HIV+	48 HIV+	24 F<br>24 M	12.7 (9.4-15.0)*<br>13.2 (11.1-14.7)*

					anthropometric variables				
151	2016	Sonego	El Salvador	Descriptive Correlational	To estimate the prevalence of dyslipidemia in children on ART in El Salvador	HIV+	173 HIV+	80 F<br>93 M	10. (3.0)
152	2016	Sudjaritruk	Thailand<br>Indonesia	Descriptive Correlational	To determine the prevalence of hypovitaminosis D and hyperparathyroidism and their effects on bone turnover and BMD among HIV-infected adolescents in Southeast Asia	HIV+	394 HIV+	225 F<br>169 M	15.0 (13.3-16.9)*
153	2016	Wong	South Africa	Analytic Case-control	To describe physical activity in South African children with and without HIV	HIV+<br>HIV-	218 HIV+<br><br>180 HIV-	110 F<br>108 M<br>81 F<br>99 M	6.0 (5.4-7.0)*<br><br>7.1 (5.7-8.6)*
154	2017	Carmo	Brazil	Analytic Observational	To evaluate the prevalence of BMD alterations and vitamin D concentrations in HIV-infected children and adolescents	HIV+	58 HIV+	35 F<br>23 M	14.2 (5.8-18.3)*
155	2017	de Lima	Brazil	Analytic Case-control	To examine aerobic fitness, total moderate to vigorous physical activity and also patterns in terms of moderate to vigorous physical activity between children and adolescents with HIV and controls	HIV+<br>HIV-	65 HIV+<br><br>65 HIV-	35 F<br>30 M<br>35 F<br>30 M	12.2 (2.1)<br><br>12.1 (1.8)
156	2017	Giacomet	Italy	Analytic Case-control	To assess the long−term effect of tenofovir disoproxil fumarate on bone mineral density in young patients	HIV+<br>HIV-	26 HIV+<br><br>202 HIV-	13 F<br>13 M<br>100 F<br>102 M	12.1 (5.0- 17.3)*<br><br>12.5 (5.2)
157	2017	Jacobson	United States	Analytic Case-control	To evaluate associations of low 25 hydroxy vitamin D and high parathyroid hormone concentrations with total body and spine BMD and BMC in HIV-infected children	HIV+<br>HIV-	412 HIV+<br><br>207 HIV-	217 F<br>195 M<br>101 F<br>106 M	13.0 (10.6-14.7)*<br><br>10.8 (9.0-12.8)*
158	2017	Jimenéz	Spain	Descriptive Correlational	To determine the prevalence and risk factors associated with low BMD in vertically HIV-infected patients	HIV+	98 HIV+	69 F<br>29 M	15.9 (12.9-17.0)*
159	2017	de Lima	Brazil	Descriptive Method Validity	To assess the validity of traditional anthropometric equations and to develop predictive equations of total body and trunk fat for children and adolescents living with HIV based on anthropometric measurements	HIV+	48 HIV+	24 F<br>24 M	12.4 (9.4-15.0)*<br>13.2 (11.1-14.7)*
160	2017	de Lima	Brazil	Analytic Experimental	To verify the effect of a playful exercise program on cardiovascular, morphological, metabolic, fitness, and quality of life outcomes	HIV+	10 HIV+	9 F<br>1 M	13.0 (11.5-15.5)*
161	2017	MacDonald	Canada	Analytic Case-control	To compare muscle power between youth who acquired HIV perinatally and HIV unexposed uninfected youth	HIV+<br>HIV-	35 HIV+<br><br>716 HIV-	17 F<br>20 M<br>339 F<br>377 M	13.9 (8.5-21.3)*<br><br>(9 to 21)
162	2017	Martins	Brazil	Analytic Case-control	To compare regular physical activity among adolescents living with HIV with their healthy peers, and to evaluate the relationship with anthropometric indicators of body fat	HIV+<br>HIV-	57 HIV+<br><br>54 HIV-	33 F<br>24 M<br>28 F<br>26 M	13.0 (1.5)<br><br>12.8 (2.3)
163	2017	Risti	Indonesia	Descriptive Correlational	To study the level vitamin D and calcium serum with mandibular bone density in HIV/AIDS children	HIV+	40 HIV+	NR	(6 to 15)
164	2017	Sudjaritruk	Thailand<br>Indonesia	Descriptive Correlational	To determine the prevalence of low bone mass and assess its relationship with abnormal bone turnover among HIV-infected Asian adolescents	HIV+	396 HIV+	226 F<br>170 M	15.0 (13.3-16.9)*
165	2017	Sudjaritruk	Thailand<br>Indonesia	Descriptive Correlational	To determine the prevalence of hypovitaminosis D and hyperparathyroidism and their effects on bone turnover and BMD	HIV+	394 HIV+	225 F<br>169 M	16.1 (14.7-17.4)*

					among HIV-infected adolescents in Southeast Asia				
166	2017	Ziegler	United States	Analytic<br>Case-control	To evaluate amino acid concentrations in HIV-infected children and young adults	HIV+<br>HIV-	79 HIV+<br><br>40 HIV-	41 F<br>38 M<br>19 F<br>21 M	9.0 (8.0-24.0)*
167	2018	Archary	South Africa	Analytic<br>Clinical Trial	To describe the effects of nutritional rehabilitation on Efavirenz and lopinavir pharmacokinetics in severely malnourished HIV-infected children and to explore the relationship between Efavirenz and lopinavir pharmacokinetic exposure and virologic outcomes	HIV+	63 HIV+	27 F<br>36 M	15.5 (16.3)
168	2018	Cames	Senegal	Descriptive<br>Correlational	To assess and identify risk factors affecting the prevalence of lipodystrophy in Senegalese children and adolescents on long-term antiretroviral treatment participating in a cohort study.	HIV+	254 HIV+	111 F<br>143 M	10.9 (8.1-14.2)*
169	2018	de Castro	Brazil	Descriptive<br>Method Validity	To determine the validity of body composition analysis by BIA compared to DXA and air displacement plethysmography in children and adolescents with HIV diagnosis	HIV+	64 HIV+	35 F<br>29 M	12.2 (2.1)<br>12.3 (2.2)
170	2018	de Lima	Brazil	Analytic<br>Case-control	To compare body image and anthropometric indicators among adolescents living with HIV and healthy adolescents	HIV+<br>HIV-	57 HIV+<br><br>54 HIV-	32 F<br>25 M<br>28 F<br>26 M	13.0 (1.5)<br><br>12.8 (2.3)
171	2018	de Lima	Brazil	Analytic<br>Case-control	To compare cardiovascular risk factors, chronic inflammation, and carotid intima-media thickness between the HIV and control groups	HIV+<br>HIV-	65 HIV+<br><br>65 HIV-	35 F<br>30 M<br>35 F<br>30 M	12.2 (2.1)<br><br>12.1 (1.8)
172	2018	Innes	South Africa	Analytic<br>Experimental	To explore intracellular stavudine triphosphate levels in children receiving a reduced dose of 0.5 to 0.75 mg/kg of body weight twice daily to investigate whether a similar dose optimization can safely be made	HIV+	23 HIV+	11 F<br>12 M	8.0 (7.0-9.0)*
173	2018	Jacobson	United States	Analytic<br>Case-control	To evaluate the relationship between body composition and bone mass in HIV-infected and HIV-uninfected children and youth	HIV+<br>HIV-	236 HIV+<br><br>143 HIV-	112 F<br>124 M<br>60 F<br>83 M	11.9 (7.1- 24.9)*<br><br>12.6 (7.1- 22.8)*
174	2018	Puthanakit	Thailand	Analytic<br>Experimental	To assess the changes in BMD during periods without and with calcium and vitamin D supplementation among HIV-infected adolescents with low BMD	HIV+	94 HIV+	48 F<br>46 M	14.3 (13.0-15.5)*
175	2018	Ramteke	South Africa	Analytic<br>Case-control	To compare the lipid profiles, growth, and body composition of HIV-infected children, stratified by treatment regimen, to a control group of uninfected children from the same population	HIV+<br>HIV-	553 HIV+<br><br>300 HIV-	298 F<br>255 M<br>138 F<br>162 M	6.9 (5.6-7.8)*<br><br>7.0 (5.3-8.1)*
176	2018	Rosales	Mexico	Descriptive<br>Correlational	To describe frequency of lipodystrophy secondary to ART in HIV-infected children	HIV+	49 HIV+	19 F<br>30 M	11.0 (6.0-17.0)*
177	2018	Sharma	United States	Analytic<br>Case-control	To evaluate changes in body composition measured by DXA in a cohort of HIV-infected youth compared with HIV-uninfected controls over a 7-year period	HIV+<br>HIV-	156 HIV+<br><br>79 HIV-	86 F<br>70 M<br>31 F<br>48 M	14.0 (12.2-14.1)*<br><br>13.1 (13.3-14.7)*
178	2018	Shiau	South Africa	Analytic<br>Case-control	To evaluate the relationships between immune activation, bone turnover, and bone mass in virally suppressed HIV-infected children and HIV-uninfected children	HIV+<br>HIV-	219 HIV+<br><br>180 HIV-	112 F<br>107 M<br>81 F	6.4 (1.2)<br><br>7.1 (1.6)

								99 M	
179	2018	Strehlau	South Africa	Analytic<br>Clinical trial	To evaluate the effects of preemptive substitution of abacavir for stavudine in children initially without lipodystrophy and virally suppressed on a stavudine-containing regimen	HIV+	213 HIV+	112 F<br>101 M	4.2 (1.0)
180	2018	Torrejón	Chile	Descriptive<br>Correlational	To evaluate BMD in vertically HIV-infected children	HIV+	53 HIV+	29 F<br>24 M	12.9 (8-18.5)*<br>13.6 (8.3-18.4)*
181	2019	Alves Júnior	Brazil	Descriptive<br>Correlational	To verify the association between anthropometric indicators and body fat percentage estimated by DXA and air displacement plethysmography in HIV children	HIV+	62 HIV+	34 F<br>28 M	12.8 (NR)
182	2019	Arpadi	South Africa	Analytic<br>Case-control	To measure bone quality at the calcaneus among South African children with HIV over a 2-year period by quantitative ultrasound	HIV+<br>HIV-	220 HIV+<br><br>220 HIV-	112 F<br>108 M<br>100 F<br>120 M	6.4 (1.3)<br><br>7.0 (1.5)
183	2019	de Lima	Brazil	Descriptive<br>Correlational	To investigate if moderate to vigorous physical activity and aerobic fitness are associated with cardiovascular risk factors in HIV-infected children and adolescents	HIV+	65 HIV+	35 F<br>30 M	12.2 (2.1)
184	2019	de Lima	Brazil	Descriptive<br>Method Validity	To examine the capacity of physiological variables and performance to predict peak oxygen consumption in children and adolescents living with HIV	HIV+	65 HIV+	35 F<br>30 M	12.0 (10.0-13-0)*
185	2019	Dona	Italy	Descriptive<br>Correlational	To assess the prevalence of bone homeostasis alterations in a group of vertically infected patients	HIV+	47 HIV+	35 F<br>32 M	19.2 (NR)
186	2019	Gregson	Zimbabwe	Analytic<br>Case-control	To investigate differences in bone and muscle mass and muscle function between HIV-infected and HIV-uninfected children	HIV+<br>HIV-	97 HIV+<br><br>77 HIV-	51 F<br>46 M<br>40 F<br>37 M	12.7 (2.5)<br><br>10.0 (2.9)
187	2019	Malete	Botswana	Analytic<br>Case-control	To examine differences in physical activity behaviors as a function of HIV status and sex, to test differences in physical activity self-efficacy, body weight satisfaction, and enjoyment of physical activity as a function of HIV status of youth with and without HIV	HIV+<br>HIV-	88 HIV+<br><br>162 HIV-	44 F<br>44 M<br>94 F<br>68 M	18.0 (1.7)<br><br>17.4 (2.4)
188	2019	Malete	Botswana	Analytic<br>Case-control	To examine the relationship between self-reported physical activity behaviors, fitness level, and cognitive functioning in an HIV+ population	HIV+<br>HIV-	88 HIV+<br><br>162 HIV-	44 F<br>44 M<br>94 F<br>68 M	18.6 (1.7)<br><br>17.45 (2.4)
189	2019	Margossian	United States<br>Puerto Rico	Analytic<br>Case-control	To evaluate associations of bone mineral metabolism marker concentrations with cardiac structure and function in a population of HIV-infected and HIV-exposed uninfected	HIV+<br>HIV-	305 HIV+<br><br>180 HIV-	164 F<br>141 M<br>92 F<br>88 M	12.9 (2.7)<br><br>11.1 (2.5)
190	2019	Marsico	Italy	Analytic<br>Case-control	To evaluate left ventricular function, epicardial adipose tissue, and intima-media thickness in children and adolescents with vertically acquired HIV infection	HIV+<br>HIV-	29 HIV+<br><br>29 HIV-	16 F<br>13 M<br>16 F<br>13 M	13.0 (9.0-18.0)*<br><br>13.6 (9.9-19.0)*
191	2019	Martins	Brazil	Descriptive<br>Correlational	To identify the association of phase angle with physical fitness in children and adolescents infected with HIV	HIV+	64 HIV+	34 F<br>30 M	12.1 (2.0)
192	2019	de Souza	Brazil	Analytic<br>Case-control	To evaluate the influence of body composition on the respiratory muscle strength of Amazonian children exposed to ART	HIV+<br>HIV-	29 HIV+<br><br>31 HIV-	16 F<br>13 M<br>14 F<br>17 M	7.8 (2.4)

193	2020	Jacobson	United States<br>Brazil	Analytic<br>Clinical trial	To evaluate the safety of alendronate, an oral bisphosphonate, and its effect on BMD in children and adolescents with HIV infection and low BMD	HIV+	50 HIV+	16 F<br>34 M	16.1 (11.1-23.4)*<br>16.3 (11.2-22.4)*
194	2020	Jacobson	United States<br>Puerto Rico	Analytic<br>Case-control	To compare lifetime fracture rates by HIV status and evaluate the association of ART use with fractures in HIV-infected children and adolescents	HIV+<br>HIV-	451 HIV+<br><br>277 HIV-	220 F<br>192 M<br>101 F<br>105 M	17.5 (7.6-22.2)*<br><br>16.7 (9.1-21.9)*
195	2020	Mahtab	South Africa	Analytic<br>Case-control	To investigate the prevalence and predictors of low BMD among South African perinatally HIV-infected adolescents on ART	HIV+<br>HIV-	407 HIV+<br><br>92 HIV-	205 F<br>202 M<br>50 F<br>42 M	14.0 (12.7-15.3)*<br><br>13.7 (12.0-15.3)*
196	2020	McHugh	Zimbabwe<br>Malawi	Descriptive<br>Correlational	To describe the features of HIV-associated chronic lung disease in older children and adolescents living with HIV and to examine the clinical factors associated with chronic lung disease	HIV+	421 HIV+	216 F<br>205 M	15.3 (12.7-17.7)*<br>15.6 (12.1-18.2)*
197	2020	Naidoo	South Africa	Analytic<br>Clinical trial	To investigate the effects of a home exercise program on the exercise endurance of children infected with HIV	HIV+	62 HIV+	36 F<br>26 M	8.7 (0.6)<br>8.3 (0.8)
198	2020	Shiau	South Africa	Analytic<br>Clinical trial	To evaluate if bone turnover levels in HIV-infected children differed between groups close to the time of switch ART	HIV+	212 HIV+	107 F<br>105 M	4.4 (NR)
199	2020	Shiau	South Africa	Analytic<br>Case-control	To compare bone architecture and strength by peripheral quantitative computed tomography in school-aged children HIV+ and uninfected children as controls in South Africa	HIV+<br>HIV-	172 HIV+<br><br>98 HIV-	111 F<br>86 M<br>37 F<br>61 M	10.2 (1.4)<br><br>10.8 (1.8)
200	2021	Alves Júnior	Brazil	Descriptive<br>Correlational	To verify whether there is difference in body fat values assessed by different methods according to the body image perception of HIV-infected children and adolescents.	HIV+	65 HIV+	35 F<br>30 M	12.2 (2.1)
201	2021	Andrade	Brazil	Descriptive<br>Correlational	To quantify the reduction of BMD with and without height adjustment	HIV+	69 HIV+	36 F<br>33 M	(5 to 19)
202	2021	Bhise	India	Descriptive<br>Correlational	To determine the bone health in HIV+ children on ART	HIV+	31 HIV+	11 F<br>20 M	13.0 (3.0)
203	2021	Braithwaite	South Africa	Analytic<br>Experimental	To evaluate bone and renal safety outcomes in virologically suppressed adolescents HIV+ after switching to tenofovir disoproxil fumarate	HIV+	50 HIV+	26 F<br>24 M	15.5 (15.1-16.1)*
204	2021	De Medeiros	Brazil	Analytic<br>Experimental	To analyze the influence of dietary counseling and physical activity on biochemical and metabolic parameters in children and adolescents with HIV	HIV+	10 HIV+	7 F<br>3 M	11.0 (4.7)
205	2021	Dobe	Mozambique	Descriptive<br>Correlational	To investigate the risk factors for cardiovascular disease in HIV infected children with sustained viral suppression in a low income country in Africa	HIV+	77 HIV+	35 F<br>42 M	10.0 (8.6-12.0)*
206	2021	Giacomet	Italy	Analytic<br>Experimental	To evaluate body composition and glycolipid metabolism in adolescents living with HIV starting a dolutegravir-based regimen	HIV+	13 HIV+	11 F<br>2 M	15.0 (12-19)*
207	2021	Jacobson	United States	Analytic<br>Case-control	To describe distributions of immune markers in children and young adults by sex and HIV status, and within groups, investigate associations of immune markers with bone density across Tanner stage	HIV+<br>HIV-	229 HIV+<br><br>124 HIV-	110 F<br>119 M<br>53 F<br>71 M	12.5 (10.1-14.8)*<br>12.8 (11.0-15.4)*<br>11.7 (9.1-13-8)*<br>11.9 (8.8-14.0)*
208	2021	Lindsey	United States<br>Brazil	Analytic<br>Clinical trial	To assess the safety of 96 weeks of ART with alendronate	HIV+	50HIV+	16 F<br>34 M	16.3 (11.1-23.4)*
209	2021	Martins	Brazil	Descriptive	To investigate whether handgrip strength levels are associated with	HIV+	65 HIV+	35 F	12.7 (10.5-14.0)*

				Correlational	BMC and BMD in HIV-infected children and adolescents			30 M	
210	2021	Martins	Portugal	Descriptive Correlational	To assess the nutritional status, physical activity, and quality of life in HIV-infected children and adolescents	HIV+	31 HIV+	NR	14.1 (3.5)
211	2021	Potterton	South Africa	Analytic Case-control	To determine the muscle strength of children perinatally infected with HIV compared with an uninfected control group	HIV+ HIV-	175 HIV+<br>171 HIV-	91 F 84 M<br>78 F 93 M	9.3 (1.9)<br>9.2 (1.9)
212	2021	Rukuni	Zimbabwe	Analytic Case-control	To investigate the association of HIV with bone density adjusted for skeletal size in peripubertal children	HIV+ HIV-	303 HIV+<br>306 HIV-	151 F 152 M<br>155 F 151 M	12.4 (2.5)<br>12.5 (2.5)
213	2021	Shen	South Africa	Analytic Comparative	To evaluated longitudinal trends and associations between bone mass, bone turnover and inflammatory markers among South African children living with HIV and controls	HIV+ HIV-	220 HIV+<br>220 HIV-	112 F 108 M<br>100 F 120 M	6.4 (1.2)<br>7.0 (1.5)
214	2021	Su	South Africa	Analytic Case-control	To compare total body and regional fat distribution in children HIV+ on suppressive ART regimens with controls	HIV+ HIV-	219 HIV+<br>219 HIV-	112 F 107 M<br>99 F 120 M	6.4 (1.2)<br>7.0 (1.5)
215	2021	Sudjaritruk	Thailand	Analytic Open label Clinical trial	To evaluate the impact of vitamin D and calcium supplementation on BMD and bone metabolism among HIV-infected Thai adolescents	HIV+	187 HIV+	88 F<br>99 M	16.4 (14.5-18.0)*<br>15.7 (14.4-17.5)*
216	2022	Alves Júnior	Brazil	Descriptive Correlational	To test the associations between anthropometric indicators and insulin resistance among children and adolescents diagnosed with HIV	HIV+	65 HIV+	35 F<br>30 M	12.2 (2.1)<br>12.2 (2.2)
217	2022	Chirindza	Mozambique	Descriptive Correlational	To determine the body composition, physical fitness and habitual physical activity of children and adolescents living with HIV on ART	HIV+	79 HIV+	36 F 33 M	(8 to 14)
218	2022	de Castro	Brazil	Descriptive Method Validity	To developed equations to estimate the BMC in children and adolescents diagnosed with HIV	HIV+	64 HIV+	35 F 29 M	12.2 (2.1)
219	2022	Dirajlal-Fargo	United States Puerto Rico	Analytic Observational	To investigate the association between gut dysfunction and body fat composition in youth with perinatal HIV	HIV+	261 HIV+	129 F 132 M	12.1 (9.9-14.1)*
220	2022	Mahtab	South Africa	Analytic Case-control	To investigate the association of mental health measures with metabolic outcomes	HIV+ HIV-	203 HIV+<br>44 HIV-	106 F 97 M<br>24 F 20M	10.7 (9.9-11.4)*<br>10.3 (9.7-11.1)*
221	2022	Martins	Brazil	Descriptive Correlational	To verify the association between lean mass and hand grip strength in HIV-infected children using ART (with or without PI) or not using ART	HIV+	65 HIV+	NR	12.4 (1.95)
222	2022	Martins	Brazil	Descriptive Correlational	To verify the association between fat free mass and lean mass with hand grip strength	HIV+	65 HIV+	35 F<br>30 M	12.2 (2.1)<br>12.2 (2.2)
223	2022	Melin	United Kington	Analytic Case-control	To investigate the levels and predictors of arterial stiffness in young people living with perinatal HIV and HIV negative young people	HIV+ HIV-	213 HIV+<br>65 HIV-	128 F 85 M<br>45 F 20 M	18 (16-20)*<br>18 (16-21)*
224	2022	Metgud	India	Analytic Case-control	To determine the muscle strength, flexibility, and cardiorespiratory endurance in children with HIV and to compare it with age and gender matched typically developing children	HIV+ HIV-	55 HIV+<br>55 HIV-	22 F 33 M<br>22 F 33 M	13.3 (2.8)<br>13.3 (2.8)
225	2022	Potterton	South Africa	Analytic	To investigate the sub-maximal endurance of children living with HIV	HIV+	175 HIV+	91 F	9.1 (1.8)

				Case-control	compared to a non-infected comparison group	HIV-	 171 HIV-	84 M<br>78 F<br>93 M	9.4 (2.0)<br>9.1 92.0)<br>9.3 (1.9)
226	2022	Rego	South Africa	Descriptive<br>Correlational	To determine the motor function, muscle strength and health-related quality of life in children aged 5–10 years who were perinatally infected with HIV	HIV+	30 HIV+	13 F<br>17 M	7.8 (1.7)
227	2022	Roberts	South Africa	Descriptive<br>Method Validity	To evaluates the correlations over one year between two different methods of Quantitative Ultrasound and DXA in HIV-infected young South African	HIV+<br>HIV-	80 HIV+<br><br>90 HIV-	40 F<br>40 M<br>39 F<br>51 M	7.1 (1.4)<br><br>7.3 (1.5)
228	2022	Rose	South Africa	Analytic<br>Case-control	To evaluate the prevalence and risk factors for hepatic steatosis in South African children with perinatally acquired HIV who started treatment early and remain on long-term ART compared to HIV-uninfected children	HIV+<br>HIV-	110 HIV+<br><br>105 HIV-	57 F<br>53 M<br>47 F<br>58 M	14.1 (12.7-14.9)*
229	2022	Vargas	Brazil	Descriptive<br>Correlational	To evaluate bone mass in pediatric patients infected with HIV	HIV+	46 HIV+	26 F<br>20 M	7.7 (3.5)
230	2022	Zanlorenci	Brazil	Descriptive<br>Correlational	To investigate physical growth parameters associated with BMD and BMC among children and adolescents diagnosed with HIV	HIV+	63 HIV+	35 F<br>28 M	12.1 (1.2)
231	2022	Zanlorenci	Brazil	Descriptive<br>Correlational	To verify the prevalence and factors associated with body image dissatisfaction in children and adolescents diagnosed with HIV infection	HIV+	60 HIV+	32 F<br>28 M	11.9 (1.9)<br>12.0 (2.1)
232	2023	Alves Júnior	Brazil	Descriptive<br>Correlational	To identify the discriminatory capacity of anthropometric parameters for high body fat in children and adolescents with HIV	HIV+	65 HIV+	35 F<br>30 M	12.8 (8.9)
233	2023	Alves Júnior	Brazil	Descriptive<br>Correlational	To verify the presence of difference in the lipid and glycemic profile in relation to different total body and trunk fat phenotypes in children and adolescents diagnosed with HIV	HIV+	62 HIV+	34 F<br>28 M	12.2 (2.1)
234	2023	Comley-White	South Africa	Analytic<br>Case-control	To establish the physical sequelae of perinatal HIV in adolescents	HIV+<br>HIV-	147 HIV+<br><br>102 HIV-	74 F<br>73 M<br>42 F<br>60 M	12 (2)<br><br>12 (1)
235	2023	Davies	South Africa	Analytic<br>Case-control	To evaluate the longitudinal trajectory of insulin resistance and dyslipidemia in children living with HIV and HIV-exposed uninfected children, compared with children HIV-unexposed	HIV+<br>HIV-	141 HIV+<br><br>344 HIV-	77 F<br>64 M<br>163 F<br>181 M	8.7 (8.4-9.52)*<br><br>9.4 (8.1-10.58)*
236	2023	Dirajlal-Fargo	United States<br>Puerto Rico	Descriptive<br>Correlational	To assess the association of changes in adiposity over 2 years with metabolic outcomes in young people living with HIV	HIV+	232 HIV+	122 F<br>110 M	12.25 (9.7-14.2)*
237	2023	Franco-Oliva	Mexico	Analytic<br>Case-control	To compare the resting energy expenditure of asymptomatic HIV-infected pediatric patients with healthy counterparts and to compare body composition, dietary intake, and physical activity between the two groups	HIV+<br>HIV-	39 HIV+<br><br>39 HIV-	14 F<br>25 M<br>14 F<br>25 M	11.6 (3.5)<br><br>11.6 (3.4)
238	2023	Gregson	Zimbabwe	Analytic<br>Case-control	To investigate the effect of HIV infection on muscle mass and function in peripubertal children established on ART and to what extent any identified deficits could be explained by impaired muscle quality	HIV+<br>HIV-	303 HIV+<br><br>306 HIV-	151 F<br>152 M<br>155 F<br>151 M	12.4 (2.6)<br>12.2 (2.5)<br>12.6 (2.5)<br>12.4 (2.5)
239	2023	Iheme	Nigeria	Descriptive<br>Correlational	To evaluate the health-related quality of life and nutritional status of adolescents and adults living with HIV/AIDS	HIV+	100 HIV+	52 F<br>48 M	(11 to 19)
240	2023	Maina	Kenya	Descriptive	To examine the direct effects of stunting on cognitive outcomes and	HIV+	328 HIV+	148 F	9.56 (1.2)

				Correlational	the extent to which stunting (partially) mediates the effects of HIV, age, and gender on cognitive outcomes	HIV-	260 HIV-	125 M<br>163 F<br>166 M	9.41 (1.4)
241	2023	Martins	Brazil	Descriptive<br>Correlational	To investigate how phase angle is associated with body composition in children and adolescents with HIV according to sex	HIV+	64 HIV+	35 F<br>29 M	12.2 (2.1)<br>12.2 (2.2)
242	2023	Mukwasi-Kahari	Zimbabwe	Analytic<br>Case-control	To determine the association between chronic HIV infection and bone architecture (density, size, strength) in peripubertal children	HIV+<br>HIV-	303 HIV+<br>301 HIV-	151 F<br>152 M<br>155 F<br>151 M	12.4 (2.6)<br>12.5 (2.5)<br>12.6 (2.5)<br>12.4 (2.5)
243	2023	Natukunda	Uganda	Descriptive<br>Correlational	To determine the prevalence and factors associated with low bone mass density among children living with HIV	HIV+	159 HIV+	80 F<br>79 M	10.0 (7.0-12.0)*
244	2023	Olibamoyo	Nigeria	Analytic<br>Case-control	To determine the mean serum vitamin E levels and its associations with the immunologic status, the nutritional status, and the use of highly active antiretroviral drugs in children with HIV	HIV+<br>HIV-	70 HIV+<br>70 HIV-	35 F<br>35 M<br>35F<br>35M	7.3 (3.8-10.0)*<br>7.3 (3.8-10.0)*
245	2023	Rehman	Zimbabwe<br>Malawi	Analytic<br>Case-control	To characterize growth relative to population norms among adolescents in southern Africa	HIV+<br>HIV-	303 HIV+<br>306 HIV-	151 F<br>152 M<br>155 F<br>151M	12.4 (2.5)<br>12.5 (2.5)
246	2023	Rukuni	Zimbabwe	Analytic<br>Case-control	To determine the prevalence of self-reported fracture, associated risk factors and disability, by HIV status in Zimbabwean children	HIV+<br>HIV-	303 HIV+<br>306 HIV-	151 F<br>152 M<br>155 F<br>151 M	12.5 (2.5)<br>12.5 (2.5)

SD: standard deviation; HIV or HIV-1: human immunodeficiency virus; HIV+: HIV-diagnosed; HIV-: without HIV infection diagnosis; F: females; M: males; NR: not reported; PI: protease inhibitors; ART: antiretroviral therapy; BIA: bioelectrical impedance analysis; DXA: dual energy X-ray absorptiometry; AIDS: acquired immunodeficiency syndrome; VO2: oxygen consumption; *median and age range.

Supplementary Table S2. Health-related physical fitness components investigated and physical activity level (protocols/tests and cut-points applied)

Nº	Year	First Author	Groups	Investigated component (s)	Investigated	Method/protocol	Cut-points	PA level method/protocol	PA level Cut-points
1	1995	Miller	HIV+	Body composition	Changes in Body composition	Anthropometric: body mass, height, body mass-for-height, Triceps SF, arm relaxed circumference, arm muscular circumference	Growth curves (NCHS/WHO)	NI	NI
2	1995	Saavedra	HIV+<br>HIV-	Body composition	Changes in Body composition	Anthropometric: height, body mass, head circumference	Growth curves (NCHS/WHO)	NI	NI
3	1996	Arpadi	HIV+	Body composition	Method validity	Anthropometric: body mass, height;<br>BIA: total body water; fat-free mass;<br>Deuterium dilution: total body water;<br>DXA: fat-free mass	NR	NI	NI
4	1997	Miller	HIV+	Body composition	Associations	Anthropometric: body mass, height, body mass-for-height, Triceps SF, arm relaxed circumference, arm muscular circumference	Growth curves (NCHS/WHO); Ten-State Nutrition Survey (arm muscular circumference and Triceps SF)	NI	NI
5	1998	Arapadi	HIV+<br>HIV-	Body composition	Groups comparison	Anthropometric: body mass, height, Triceps SF, arm relaxed circumference, arm muscular circumference;<br>DXA: body fat mass, body fat percentage, fat-free mass	NR	NI	NI
6	1998	Henderson	HIV+<br>HIV-	Body composition	Groups comparison	Anthropometric: body mass, height, Triceps SF, arm relaxed circumference, arm muscular circumference;<br>Deuterium dilution: total body water	Growth curves (NCHS/WHO)	NI	NI
7	1999	Fontana	HIV+<br>HIV-	Body composition	Groups comparison	Anthropometric: body mass, height, arm relaxed circumference, Triceps SF, arm muscular circumference, arm area;<br>BIA: fat-free mass	Z-scores	NI	NI
8	1999	Fox-Wheeler	HIV+	Body composition	Changes in Body composition	Anthropometric: body mass, height, Triceps SF, arm relaxed circumference, arm muscular circumference, BMI;<br>Computed Tomography: bone area	Not applied	NI	NI
9	2000	Arpadi	HIV+	Body composition	Associations	Anthropometric: body mass, height;<br>DXA: fat-free mass;<br>Deuterium dilution: total body water	Percentiles	NI	NI
10	2000	Fiore	HIV+	Body composition	Associations	Anthropometric: body mass, height, BMI	Percentiles	NI	NI
11	2000	Heller	HIV+	Body composition	Method validity	Anthropometric: body mass, height, BMI, Triceps SF, arm relaxed circumference, arm muscular circumference	Growth curves (NCHS/WHO)	NI	NI
12	2000	Jansen	HIV+<br>HIV-	Body composition	Associations	Anthropometric: body mass, height, Triceps SF, arm relaxed circumference, arm muscular circumference, body fat percentage	Growth curves (NCHS/WHO)	NI	NI

13	2000	Jaquet	HIV+	Body composition	Prevalences	Anthropometric: body mass, height, BMI, Biceps SF, Triceps SF, Suprailiac SF, Subscapular SF	French Growth curves	NI	NI
14	2000	Keyser	HIV+	Body composition<br>Cardiorespiratory fitness	Prevalences	Anthropometric: body mass, height<br>Maximum effort treadmill test:<br>heart rate; peak oxygen consumption	Not applied<br><br>ACSM Guidelines	NI	NI
15	2000	Mismer	HIV+	Body composition	Changes in Body composition	Anthropometric: body mass, height, Triceps SF, arm relaxed circumference, arm muscular circumference	Growth curves (NCHS/WHO);<br>Ten-State Nutrition Survey (arm muscular circumference e Triceps SF)	NI	NI
16	2001	Arpadi	HIV+	Body composition	Changes in Body composition	Anthropometric: body mass, height, BMI;<br>DXA: body fat mass	Z-scores	NI	NI
17	2001	Brambilla	HIV+<br>HIV-	Body composition	Groups comparison	Anthropometric: body mass, height, BMI;<br>DXA: body fat mass, lean mass;<br>Magnetic Resonance Imaging: visceral fat	Not applied	NI	NI
18	2001	Dreimane	HIV+	Body composition	Changes in Body composition	Anthropometric: body mass, height	Not applied	NI	NI
19	2001	Ellis	HIV+<br>HIV-	Body composition	Method validity	Anthropometric: body mass, height, BMI;<br>DXA: bone mass content, bone mass density, bone area	Not applied	NI	NI
20	2001	Melvin	HIV+	Body composition	Groups comparison	Anthropometric: body mass, height, BMI, waist circumference, hip circumference, waist-to-height ration, Triceps SF, Subscapular SF, Abdominal SF, Thigh SF;<br>DXA: body fat mass, lean mass, bone mass density	Not applied	NI	NI
21	2001	Miller	HIV+	Body composition	Changes in Body composition	Anthropometric: body mass, height, body mass-for-height, Triceps SF, arm relaxed circumference, arm muscular circumference	Growth curves (NCHS/WHO);<br>Ten-State Nutrition Survey (arm muscular circumference e Triceps SF)	NI	NI
22	2001	Mora	HIV+<br>HIV-	Body composition	Groups comparison	Anthropometric: body mass, height, BMI;<br>DXA: bone mass density	Not applied	NI	NI
23	2001	O'Brien	HIV+	Body composition	Associations	Anthropometric: body mass, height, BMI;<br>DXA: body fat percentage, lean mass, bone mass content, bone mass density	Growth curves (NCHS/WHO);<br>Previous study (sample HIV-)	NI	NI
24	2001	Tan	HIV+<br>HIV-	Body composition	Groups comparison	Anthropometric: body mass, height	Growth curves (NCHS/WHO)	NI	NI
25	2002	Amaya	HIV+	Body composition	Associations	Anthropometric: body mass, height, BMI, hip circumference, abdominal girth	Not applied	NI	NI
26	2002	Arpadi	HIV+	Body composition	Groups comparison	Anthropometric: body mass, height, BMI;	Z-scores	NI	NI

			HIV-			DXA: bone mass content			
27	2002	Cade	HIV+<br>HIV-	Body composition<br>Cardiorespiratory fitness	Groups comparison	Anthropometric: body mass, height, BMI, lean body mass index;<br>Maximum effort treadmill test: heart rate peak; peak oxygen consumption	Not applied	NR	NR
28	2002	Cossarizza	HIV+<br>HIV-	Body composition	Groups comparison	Anthropometric: body mass, height, BMI;<br>DXA: body fat mass, lean mass	Not applied	NI	NI
29	2002	Horlick	HIV+<br>HIV-	Body composition	Method validity	Anthropometric: body mass, height, BMI;<br>DXA: fat-free mass;<br>Deuterium dilution: total body water;<br>BIA: resistance, impedance	Z-scores	NI	NI
30	2002	Nachman	HIV+	Body composition	Groups comparison	Anthropometric: body mass, height	Growth curves (NCHS/WHO)	NI	NI
31	2002	Rondanelli	HIV+<br>HIV-	Body composition	Groups comparison	Anthropometric: body mass, height, BMI, Biceps SF, Triceps SF, Suprailiac SF, Subscapular SF, arm relaxed circumference, arm muscular circumference	Not applied	Structured questionnaire	Not applied
32	2002	Verweel	HIV+	Body composition	Changes in Body composition	Anthropometric: body mass, height, BMI	Dutch Growth curves	NI	NI
33	2003	Beregszaszi	HIV+<br>HIV-	Body composition	Groups comparison	Anthropometric: body mass, height, BMI, Suprailiac SF, Biceps SF, Triceps SF, Subscapular SF;<br>BIA: body fat percentage	French Growth curves	NI	NI
34	2003	Bitnun	HIV+	Body composition	Groups comparison	Anthropometric: body mass, height, BMI, waist circumference, hip circumference, waist-to-hip ratio;<br>Computed Tomography:<br>visceral fat	Not applied	NI	NI
35	2003	Bockhorst	HIV+	Body composition	Groups comparison	Anthropometric: body mass, height, BMI	Not applied	NI	NI
36	2003	McComsey	HIV+	Body composition	Changes in Body composition	Anthropometric: body mass, height, BMI, Suprailiac SF, Biceps SF, Triceps SF, Subscapular SF, thigh circumference, arm relaxed circumference, Triceps SF, Thigh SF, waist-to-hip ratio;<br>BIA: body fat mass, body fat percentage, lean mass, lean mass percentual	Not applied	NI	NI
37	2003	Vigano	HIV+	Body composition	Groups comparison	Anthropometric: body mass, height, BMI;<br>DXA: body fat mass, body fat percentage, lean mass;<br>Magnetic Resonance Imaging: visceral fat	Not applied	NI	NI
38	2003	Vigano	HIV+<br>HIV-	Body composition	Groups comparison	Anthropometric: body mass, height, BMI;<br>DXA: body fat mass, body fat percentage, lean mass;<br>Magnetic Resonance Imaging: visceral fat	Not applied	NI	NI
39	2003	Zamboni	HIV+	Body composition	Associations	Anthropometric: body mass, height, BMI;<br>DXA: bone mass density	Previous study (sample HIV-)	NI	NI
40	2004	Ghaffari	HIV+	Body composition	Groups comparison	Anthropometric: body mass, height	Growth curves	NI	NI

							(NCHS/WHO)		
41	2004	Hardin	HIV+ HIV-	Body composition	Groups comparison	Anthropometric: body mass, height; DXA: lean mass	Not applied	NI	NI
42	2004	Mora	HIV+ HIV-	Body composition	Groups comparison	Anthropometric: body mass, height; DXA: bone mass density	Z-scores	NR	Not applied
43	2004	Panamonta	HIV+	Body composition	Associations	Anthropometric: body mass, height	Thai reference values	NI	NI
44	2004	Rojo	HIV+	Body composition	Associations	Anthropometric: body mass, height, BMI; DXA: bone mass density	(bone mass density) Osteoporosis WHO taskforce	NI	NI
45	2004	Stagi	HIV+ HIV-	Body composition	Groups comparison	Anthropometric: body mass, height, BMI	Previous study (sample HIV-)	NI	NI
46	2004	Taylor	HIV+	Body composition	Groups comparison	Physical examination: visual inspection of fat distribution	Not applied	NI	NI
47	2004	Thotne	HIV+	Body composition	Associations	Anthropometric: body mass, height, BMI	Not applied	NI	NI
48	2005	Aldámiz-Echevarría	HIV+ HIV-	Body composition	Groups comparison	Anthropometric: body mass, height, BMI	Spanish Growth curves	NI	NI
49	2005	Bitnun	HIV+	Body composition	Associations	Anthropometric: body mass, height, BMI, waist circumference, hip circumference, waist-to-hip ratio	Previous study (sample HIV-)	NI	NI
50	2005	Giacomet	HIV+ HIV-	Body composition	Groups comparison	DXA: bone mass density, bone mass content	Not applied	NI	NI
51	2005	Hardin	HIV+	Body composition	Changes in Body composition	Anthropometric: body mass, height, BMI; DXA: lean mass	Z-scores	NI	NI
52	2005	Hazra	HIV+	Body composition	Changes in Body composition	Anthropometric: body mass, height; DXA: bone mass density	(bone mass density) Previous study (sample HIV-)	NI	NI
53	2005	Jacobson	HIV+ HIV-	Body composition	Groups comparison	Anthropometric: body mass, height, BMI, arm relaxed circumference, Triceps SF, arm muscular circumference; DXA: bone mass density	Growth curves (NCHS/WHO) *Ten-State Nutrition Survey* (arm muscular circumference e Triceps SF)	NI	NI
54	2005	Mora	HIV+ HIV-	Body composition	Groups comparison	Anthropometric: body mass, height, BMI; DXA: bone mass content	Not applied	NI	NI
55	2005	Pitukcheewanont	HIV+ HIV-	Body composition	Groups comparison	Anthropometric: body mass, height, BMI; DXA: bone mass density; Computed Tomography: bone mass density e bone area	Z-scores	NI	NI
56	2005	Rosso	HIV+ HIV-	Body composition	Groups comparison	Anthropometric: body mass, height, BMI; DXA: bone mass density;	Previous study (sample HIV-)	NI	NI
57	2005	Vigano	HIV+ HIV-	Body composition	Groups comparison	Anthropometric: body mass, height, BMI; DXA: body fat mass, lean mass;	Not applied	NI	NI

						Magnetic Resonance Imaging: visceral fat			
58	2006	Barros	HIV+	Body composition Muscular strength/enduran ce	Associations	Anthropometric: body mass, height, Biceps SF, Suprailiac SF, Subscapular SF; Triceps SF; Axilla SF, Calf SF, calf circumference, arm contract circumference;<br>Muscular strength/endurance: vertical jump, horizontal jump	Previous study (sample HIV-)	NI	NI
59	2006	Ergun-Longmire	HIV+	Body composition	Groups comparison	Anthropometric: body mass, height, BMI, Triceps SF, Biceps SF, Abdominal SF, waist circumference, hip circumference	Z-scores	NI	NI
60	2006	Gafni	HIV+	Body composition	Associations	Anthropometric: body mass, height, BMI;<br>DXA: bone mass density	Growth curves (NCHS/WHO)	NI	NI
61	2006	Gutiérrez	HIV+	Body composition	Associations	Anthropometric: body mass, height, BMI, Triceps SF, Subscapular SF	Spanish Growth curves	NI	NI
62	2006	Haroun	HIV+<br>HIV-	Body composition	Groups comparison	Anthropometric: body mass, height, BMI, Triceps SF, Subscapular SF, waist circumference	British Growth curves	NI	NI
63	2006	Hartman	HIV+	Body composition	Groups comparison	Anthropometric: body mass, height, BMI, Biceps SF, Triceps SF, Subscapular SF, Suprailiac SF, arm relaxed circumference, calf circumference; waist circumference, hip circumference	Dutch Growth curves	NI	NI
64	2006	Moscocki	HIV+<br>HIV-	Body composition	Groups comparison	Anthropometric: body mass, height, BMI, Biceps SF, Triceps SF, Subscapular SF, Suprailiac SF, arm relaxed circumference, arm muscular circumference	NHANES III	NI	NI
65	2006	Verkauskiene	HIV+	Body composition	Groups comparison	Anthropometric: body mass, height, BMI, Biceps SF, Triceps SF, Subscapular SF, Suprailiac SF;<br>BIA: body fat percentage	French Growth curves curves	NI	NI
66	2006	Weidle	HIV+	Body composition	Associations	Anthropometric: body mass, height	Not applied	NI	NI
67	2007	Chantry	HIV+<br>HIV-	Body composition	Groups comparison	Anthropometric: body mass, height, BMI	Growth curves (NCHS/WHO)	NI	NI
68	2007	Dzwonek	HIV+	Body composition	Associations	Anthropometric: body mass, height, BMI, Triceps SF, Biceps SF, Subscapular SF, Suprailiac SF, arm relaxed circumference, waist circumference, hip circumference, calf circumference	(BMI) British Growth curves<br>(SF) Dutch Growth curves	NI	NI
69	2007	Ene	HIV+	Body composition	Groups comparison	Anthropometric: body mass, height, arm relaxed circumference, hip circumference, abdominal girth	Not applied	Structured questionnaire	Not applied
70	2007	Kim	HIV+	Body composition	Groups comparison	Anthropometric: body mass, height, BMI	Growth curves (NCHS/WHO)	NI	NI
71	2007	McComsey	HIV+<br>HIV-	Body composition	Groups comparison	Anthropometric: body mass, height, waist circumference, hip circumference, waist-to-height ration	Not applied	NI	NI
72	2007	Mora	HIV+	Body composition	Groups comparison	Anthropometric: body mass, height, BMI;	Italian Growth	NI	NI

			HIV-			DXA: bone mass density	curves		
73	2007	Papaevangelou	HIV+<br>HIV-	Body composition	Groups comparison	Anthropometric: body mass, height, BMI	Not applied	NI	NI
74	2007	Tremechin	HIV+<br>HIV-	Body composition	Groups comparison	Anthropometric: body mass, height, BMI, arm relaxed circumference, Triceps SF, Subscapular SF;<br>BIA: fat-free mass, total body water	Growth curves (NCHS/WHO)	NI	NI
75	2007	Vigano	HIV+<br>HIV-	Body composition	Changes in Body composition	Anthropometric: body mass, height, BMI;<br>DXA: body fat mass, lean mass	Not applied	NI	NI
76	2007	Vigano	HIV+<br>HIV-	Body composition	Changes in Body composition	Anthropometric: body mass, height, BMI;<br>DXA: lean mass	Not applied	NI	NI
77	2008	Chantry	HIV+<br>HIV-	Body composition	Groups comparison	Anthropometric: body mass, height, BMI, waist circumference, hip circumference, arm relaxed circumference, arm muscular circumference, Triceps SF, Thigh SF, Subscapular SF	NHANES 99-00 e 01-02	NI	NI
78	2008	Gonzales-Tome	HIV+	Body composition	Changes in Body composition	Anthropometric: body mass, height, arm relaxed circumference, Triceps SF, Subscapular SF;<br>DXA: bone mass density, lean mass, body fat percentage	(bone mass density) Previous study (sample HIV-)<br>(bone mass density) World Health Organization Taskforce for Osteoporosis	NI	NI
79	2008	Miller	HIV+<br>HIV-	Body composition	Groups comparison	Anthropometric: body mass, height, BMI, waist circumference, hip circumference, arm relaxed circumference, Triceps SF, arm muscular circumference	NHANES III	NI	NI
80	2008	Purdy	HIV+	Body composition	Changes in Body composition	Anthropometric: body mass, height;<br>DXA: bone mass density	Previous study (sample HIV-)	NI	NI
81	2008	Sharma	HIV+	Body composition	Changes in Body composition	Anthropometric: body mass, height, BMI, arm relaxed circumference, Triceps SF, arm muscular circumference	Growth curves (NCHS/WHO)<br>(arm muscular circumference Triceps SF) Previous study (sample HIV-)	NI	NI
82	2008	Spagnoulo	HIV+<br>HIV-	Body composition	Groups comparison	Anthropometric: body mass, height, BMI;<br>Ultrasound: visceral fat	Italian Growth curves	NI	NI
83	2009	Aldrovandi	HIV+<br>HIV-	Body composition	Groups comparison	Anthropometric: body mass, height, BMI, waist circumference, hip circumference, waist-to-height ration;<br>DXA: lean mass, body fat mass	Growth curves (NCHS/WHO)	Structured questionnaire	Days per week
84	2009	Arpadi	HIV+<br>HIV-	Body composition	Groups comparison	Anthropometric: body mass, height, BMI;<br>DXA: body fat mass, body fat percentage	Growth curves (NCHS/WHO)	NI	NI
85	2009	Lopez	HIV+	Body composition	Groups comparison	Anthropometric: body mass, height, BMI;	Z-scores	NI	NI

			HIV-			DXA: bone mass density, body fat mass		
86	2009	Mora	HIV+	Body composition	Method validity	Anthropometric: body mass, height, BMI;<br>DXA: bone mass density, bone mass content;<br>Ultrasound: speed of sound	(bone mass density, bone mass content) NHANES III	NI	NI
87	2009	Sarni	HIV+	Body composition	Associations	Anthropometric: body mass, height, BMI	Growth curves (NCHS/WHO)	NI	NI
88	2009	Vigano	HIV+	Body composition	Changes in Body composition	Anthropometric: body mass, height, BMI	Growth curves (NCHS/WHO)	NI	NI
89	2010	Cervia	HIV+	Body composition	Changes in Body composition	Anthropometric: body mass, height, waist circumference, hip circumference, Triceps SF, Thigh SF, Subscapular SF;<br>BIA: total body water, fat-free mass, body fat mass	NHANES (NR)	NI	NI
90	2010	Chantry	HIV+	Body composition	Changes in Body composition	Anthropometric: body mass, height, waist circumference, hip circumference, Triceps SF, Thigh SF, Subscapular SF, arm muscular circumference, tight muscular circumference;<br>BIA: total body water, fat-free mass, body fat mass	NHANES 99-00 e 01-02	NI	NI
91	2010	Jacobson	HIV+<br>HIV-	Body composition	Groups comparison	Anthropometric: body mass, height, BMI;<br>DXA: bone mass content, bone mass density, lean mass, body fat mass	Growth curves (NCHS/WHO)	Structured questionnaire	NR
92	2010	Miller	HIV+<br>HIV-	Body composition	Groups comparison	Anthropometric: body mass, height, BMI, hip circumference, waist circumference;<br>DXA: body fat mass, body fat percentage, lean mass	Growth curves (NCHS/WHO)	NI	NI
93	2010	Miller	HIV+	Cardiorespiratory fitness<br>Muscular strength/enduran ce<br>Body composition<br>Flexibility	Changes in: Body composition, Cardiorespiratory fitness, Muscular strength/endurance and Flexibility	Muscular strength/endurance: hand grip strength, sit-up;<br>Flexibility: sit-to-reach;<br>Maximum effort treadmill test: peak oxygen consumption, heart rate, respiratory rate;<br>Anthropometric: body mass, height, BMI, hip circumference, waist circumference;<br>DXA: body fat mass	Growth curves (NCHS/WHO)	NI	NI
94	2010	Stagi	HIV+	Body composition	Changes in Body composition	Anthropometric: body mass, height, BMI	Italian Growth curves	NI	NI
95	2010	Vigano	HIV+	Body composition	Changes in Body composition	Anthropometric: body mass, height, BMI;<br>DXA: bone mass density	Italian Growth curves	NI	NI
96	2010	Werner	HIV+	Body composition	Associations	Anthropometric: body mass, height, BMI, Triceps SF, Subscapular SF	Growth curves (NCHS/WHO)	NR	NR
97	2010	Zuccotti	HIV+<br>HIV-	Body composition	Groups comparison	Anthropometric: body mass, height, BMI;<br>DXA: bone mass content, bone mass density	Italian Growth curves<br>(bone mass density bone mass content) NHANESS (NR)	NI	NI

98	2011	Contri	HIV+	Body composition	Groups comparison	Anthropometric: body mass, height, BMI, waist circumference, arm relaxed circumference Triceps SF, Subscapular SF;<br>BIA: fat-free mass, total body water	Growth curves (NCHS/WHO) (NR)	NI	NI
99	2011	da Silva	HIV+	Body composition	Associations	Anthropometric: body mass, height, BMI	Growth curves (NCHS/WHO)	NI	NI
100	2011	Dimock	HIV+	Body composition	Associations	Anthropometric: body mass, height, BMI, waist circumference, hip circumference;<br>DXA: body fat mass, lean mass	Not applied	NI	NI
101	2011	Geffner	HIV+	Body composition	Associations	Anthropometric: body mass, height, BMI, waist circumference; hip circumference, waist-to-height ration;<br>DXA: body fat percentage	BMI Percentiles	NI	NI
102	2011	Jacobson	HIV+<br>HIV-	Body composition	Groups comparison	Anthropometric: body mass, height, BMI, Suprailiac SF, waist circumference, hip circumference, waist-to-hip ratio;<br>DXA: body fat mass, lean mass, body fat percentage	Growth curves (NCHS/WHO) (body fat mass) NHANES 199-2004	NI	NI
103	2011	Mohd	HIV+	Body composition	Associations	Anthropometric: body mass, height, BMI	Growth curves (NCHS/WHO)	NI	NI
104	2011	Morén	HIV+<br>HIV-	Body composition	Groups comparison	Physical examination: visual inspection of fat distribution	Not applied	NI	NI
105	2011	Ramalho	HIV+<br>HIV-	Body composition	Groups comparison	Anthropometric: body mass, height, BMI, waist circumference hip circumference, Triceps SF, Subscapular SF, SF ratio (Subscapular SF/Triceps SF)	Growth curves (NCHS/WHO)	NI	NI
106	2011	Resino	HIV+	Body composition	Changes in Body composition	Anthropometric: body mass, height, BMI	Growth curves (NCHS/WHO)	NI	NI
107	2011	Spoulou	HIV+<br>HIV-	Body composition	Groups comparison	Anthropometric: body mass, height, BMI;<br>DXA: body fat mass, lean mass	Z-scores	NI	NI
108	2011	Tremeschin	HIV+<br>HIV-	Body composition	Groups comparison	Anthropometric: body mass, height, BMI, arm relaxed circumference, waist circumference, Subscapular SF, Triceps SF;<br>BIA: fat-free mass total body water;<br>DXA: body fat mass, lean mass	Growth curves (NCHS/WHO)	NI	NI
109	2011	Vigano	HIV+<br>HIV-	Body composition	Groups comparison	Anthropometric: body mass, height, BMI;<br>DXA: body fat mass, lean mass	Italian Growth curves	NI	NI
110	2012	Alam	HIV+	Body composition	Associations	Physical examination: visual inspection of fat distribution	Not applied	NI	NI
111	2012	Arpadi	HIV+	Body composition	Groups comparison	Anthropometric: body mass, height, BMI;<br>DXA: bone mass content, bone mass density;	Percentiles	NI	NI
112	2012	Bhargav	HIV+	Body composition	Associations	Anthropometric: body mass, height, BMI;<br>BIA: body fat mass, body fat percentage, fat-free mass	Not applied	NI	NI
113	2012	Innes	HIV+	Body composition	Groups comparison	Anthropometric: body mass, height, BMI, arm relaxed circumference, thigh circumference, chest circumference, waist circumference; hip	Z-scores	NI	NI

						circumference, Biceps SF, Triceps SF, Subscapular SF, Thigh SF, waist-to-height ration, SF ratio; DXA: body fat mass, body fat percentage, lean mass			
114	2012	Lindsey	HIV+ HIV-	Body composition	Groups comparison	DXA: lean mass, body fat mass, body fat percentage	Not applied	NI	NI
115	2012	Miller	HIV+ HIV-	Body composition	Groups comparison	Anthropometric: body mass, height, BMI, waist circumference, hip circumference; DXA: body fat percentage	Growth curves (NCHS/WHO)	NI	NI
116	2012	Negra	HIV+	Body composition	Changes in Body composition	DXA: bone mass density	Not applied	NI	NI
117	2012	Puthanakit	HIV+ HIV-	Body composition	Groups comparison	Anthropometric: body mass, height, BMI; DXA: bone mass density	Growth curves (NCHS/WHO) (body mass, height) Thai reference values	NI	NI
118	2012	Ramos	HIV+ HIV-	Body composition Muscular strength/enduran ce	Groups comparison	Anthropometric: body mass, height, BMI; Muscular strength/endurance: isokinetic dynamometer	Not applied	NI	NI
119	2012	Schtscherbyna	HIV+	Body composition	Associations	Anthropometric: body mass, height, BMI; DXA: bone mass density, lean mass, body fat percentage	Growth curves (NCHS/WHO)	NI	NI
120	2013	Arpadi	HIV+	Body composition	Groups comparison	Anthropometric: body mass, height, BMI, arm relaxed circumference, thigh circumference, waist circumference, hip circumference, Biceps SF, Triceps SF, Subscapular SF, Suprailiac SF, Abdominal SF, Thigh SF, SF ratio; BIA: body fat percentage	Growth curves (NCHS/WHO)	NI	NI
121	2013	Bunders	HIV+	Body composition	Associations	Anthropometric: body mass, height, BMI; DXA: bone mass density	Growth curves (NCHS/WHO)	NI	NI
122	2013	Chokephaibulkit	HIV+	Body composition	Associations	DXA: bone mass density	Not applied	NI	NI
123	2013	DiMeglio	HIV+ HIV-	Body composition	Groups comparison	Anthropometric: body mass, height, BMI; DXA: bone mass density	Growth curves (NCHS/WHO) (bone mass density) Previous study (sample HIV-)	Structured questionnaire	Percentiles
124	2013	dos Santos	HIV+	Flexibility; Muscular strength/enduran ce	Associations	Flexibility: teste de sit-to-reach; Muscular strength/endurance: abdominal test	PROESP-BR	NI	NI
125	2013	Fabiano	HIV+	Body composition	Changes in Body composition	Anthropometric: body mass, height, BMI, waist circumference; DXA: lean mass, body fat mass, bone mass content	Italian Growth curves (Waist circumference)	NI	NI

							Previous study (sample HIV-)		
126	2013	Innes	HIV+	Body composition	Method validity	Anthropometric: body mass, height, BMI, arm relaxed circumference, thigh circumference, chest circumference, waist circumference, hip circumference, Biceps SF, Triceps SF, Suprailiac SF, Subscapular SF, Thigh SF, waist-to-height ration, SF ratio, waist-to-arm relaxed circumference ratio, body mass/arm relaxed circumference ratio	New cut-point	NI	NI
127	2013	Lima	HIV+	Body composition	Associations	Anthropometric: body mass, height, BMI;<br>DXA: bone mass density, bone mass content	Growth curves (NCHS/WHO)<br>(bone mass content, bone mass density)<br>NHANES IV	Pedometer	13,000 and 11,000 steps per day
128	2013	Macdonald	HIV+	Body composition	Associations	Anthropometric: body mass, height, BMI, tibia length;<br>DXA: bone mass content, lean mass, body fat percentage	Not applied	Structured questionnaire	NR
129	2013	Palchetti	HIV+	Body composition	Associations	Anthropometric: body mass, height, BMI, waist circumference, arm relaxed circumference, calf circumference, Triceps SF, Biceps SF, Subscapular SF, Suprailiac SF, SF ratio;<br>DXA: lean mass, body fat mass, body fat percentage	Growth curves (NCHS/WHO)	NI	NI
130	2013	Palchetti	HIV+	Body composition	Method validity	Anthropometric: body mass, height, BMI;<br>BIA: fat-free mass, body fat mass<br>DXA: fat-free mass, body fat mass	Growth curves (NCHS/WHO)	NI	NI
131	2013	Sharma	HIV+	Body composition	Associations	Anthropometric: body mass, height, BMI;	Z-scores	NI	NI
132	2013	Somarriba	HIV+<br>HIV-	Body composition<br>Cardiorespiratory fitness<br>Muscular strength/endurance<br>Flexibility	Groups comparison	Anthropometric: body mass, height, BMI, waist circumference, hip circumference, waist-to-height ration;<br>DXA, body fat mass;<br>Maximum effort treadmill test: peak oxygen consumption;<br>Muscular strength/endurance: one repetition maximum test (chest press and leg press), sit-up;<br>Flexibility: modified sit-to-reach test	Growth curves (NCHS/WHO)<br>National Presidential Fitness Program	NI	NI
133	2014	Agustinho	HIV+<br>HIV-	Body composition	Groups comparison	Anthropometric: body mass, height, BMI, waist circumference, hip circumference, Subscapular SF, Biceps SF, Triceps SF, Suprailiac SF;<br>BIA: body fat mass	Growth curves (NCHS/WHO)	NI	NI
134	2014	Dejkhamron	HIV+	Body composition	Changes in Body composition	Anthropometric: body mass, height, BMI	Growth curves (NCHS/WHO)	NI	NI
135	2014	Foissac	HIV+	Body composition	Changes in Body	Anthropometric: body mass, height, BMI	Not applied	NI	NI

					composition				
136	2014	Hillesheim	HIV+	Body composition	Associations	Anthropometric: body mass, height, BMI	Growth curves (NCHS/WHO)	NI	NI
137	2014	Humphries	HIV+	Muscular strength/endurance	Groups comparison	Anthropometric: body mass, height, BMI; Muscular strength/endurance: hand grip strength	NR	NI	NI
138	2014	Mussime	HIV+ HIV-	Body composition	Groups comparison	Anthropometric: body mass, height, arm relaxed circumference, calf circumference, Biceps SF, Triceps SF, Subscapular SF, Suprailiac SF, SF ratio	Not applied	NI	NI
139	2014	Theeodoridou	HIV+ HIV-	Body composition	Groups comparison	Anthropometric: body mass, height, BMI; DXA: body fat mass, lean mass	Not applied	NI	NI
140	2014	Vreeman	HIV+	Body composition	Associations	Anthropometric: body mass, height, arm relaxed circumference; Deuterium dilution: total body water	Not applied	NI	NI
141	2015	Aurpibul	HIV+	Body composition	Changes in Body composition	Anthropometric: body mass, height; DXA: bone mass density	(bone mass density) Thai reference values	NI	NI
142	2015	Cohen	HIV+ HIV-	Body composition	Groups comparison	Anthropometric: body mass, height, BMI; DXA: body fat mass	Z-scores	NI	NI
143	2015	Della Negra	HIV+	Body composition	Changes in Body composition	Anthropometric: body mass, height; DXA: bone mass density	Growth curves (NCHS/WHO)	NI	NI
144	2015	dos Reis	HIV+	Body composition	Associations	Anthropometric: body mass, height, BMI, arm relaxed circumference, waist circumference, neck circumference, arm muscular circumference, arm fat mass circumference, Triceps SF; BIA: body fat percentage	Growth curves (NCHS/WHO) (waist circumference) NHANES 199-2002, Previous study (sample HIV-)) (neck circumference) Previous study (sample HIV-))	NI	NI
145	2015	Mora	HIV+ HIV-	Body composition	Groups comparison	Anthropometric: body mass, height, BMI; DXA: bone mass density	Italian Growth curves	NI	NI
146	2015	Palchetti	HIV+	Body composition	Changes in Body composition	Anthropometric: body mass, height, BMI; DXA: body fat mass, lean mass, bone mass content, bone mass density	Growth curves (NCHS/WHO)	NI	NI
147	2015	Swetha	HIV+	Body composition	Associations	Anthropometric: body mass, height, BMI, SF (NR)	Growth curves (NCHS/WHO)	NI	NI
148	2016	Arpadi	HIV+ HIV-	Body composition	Groups comparison	Anthropometric: body mass, height, BMI; DXA: bone mass content, bone mass density	Growth curves (NCHS/WHO)	Structured questionnaire	WHO PA recommendations
149	2016	Gaur	HIV+	Body composition	Changes in Body composition	Anthropometric: body mass, height; DXA: bone mass density	Growth curves (NCHS/WHO)	NI	NI
150	2016	de Lima	HIV+	Body composition	Method validity	Anthropometric: body mass, height, BMI, arm relaxed circumference, humerus diameter,	Growth curves (NCHS/WHO)	NI	NI

						femur diameter, Triceps SF, arm muscular circumference;<br>DXA: bone mass content, bone mass density			
151	2016	Sonego	HIV+	Body composition	Associations	Anthropometric: body mass, height, BMI	Growth curves (NCHS/WHO)	Structured questionnaire	Days per week
152	2016	Sudjaritruk	HIV+	Body composition	Associations	Anthropometric: body mass, height, BMI;<br>DXA: bone mass density	Growth curves (NCHS/WHO);<br>(body mass, height) Thai reference values	Structured questionnaire	Not applied
153	2016	Wong	HIV+<br>HIV-	Body composition	Groups comparison	Anthropometric: body mass, height, arm relaxed circumference;<br>BIA: fat-free mass percentual	Growth curves (NCHS/WHO)	Structured questionnaire	WHO PA recommendations
154	2017	Carmo	HIV+	Body composition	Changes in Body composition	Anthropometric: body mass, height;<br>DXA: bone mass density	International Society for Clinical Densitometry	NR	Not applied
155	2017	de Lima	HIV+<br>HIV-	Body composition<br>Cardiorespiratory fitness	Groups comparison	Anthropometric: body mass, height, BMI;<br>Maximum effort cycle ergometer test : peak oxygen consumption, heart rate	Growth curves (NCHS/WHO)	Accelerometer	WHO PA recommendations
156	2017	Giacomet	HIV+<br>HIV-	Body composition	Groups comparison	Anthropometric: body mass, height, BMI;<br>DXA: bone mass density	Italian Growth curves	NI	NI
157	2017	Jacobson	HIV+<br>HIV-	Body composition	Groups comparison	Anthropometric: body mass, height, BMI;<br>DXA: bone mass density, bone mass content	Growth curves (NCHS/WHO)	Structured questionnaire	Internal (Z-scores)
158	2017	Jimenéz	HIV+	Body composition	Associations	Anthropometric: body mass, height, BMI;<br>DXA: bone mass density	Spanish Growth curves (bone mass density)<br>Spanish reference values	NI	NI
159	2017	de Lima	HIV+	Body composition	Method validity	Anthropometric: body mass, height, BMI, arm relaxed circumference, waist circumference, Subscapular SF, Triceps SF, Abdominal SF, Calf SF, SF ratio;<br>DXA: body fat percentage	Growth curves (NCHS/WHO)<br>(body fat percentage)<br>NHANES 2009	NI	NI
160	2017	de Lima	HIV+	Body composition;<br>Muscular strength/endurance;<br>Cardiorespiratory fitness;<br>Flexibility	Changes in: Body composition, Muscular strength/endurance, Cardiorespiratory fitness and Flexibility	Anthropometric: body mass, height, BMI, Triceps SF, Subscapular SF, Biceps SF, Suprailiac SF, SF ratio, abdominal girth, arm muscular circumference;<br>abdominal test, teste de isometria na barra;<br>Submaximal effort treadmill test: peak oxygen consumption;<br>Flexibility: teste de sit-to-reach	Not applied	NI	NI
161	2017	MacDonald	HIV+<br>HIV-	Body composition;<br>Muscular strength/endurance	Groups comparison	Anthropometric: body mass, height, BMI;<br>DXA: lean mass, body fat percentage;<br>Computed Tomography: muscle cross-sectional area;<br>Muscular strength/endurance: vertical jump	Z-scores	Structured questionnaire	Not applied
162	2017	Martins	HIV+	Body composition	Groups comparison	Anthropometric: body mass, height, BMI, arm	Not applied	Structured	PAQ-C score

			HIV-			relaxed circumference, waist circumference; Subscapular SF, Triceps SF, Abdominal SF, Calf SF, SF ratio; DXA: lean mass, body fat percentage;		questionnaire	
163	2017	Risti	HIV+	Body composition	Associations	X-ray: mandibular bone density	Not applied	NI	NI
164	2017	Sudjaritruk	HIV+	Body composition	Associations	Anthropometric: body mass, height, BMI; DXA: bone mass density	Growth curves (NCHS/WHO) (body mass, height) Thai reference values	Structured questionnaire	Not applied
165	2017	Sudjaritruk	HIV+	Body composition	Associations	Anthropometric: body mass, height, BMI; DXA: bone mass density	Growth curves (NCHS/WHO) (body mass, height) Thai reference values	Structured questionnaire	Not applied
166	2017	Ziegler	HIV+ HIV-	Body composition	Groups comparison	Anthropometric: body mass, height, BMI, waist circumference	Not applied	NI	NI
167	2018	Archary	HIV+	Body composition	Associations	Anthropometric: body mass, height, BMI, arm relaxed circumference; Fat-free mass (NR)	Not applied	NI	NI
168	2018	Cames	HIV+	Body composition	Associations	Anthropometric: body mass, height, BMI	Growth curves (NCHS/WHO)	NI	NI
169	2018	de Castro	HIV+	Body composition	Method validity	Anthropometric: body mass, height, BMI; BIA: body fat mass, body fat percentage, fat-free mass, lean mass, bone mass content; DXA: body fat mass, body fat percentage, fat-free mass, lean mass, bone mass content; Air displacement plethysmography: body fat mass, body fat percentage, fat-free mass	Not applied	NI	NI
170	2018	de Lima	HIV+ HIV-	Body composition	Groups comparison	Anthropometric: body mass, height, BMI, Triceps SF, Subscapular SF, Abdominal SF, Calf SF, arm relaxed circumference, waist circumference	Not applied	NI	NI
171	2018	de Lima	HIV+ HIV-	Body composition	Groups comparison	Anthropometric: body mass, height, BMI; DXA: body fat mass	Not applied	NI	NI
172	2018	Innes	HIV+	Body composition	Changes in Body composition	Anthropometric: body mass, height, BMI; Fat-free mass (NR)	Not applied	NI	NI
173	2018	Jacobson	HIV+ HIV-	Body composition	Groups comparison	Anthropometric: body mass, height; DXA: bone mass density, bone mass content, body fat mass, lean mass	Z-scores	NR	NR
174	2018	Puthanakit	HIV+	Body composition	Associations	Anthropometric: body mass, height, BMI DXA: bone mass density	(body mass, height) Thai reference values	NI	NI

175	2018	Ramteke	HIV+<br>HIV-	Body composition	Groups comparison	Anthropometric: body mass, height, BMI, arm relaxed circumference, hip circumference, waist circumference, waist-to-height ration, Biceps SF, Triceps SF, Subscapular SF, Suprailiac SF, Abdominal SF, Thigh SF, SF ratio, arm muscular circumference	Growth curves (NCHS/WHO)	NI	NI
176	2018	Rosales	HIV+	Body composition	Associations	Anthropometric: body mass, height, BMI, Triceps SF, Subscapular SF, waist circumference, arm relaxed circumference, calf circumference;<br>BIA: body fat mass, fat-free mass	Percentiles	NI	NI
177	2018	Sharma	HIV+<br>HIV-	Body composition	Groups comparison	Anthropometric: body mass, height, BMI, waist circumference, hip circumference, waist-to-height ration;<br>DXA: body fat mass, lean mass, body fat percentage	Not applied	NI	NI
178	2018	Shiau	HIV+<br>HIV-	Body composition	Groups comparison	Anthropometric: body mass, height, BMI;<br>DXA: bone mass content	BMI (NR)<br>(bone mass content) United States Bone Mineral Density in Childhood Study	NI	NI
179	2018	Strehlau	HIV+	Body composition	Associations	Anthropometric: body mass, height, arm relaxed circumference, waist circumference, hip circumference, thigh circumference, Biceps SF, Triceps SF, Subscapular SF, Suprailiac SF, Abdominal SF, Thigh SF, SF ratio;<br>DXA: bone mass content;<br>BIA: body fat percentage	Growth curves (NCHS/WHO)	NI	NI
180	2018	Torrejón	HIV+	Body composition	Associations	Anthropometric: body mass, height, BMI;<br>DXA: bone mass density	Growth curves (NCHS/WHO)<br>(bone mass density) NHANES III	NI	NI
181	2019	Alves Júnior	HIV+	Body composition	Associations	Anthropometric: body mass, height, BMI, Abdominal SF, Triceps SF, Subscapular SF, Calf SF, arm relaxed circumference, waist circumference, neck circumference; DXA: body fat mass;<br>Air displacement plethysmography: body fat mass	Not applied	Structured questionnaire	PA-C score
182	2019	Arpadi	HIV+<br>HIV-	Body composition	Groups comparison	Anthropometric: body mass, height;<br>Ultrasound: calcaneus stiffness	Growth curves (NCHS/WHO)	NI	NI
183	2019	de Lima	HIV+	Body composition<br>Cardiorespiratory fitness	Associations	Anthropometric: body mass, height;<br>Maximum effort cycle ergometer test : peak oxygen consumption;	(peak oxygen consumption)<br>Previous study	Accelerometer	WHO PA recommendations

						DXA: body fat percentage	(sample HIV-)		
184	2019	de Lima	HIV+	Body composition<br>Cardiorespiratory fitness	Method validity	Anthropometric: body mass, height, BMI;<br>Maximum effort cycle ergometer test : peak oxygen consumption	Not applied	NI	NI
185	2019	Dona	HIV+	Body composition	Associations	Anthropometric: body mass, height, BMI DXA: bone mass density	(bone mass density) WHO reference data (NR)	Structured questionnaire	NR
186	2019	Gregson	HIV+<br>HIV-	Body composition<br>Muscular strength/endurance	Groups comparison	Anthropometric: body mass, height;<br>Muscular strength/endurance: hand grip strength;<br>DXA: bone mass content, bone mass density, lean mass	Growth curves (NCHS/WHO)<br>(bone mass density, bone mass content) British Growth curves	NI	NI
187	2019	Malete	HIV+<br>HIV-	Body composition	Groups comparison	Anthropometric: body mass, height, BMI, waist circumference, hip circumference;	Growth curves (NCHS/WHO)	Structured questionnaire	Not applied
188	2019	Malete	HIV+<br>HIV-	Body composition<br>Muscular strength/endurance<br>Cardiorespiratory fitness	Groups comparison	Anthropometric: body mass, height, BMI;<br>Muscular strength/endurance: push-ups;<br>Cardiorespiratory fitness:<br>20 meters shuttle run test: total completed laps	Not applied	Structured questionnaire	Not applied
189	2019	Margossian	HIV+<br>HIV-	Body composition	Groups comparison	Anthropometric: body mass, height, BMI	Z-scores	Structured questionnaire	Minutes per day
190	2019	Marsico	HIV+<br>HIV-	Body composition	Groups comparison	Anthropometric: body mass, height, BMI	Percentiles	NI	NI
191	2019	Martins	HIV+	Body composition<br>Muscular strength/endurance<br>Cardiorespiratory fitness	Associations	Anthropometric: body mass, height, BMI;<br>DXA: body fat mass, lean mass;<br>Muscular strength/endurance: hand grip strength ;<br>Maximum effort cycle ergometer test: peak oxygen consumption	Z-scores	Accelerometer	Not applied
192	2019	de Souza	HIV+<br>HIV-	Body composition<br>Muscular strength/endurance	Groups comparison	Anthropometric: body mass, height, BMI, Triceps SF, sub SF, waist circumference, waist-to-height ratio;<br>Muscular strength/endurance: maximum expiratory pressure	NR	NI	NI
193	2020	Jacobson	HIV+	Body composition	Groups comparison	DXA: bone mass density	Z-scores	NI	NI
194	2020	Jacobson	HIV+<br>HIV-	Body composition	Groups comparison	Anthropometric: body mass, height, BMI;<br>DXA: bone mass density	(bone mass density) International Society for Clinical Densitometry	NI	NI
195	2020	Mahtab	HIV+<br>HIV-	Body composition	Groups comparison	Anthropometric: body mass, height, BMI, arm relaxed circumference, waist circumference, thigh circumference;	Growth curves (NCHS/WHO)	NI	NI

						Ultrasound: calcaneus stiffness			
196	2020	McHugh	HIV+	Body composition Cardiorespiratory fitness	Associations	Anthropometric: body mass, height, BMI; Cardiorespiratory fitness: incremental walking test	British Growth curves	NI	NI
197	2020	Naidoo	HIV+	Body composition Cardiorespiratory fitness	Associations	Anthropometric: body mass, height, BMI; Cardiorespiratory fitness: six minutes walking test	Growth curves (NCHS/WHO) (six minutes walking test) Previous study (sample HIV-) e American Thoracic Society	NI	NI
198	2020	Shiau	HIV+	Body composition	Associations	Anthropometric: body mass, height; DXA: bone mass content	Z-scores	NI	NI
199	2020	Shiau	HIV+ HIV-	Body composition	Groups comparison	Anthropometric: body mass, height, BMI; Computed Tomography: bone mass density	Growth curves (NCHS/WHO)	NI	NI
200	2021	Alves Júnior	HIV+	Body composition	Associations	Anthropometric: body mass, height, BMI, Abdominal SF, Triceps SF, Calf SF, SF ratio; DXA: body fat mass	Not applied	Accelerometer	Not applied
201	2021	Andrade	HIV+	Body composition	Associations	Anthropometric: body mass, height, BMI; DXA: bone mass density	Z-scores	NI	NI
202	2021	Bhise	HIV+	Body composition	Associations	Anthropometric: body mass, height, BMI; DXA: bone mass density	Indian Growth curves (bone mass density) Previous study (sample HIV-)	NI	NI
203	2021	Braithwaite	HIV+	Body composition	Associations	Anthropometric: body mass, height, BMI; DXA: bone mass content, bone mass density	Growth curves (NCHS/WHO) (bone mass content, bone mass density) NHANESS (NR)	NI	NI
204	2021	De Medeiros	HIV+	Body composition	Associations	Anthropometric: body mass, height, BMI; DXA: body fat percentage, lean mass, bone mass density	Not applied	Structured questionnaire	Not applied
205	2021	Dobe	HIV+	Body composition	Associations	Anthropometric: body mass, height, BMI, waist circumference, waist-to-height ratio	Growth curves (NCHS/WHO)	NI	NI
206	2021	Giacomet	HIV+	Body composition	Associations	Anthropometric: body mass, height, BMI, Triponderal mass index; DXA: body fat percentage	Not applied	NI	NI
207	2021	Jacobson	HIV+ HIV-	Body composition	Groups comparison	Anthropometric: body mass, height, BMI; DXA: bone mass content, bone mass density, lean mass	Not applied	NI	NI
208	2021	Lindsey	HIV+	Body composition	Changes in Body composition	DXA: bone mass density	Not applied	NI	NI

209	2021	Martins	HIV+	Body composition<br>Muscular strength/endurance	Associations	Anthropometric: body mass, height, BMI;<br>DXA: bone mass content, bone mass density, lean mass;<br>Muscular strength/endurance: hand grip strength	Not applied	Accelerometer	Not applied
210	2021	Martins	HIV+	Body composition	Associations	Anthropometric: body mass, height, BMI, Triceps SF, arm relaxed circumference;<br>BIA: body fat mass, lean mass	Growth curves (NCHS/WHO)<br>(Triceps SF, arm relaxed circumference)<br>Previous study (sample HIV-)	Structured questionnaire	Not applied
211	2021	Potterton	HIV+<br>HIV-	Body composition<br>Muscular strength/endurance	Groups comparison	Anthropometric: body mass, height, BMI;<br>Muscular strength/endurance: hand grip strength	Growth curves (NCHS/WHO)	NI	NI
212	2021	Rukuni	HIV+<br>HIV-	Body composition	Groups comparison	Anthropometric: body mass, height, BMI;<br>DXA: bone mass content, bone mass density	(BMI, bone mass density, bone mass content)<br>British Growth curves	Structured questionnaire	METs per week
213	2021	Shen	HIV+<br>HIV-	Body composition	Groups comparison	Anthropometric: body mass, height, BMI;<br>DXA: bone mass content, bone mass density	Growth curves (NCHS/WHO)<br>(bone mass density, bone mass content)<br>United States Bone Mineral Density in Childhood Study	Structured questionnaire	WHO PA recommendations
214	2021	Su	HIV+<br>HIV-	Body composition	Groups comparison	Anthropometric: body mass, height, BMI;<br>DXA: body fat mass, body fat percentage	Growth curves (NCHS/WHO)	Structured questionnaire	WHO PA recommendations
215	2021	Sudjaritruk	HIV+	Body composition	Associations	Anthropometric: body mass, height;<br>DXA: bone mass density	(body mass, height) Thai reference values<br>(bone mass density) Thai reference values	Structured questionnaire	Not applied
216	2022	Alves Júnior	HIV+	Body composition	Associations	Anthropometric: body mass, height, BMI Abdominal SF, Subscapular SF, Triceps SF, Calf SF, waist circumference, arm relaxed circumference, neck circumference	Not applied	Accelerometer	Not applied
217	2022	Chirindza	HIV+	Body composition<br>Muscular strength/endurance<br>Flexibility	Associations	Anthropometric: body mass, height, BMI, Subscapular SF, Triceps SF, arm relaxed circumference, SF ratio;<br>Muscular strength/endurance: abdominal test, hand grip strength, horizontal jump;	Growth curves (NCHS/WHO)<br>(arm relaxed circumference, SF ratios) Previous	Pedometer	WHO PA recommendations

						Flexibility: sit-to-reach	study (sample HIV-) (Muscular strength/endurance e Flexibility) AAPHER Youth Fitness Test, Fitnessgram, Alpha-fit		
218	2022	de Castro	HIV+	Body composition	Method validity	Anthropometric: body mass, height, BMI;<br>BIA: bone mass content, fat-free mass, lean mass, body fat mass, total body water;<br>DXA: bone mass content, fat-free mass, lean mass, body fat mass	Not applied	NI	NI
219	2022	Dirajlal-Fargo	HIV+	Body composition	Associations	Anthropometric: body mass, height, BMI;<br>DXA: body fat percentage	Growth curves (NCHS/WHO)	Structured questionnaire	Not applied
220	2022	Mahtab	HIV+<br>HIV-	Body composition	Groups comparison	Anthropometric: body mass, height, BMI, waist circumference, hip circumference, thigh circumference, arm relaxed circumference	Not applied	NI	NI
221	2022	Martins	HIV+	Body composition<br>Muscular strength/endurance	Associations	Anthropometric: body mass, height;<br>DXA: lean mass;<br>Muscular strength/endurance: hand grip strength	Not applied	NI	NI
222	2022	Martins	HIV+	Body composition<br>Muscular strength/endurance	Associations	Anthropometric: body mass, height;<br>DXA: lean mass, fat-free mass;<br>Muscular strength/endurance: hand grip strength	Not applied	Accelerometer	Not applied
223	2022	Melin	HIV+<br>HIV-	Body composition	Groups comparison	Anthropometric: height, body mass, BMI, waist circumference, hip circumference, waist to hip ratio	Growth curves (NCHS/WHO)	Structured questionnaire	Not applied
224	2022	Metgud	HIV+<br>HIV-	Body composition<br>Cardiorespiratory fitness<br>Muscular strength/endurance<br>Flexibility	Groups comparison	Anthropometric: body mass, height, BMI, waist circumference, hip circumference, waist-to-height ration;<br>Muscular strength/endurance: hand grip strength;<br>Flexibility: modified sit-to-reach test;<br>Cardiorespiratory fitness:<br>six minutes walking test; heart rate	Not applied	NI	NI
225	2022	Potterton	HIV+<br>HIV-	Body composition<br>Cardiorespiratory fitness	Groups comparison	Anthropometric: body mass, height, weight-for-age;<br>6 minutes walking test: total distance, maximum heart rate	Growth curves (NCHS/WHO)	NI	NI
226	2022	Rego	HIV+	Body composition<br>Muscular strength/endurance	Associations	Anthropometric: body mass, height, BMI;<br>Muscular strength/endurance: vertical jump	Z-scores	NI	NI
227	2022	Roberts	HIV+	Body composition	Method validity	Anthropometric: body mass, height, BMI;	Growth curves	NI	NI

			HIV-			DXA: bone mass density, bone mass content; Ultrasound: speed of sound, calcaneus stiffness	(NCHS/WHO) (bone mass density e bone mass content) United States Bone Mineral Density in Childhood Study		
228	2022	Rose	HIV+ HIV-	Body composition	Groups comparison	Anthropometric: body mass, height, BMI, waist circumference, hip circumference;	Growth curves (NCHS/WHO)	NI	NI
229	2022	Vargas	HIV+	Body composition	Associations	Anthropometric: body mass, height; DXA: bone mass density	Center of Disease Control and Prevention Growth Charts; NHANES III (bone mass density)	NI	NI
230	2022	Zanlorenci	HIV+	Body composition Muscular strength/endurance	Associations	Anthropometric: body mass, height, BMI; DXA: bone mass density, bone mass content, body fat percentage; Muscular strength/endurance: hand grip strength	Z-scores	Accelerometer	WHO PA recommendations
231	2022	Zanlorenci	HIV+	Body composition	Associations	Anthropometric: body mass, height, BMI, Triceps SF, Subscapular SF Abdominal SF, Calf SF; Air displacement plethysmography: body fat mass	Growth curves (NCHS/WHO)	Structured questionnaire	WHO PA recommendations
232	2023	Alves-Júnior	HIV+	Body composition	Associations	Anthropometric: body mass, height, abdominal SF, tricipital SF, subscapular SF, calf SF, body adiposity index, BMI, conicity index, arm relaxed circumference, waist circumference, neck circumference, waist-to-height ratio; DXA: fat mass, fat-free mass, fat mas percentage; Air displacement plethysmography: fat mass, fat-free mass, fat mas percentage	Percentiles	NI	NI
233	2023	Alves Júnior	HIV+	Body composition	Associations	Anthropometric: body mass, height; DXA: body fat mass	Terciles	Accelerometer	Not applied
234	2023	Comley-White	HIV+ HIV-	Body composition Cardiorespiratory fitness Muscular strength/endurance	Groups comparison	Anthropometric: body mass, height; Cardiorespiratory fitness: Shuttle run test; Muscular strength/endurance: standing broad jump	Growth curves (NCHS/WHO)	NI	NI
235	2023	Davies	HIV+ HIV-	Body composition	Groups comparison	Anthropometric: height; DXA: fat mass	Not applied	NI	NI
236	2023	Dirajlal-Fargo	HIV+	Body composition	Changes in Body	Anthropometric: body mass, height, BMI;	Growth curves	NI	NI

					composition	DXA: fat mass	(NCHS/WHO)		
237	2023	Franco-Oliva	HIV+<br>HIV-	Body composition<br>Muscular strength/endurance	Groups comparison	Anthropometric: body mass, height, waist circumference, hip circumference, thigh circumference, calf circumference, arm relaxed circumference, BMI;<br>BIA: fat-free mass, fat mass, soft lean mass, body water;<br>Muscular strength/endurance: hand grip strength	Growth curves (NCHS/WHO)	Structured questionnaire	WHO PA recommendations
238	2023	Gregson	HIV+<br>HIV-	Body composition<br>Muscular strength/endurance	Groups comparison	Anthropometric: body mass, height;<br>DXA: fat-free mass, fat mass;<br>Computed Tomography: muscle density;<br>Muscular strength/endurance: hand grip strength, long jump	Not applied	Structured questionnaire	METs per week
239	2023	Iheme	HIV+	Body composition	Associations	Anthropometric: body mass, height, BMI	Growth curves (NCHS/WHO)	NI	NI
240	2023	Maina	HIV+<br>HIV-	Body composition	Associations	Anthropometric: body mass, height, BMI	Growth curves (NCHS/WHO)	NI	NI
241	2023	Martins	HIV+	Body composition	Associations	Anthropometric: body mass, height, BMI;<br>DXA: lean soft tissue mass, fat mass, bone mass density, bone mineral content	Not applied	Accelerometer	Not applied
242	2023	Mukwasi-Kahari	HIV+<br>HIV-	Body composition	Groups comparison	Anthropometric: body mass, height, BMI<br>Computed Tomography: bone mass density;<br>DXA: body fat mass, lean mass	British Growth curves	Structured questionnaire	METs per week
243	2023	Natukunda	HIV+	Body composition	Prevalences	Anthropometric: body mass, height, BMI;<br>DXA: bone mass density	British Growth curves;<br>International Society for Clinical Densitometry	NI	NI
244	2023	Olibamoyo	HIV+<br>HIV-	Body composition	Groups comparison	Anthropometric: body mass, height, BM	Growth curves (NCHS/WHO)	NI	NI
245	2023	Rehman	HIV+<br>HIV-	Body composition	Groups comparison	Anthropometric: body mass, height, BMI;<br>DXA: fat mas, fat-free mass	British Growth curves	NI	NI
246	2023	Rukuni	HIV+<br>HIV-	Body composition	Groups comparison	Anthropometric: body mass, height;<br>DXA: bone mass density, bone mineral contet	Not applied	Structured questionnaire	METs per week

PA: physical activity; HIV or HIV-1: human immunodeficiency virus; HIV+: HIV-diagnosed; HIV-: without HIV infection diagnosis; SF: skinfold; NCHS: National Center for Health Statistics; WHO: World Health Organization; NI: not investigated; BIA: bioelectrical impedance analysis; DXA: dual energy X-ray absorptiometry; NR: not reported; BMI: body mass index; ACSM: American College of Sports Medicine; NHANES: National Health and Nutrition Examination Survey.

Supplementary Table S3. Physical activity investigation: methods/protocols, reference values and cut-points, aims and outcomes.

Protocol/test	Number of studies (n=50)	% of total studies (n=50)
Structured questionnaires	33	66.0%
Accelerometers	10	20.0%
Pedometers	2	4.0%
Not reported	5	10.0%
Reference values and cut-points	Number of studies (n=50)	% of total studies (n=50)
WHO physical activity recommendations	10	20.0%
Internal cut-point	12	24.0%
Days per week	2	4.0%
Percentiles	1	2.0%
Steps per day	1	2.0%
Z-score	1	2.0%
Minutes per day	1	2.0%
METs per week	4	8.0%
PAQ-C score	2	4.0%
Not reported	6	12.0%
Not applied	22	44.0%
Aims		
Groups comparisons (HIV-diagnosed and HIVV-non diagnosed)	21	
Associations with health-related physical fitness	12	
Model adjustments	8	
Sample description	6	
Match strategy	1	
Outcomes		
No difference related to physical activity level	11	
Low physical activity level to HIV-diagnosed group	10	
No association		
Body fat parameters	4	
Bone mass parameters	2	
Negative association		
Body fat parameters	4	
Positive association		
Bone mass parameters	2	
Fat-free mass	1	
Muscle strength/endurance	1	
Model adjustments		
No change in results after adjusted for physical activity level	8	
Changes in results after adjusted for physical activity level	0	

%: percentage: WHO: World Health Organization; MET: metabolic equivalent of task; PAQ-C: Physical Activity Questionary for Older Children.

Supplementary Table S4. References included in the scoping review

1. Miller TL, Awnetwant EL, Evans S *et al.* (1995) Gastrostomy tube supplementation for HIV-infected children. *Pediatrics* **96**, 696-702.
2. Saavedra JM, Henderson RA, Perman JA *et al.* (1995) LONGITUDINAL ASSESSMENT OF GROWTH IN CHILDREN BORN TO MOTHERS WITH HUMAN-IMMUNODEFICIENCY-VIRUS INFECTION. *ARCHIVES OF PEDIATRICS & ADOLESCENT MEDICINE* **149**, 497-502.
3. Arpadi SM, Wang J, Cuff PA *et al.* (1996) Application of bioimpedance analysis for estimating body composition in prepubertal children infected with human immunodeficiency virus type 1. *J Pediatr* **129**, 755-757.
4. Miller TL, Orav EJ, Colan SD, Lipshultz SE (1997) Nutritional status and cardiac mass and function in children infected with the human immunodeficiency virus. *AMERICAN JOURNAL OF CLINICAL NUTRITION* **66**, 660-664.
5. Arpadi SM, Horlick MNB, Wang J *et al.* (1998) Body composition in prepubertal children with human immunodeficiency virus type I infection. *Archives of Pediatrics and Adolescent Medicine* **152**, 688-693.
6. Henderson RA, Talusan K, Hutton N *et al.* (1998) Resting energy expenditure and body composition in children with HIV infection. *J Acquir Immune Defic Syndr Hum Retrovirol* **19**, 150-157.
7. Fontana M, Zuin G, Plebani A *et al.* (1999) Body composition in HIV-infected children: relations with disease progression and survival. *Am J Clin Nutr* **69**, 1282-1286.
8. Fox-Wheeler S, Heller L, Salata CM *et al.* (1999) Evaluation of the effects of oxandrolone on malnourished HIV-positive pediatric patients. *Pediatrics* **104**, e73.
9. Arpadi SM, Cuff PA, Arpadi SM *et al.* (2000) Growth velocity, fat-free mass and energy intake are inversely related to viral load in HIV-infected children. *Journal of Nutrition* **130**, 2498-2502.
10. Fiore P, Donelli E, Boni S *et al.* (2000) Nutritional status changes in HIV-infected children receiving combined antiretroviral therapy including protease inhibitors. *INTERNATIONAL JOURNAL OF ANTIMICROBIAL AGENTS* **16**, 365-369.
11. Heller L, Fox S, Hell KJ, Church JA (2000) Development of an instrument to assess nutritional risk factors for children infected with human immunodeficiency virus. *J Am Diet Assoc* **100**, 323-329.
12. Jansen AK, Lopez FA (2000) Avaliação da composição corporal por antropometria: crianças com síndrome da imunodeficiência humana. *Rev paul pediatr* **18**, 59-68.
13. Jaquet D, Lévine M, Ortega-Rodriguez E *et al.* (2000) Clinical and metabolic presentation of the lipodystrophic syndrome in HIV-infected children. *Aids* **14**, 2123-2128.
14. Keyser RE, Peralta L, Cade WT *et al.* (2000) Functional aerobic impairment in adolescents seropositive for HIV: a quasiexperimental analysis. *Arch Phys Med Rehabil* **81**, 1479-1484.
15. Missmer SA, Spiegelman D, Gorbach SL, Miller TL (2000) Predictors of change in the functional status of children with human immunodeficiency virus infection. *PEDIATRICS* **106**.
16. Arpadi SM, Cuff PA, Horlick M *et al.* (2001) Lipodystrophy in HIV-infected children is associated with high viral load and low CD4+ -lymphocyte count and CD4+ -lymphocyte percentage at baseline and use of protease inhibitors and stavudine. *J Acquir Immune Defic Syndr* **27**, 30-34.
17. Brambilla P, Bricalli D, Sala N *et al.* (2001) Highly active antiretroviral-treated HIV-infected children show fat distribution changes even in absence of lipodystrophy. *Aids* **15**, 2415-2422.
18. Dreimane D, Nielsen K, Deveikis A *et al.* (2001) Effect of protease inhibitors combined with standard antiretroviral therapy on linear growth and weight gain in human immunodeficiency virus type 1-infected children. *PEDIATRIC INFECTIOUS DISEASE JOURNAL* **20**, 315-316.
19. Ellis KJ, Shypailo RJ, Hardin DS *et al.* (2001) Z score prediction model for assessment of bone mineral content in pediatric diseases. *J Bone Miner Res* **16**, 1658-1664.
20. Melvin AJ, Lennon S, Mohan KM, Purnell JQ (2001) Metabolic abnormalities in HIV type 1-infected children treated and not treated with protease inhibitors. *AIDS Res Hum Retroviruses* **17**, 1117-1123.
21. Miller TL, Mawn BE, Orav EJ *et al.* (2001) The effect of protease inhibitor therapy on growth and body composition in human immunodeficiency virus type 1-infected children. *Pediatrics* **107**, E77.
22. Mora S, Sala N, Bricalli D *et al.* (2001) Bone mineral loss through increased bone turnover in HIV-infected children treated with highly active antiretroviral therapy. *Aids* **15**, 1823-1829.
23. O'Brien KO, Razavi M, Henderson RA *et al.* (2001) Bone mineral content in girls perinatally infected with HIV. *Am J Clin Nutr* **73**, 821-826.
24. Tan BM, Nelson Jr RP, James-Yarish M *et al.* (2001) Bone metabolism in children with human immunodeficiency virus infection receiving highly active anti-retroviral therapy including a protease inhibitor. *Journal of Pediatrics* **139**, 447-451.
25. Amaya RA, Kozinetz CA, McMeans A *et al.* (2002) Lipodystrophy syndrome in human immunodeficiency virus-infected children. *Pediatr Infect Dis J* **21**, 405-410.
26. Arpadi SM, Horlick M, Thornton J *et al.* (2002) Bone mineral content is lower in prepubertal HIV-infected children. *J Acquir Immune Defic Syndr* **29**, 450-454.
27. Cade WT, Peralta L, Keyser RE (2002) Aerobic capacity in late adolescents infected with HIV and controls. *Pediatr Rehabil* **5**, 161-169.
28. Cossarizza A, Pinti M, Moretti L *et al.* (2002) Mitochondrial functionality and mitochondrial DNA content in lymphocytes of vertically infected human immunodeficiency virus-positive children with highly active antiretroviral therapy-related lipodystrophy. *Journal of Infectious Diseases* **185**, 299-305.
29. Horlick M, Arpadi SM, Bethel J *et al.* (2002) Bioelectrical impedance analysis models for prediction of total body water and fat-free mass in healthy and HIV-infected children and adolescents. *American Journal of Clinical Nutrition* **76**, 991-999.
30. Nachman SA, Lindsey JC, Pelton S *et al.* (2002) Growth in human immunodeficiency virus-infected children receiving ritonavir-containing antiretroviral therapy. *ARCHIVES OF PEDIATRICS & ADOLESCENT MEDICINE* **156**, 497-503.
31. Rondanelli M, Caselli D, Aricò M *et al.* (2002) Insulin-like growth factor I (IGF-I) and IGF-binding protein 3 response to growth hormone is impaired in HIV-infected children. *AIDS RESEARCH AND HUMAN RETROVIRUSES* **18**, 331-339.

32. Verweel G, van Rossum AMC, Hartwig NG *et al.* (2002) Treatment with highly active antiretroviral therapy in human immunodeficiency virus type 1-infected children is associated with a sustained effect on growth. *PEDIATRICS* **109**.

33. Beregszàszi M, Jaquet D, Lévine M *et al.* (2003) Severe insulin resistance contrasting with mild anthropometric changes in the adipose tissue of HIV-infected children with lipohypertrophy. *Int J Obes Relat Metab Disord* **27**, 25-30.

34. Bitnun A, Sochett E, Babyn P *et al.* (2003) Serum lipids, glucose homeostasis and abdominal adipose tissue distribution in protease inhibitor-treated and naive HIV-infected children. *Aids* **17**, 1319-1327.

35. Bockhorst JL, Ksseiry I, Toye M *et al.* (2003) Evidence of human immunodeficiency virus-associated lipodystrophy syndrome in children treated with protease inhibitors. *Pediatr Infect Dis J* **22**, 463-465.

36. McComsey G, Bhumbra N, Rathore M, Alvarez A (2003) Impact of protease inhibitor substitution with efavirenz in HIV-infected children: Results of the first pediatric switch study. *PEDIATRICS* **111**.

37. Viganò A, Mora S, Brambilla P *et al.* (2003) Impaired growth hormone secretion correlates with visceral adiposity in highly active antiretroviral treated HIV-infected adolescents. *Aids* **17**, 1435-1441.

38. Viganò A, Mora S, Testolin C *et al.* (2003) Increased lipodystrophy is associated with increased exposure to highly active antiretroviral therapy in HIV-infected children. *J Acquir Immune Defic Syndr* **32**, 482-489.

39. Zamboni G, Antoniazzi F, Bertoldo F *et al.* (2003) Altered bone metabolism in children infected with human immunodeficiency virus. *Acta Paediatr* **92**, 12-16.

40. Ghaffari G, Passalacqua DJ, Caicedo JL *et al.* (2004) Two-year clinical and immune outcomes in human immunodeficiency virus-infected children who reconstitute CD4 T cells without control of viral replication after combination antiretroviral therapy. *Pediatrics* **114**, e604-611.

41. Hardin DS, Ellis KJ, Rice J, Doyle ME (2004) Protease inhibitor therapy improves protein catabolism in prepubertal children with HIV infection. *J Pediatr Endocrinol Metab* **17**, 321-325.

42. Mora S, Zamproni I, Beccio S *et al.* (2004) Longitudinal changes of bone mineral density and metabolism in antiretroviral-treated human immunodeficiency virus-infected children. *J Clin Endocrinol Metab* **89**, 24-28.

43. Panamonta O, Kosalaraksa P, Thinkhamrop B *et al.* (2004) Endocrine function in Thai children infected with human immunodeficiency virus. *JOURNAL OF PEDIATRIC ENDOCRINOLOGY & METABOLISM* **17**, 33-40.

44. Rojo Conejo P, Ramos Amador JT, García Piñar L *et al.* (2004) Decreased bone mineral density in HIV-infected children receiving highly active antiretroviral therapy. *An Pediatr (Barc)* **60**, 249-253.

45. Stagi S, Bindi G, Galluzzi F *et al.* (2004) Changed bone status in human immunodeficiency virus type 1 (HIV-1) perinatally infected children is related to low serum free IGF-1. *CLINICAL ENDOCRINOLOGY* **61**, 692-699.

46. Taylor P, Worrell C, Steinberg SM *et al.* (2004) Natural history of lipid abnormalities and fat redistribution among human immunodeficiency virus-infected children receiving long-term, protease inhibitor-containing, highly active antiretroviral therapy regimens. *Pediatrics* **114**, e235-242.

47. Thorne C (2004) Antiretroviral therapy, fat redistribution and hyperlipidaemia in HIV-infected children in Europe. *AIDS* **18**, 1443-1451.

48. Aldámiz-Echevarría L, Pocheville I, Sanjurjo P *et al.* (2005) Abnormalities in plasma fatty acid composition in human immunodeficiency virus-infected children treated with protease inhibitors. *ACTA PAEDIATRICA* **94**, 672-677.

49. Bitnun A, Sochett E, Dick PT *et al.* (2005) Insulin sensitivity and β-cell function in protease inhibitor-treated and -naive human immunodeficiency virus-infected children. *JOURNAL OF CLINICAL ENDOCRINOLOGY & METABOLISM* **90**, 168-174.

50. Giacomet V, Mora S, Martelli L *et al.* (2005) A 12-month treatment with tenofovir does not impair bone mineral accrual in HIV-infected children. *J Acquir Immune Defic Syndr* **40**, 448-450.

51. Hardin DS, Rice J, Doyle ME, Pavia A (2005) Growth hormone improves protein catabolism and growth in prepubertal children with HIV infection. *Clin Endocrinol (Oxf)* **63**, 259-262.

52. Hazra R, Gafni RI, Maldarelli F *et al.* (2005) Tenofovir disoproxil fumarate and an optimized background regimen of antiretroviral agents as salvage therapy for pediatric HIV infection. *Pediatrics* **116**, e846-e854.

53. Jacobson DL, Spiegelman D, Duggan C *et al.* (2005) Predictors of bone mineral density in human immunodeficiency virus-1 infected children. *J Pediatr Gastroenterol Nutr* **41**, 339-346.

54. Mora S, Zamproni I, Giacomet V *et al.* (2005) Analysis of bone mineral content in horizontally HIV-infected children naïve to antiretroviral treatment. *Calcified Tissue International* **76**, 336-340.

55. Pitukcheewanont P, Safani D, Church J, Gilsanz V (2005) Bone measures in HIV-1 infected children and adolescents: disparity between quantitative computed tomography and dual-energy X-ray absorptiometry measurements. *Osteoporos Int* **16**, 1393-1396.

56. Rosso R, Vignolo M, Parodi A *et al.* (2005) Bone quality in perinatally HIV-infected children: role of age, sex, growth, HIV infection, and antiretroviral therapy. *AIDS Res Hum Retroviruses* **21**, 927-932.

57. Viganò A, Mora S, Manzoni P *et al.* (2005) Effects of recombinant growth hormone on visceral fat accumulation: pilot study in human immunodeficiency virus-infected adolescents. *J Clin Endocrinol Metab* **90**, 4075-4080.

58. Barros C, Araújo T, Andrade E *et al.* (2006) Avaliação das variáveis de força muscular, agilidade e composição corporal em crianças vivendo com HIV/AIDS. *Rev bras ciênc mov* **14**, 47-54.

59. Ergun-Longmire B, Lin-Su K, Dunn AM *et al.* (2006) Effects of protease inhibitors on glucose tolerance, lipid metabolism, and body composition in children and adolescents infected with human immunodeficiency virus. *Endocr Pract* **12**, 514-521.

60. Gafni RI, Hazra R, Reynolds JC *et al.* (2006) Tenofovir disoproxil fumarate and an optimized background regimen of antiretroviral agents as salvage therapy: impact on bone mineral density in HIV-infected children. *Pediatrics* **118**, e711-718.

61. Gutierréz S, De León M, Cuñetti L *et al.* (2006) Dislipemia y lipodistrofia en niños uruguayos VIH positivos en tratamiento antirretroviral. *Rev méd Urug* **22**, 197-202.

62. Haroun D, Wells J, Lau C *et al.* (2006) Assessment of obesity status in outpatients from three disease states. *Acta Paediatrica, International Journal of Paediatrics* **95**, 970-974.

63. Hartman K, Verweel G, de Groot R, Hartwig NG (2006) Detection of lipoatrophy in human immunodeficiency virus-1-infected children treated with highly active antiretroviral therapy. *Pediatr Infect Dis J* **25**, 427-431.

64. Moscicki AB, Ellenberg JH, Murphy DA, Xu JH (2006) Associations among body composition, androgen levels, and human immunodeficiency virus status in adolescents. *JOURNAL OF ADOLESCENT HEALTH* **39**, 164-173.

65. Verkauskiene R, Dollfus C, Levine M *et al.* (2006) Serum adiponectin and leptin concentrations in HIV-infected children with fat redistribution syndrome. *Pediatr Res* **60**, 225-230.

66. Weidle PJ, Abrams EJ, Gvetadze R *et al.* (2006) A simplified weight-based method for pediatric drug dosing for zidovudine and didanosine in resource-limited settings. *Pediatr Infect Dis J* **25**, 59-64.

67. Chantry CJ, Frederick MM, Meyer WA, 3rd *et al.* (2007) Endocrine abnormalities and impaired growth in human immunodeficiency virus-infected children. *Pediatr Infect Dis J* **26**, 53-60.

68. Dzwonek AB, Novelli V, Schwenk A (2007) Serum leptin concentrations and fat redistribution in HIV-1-infected children on highly active antiretroviral therapy. *HIV Med* **8**, 433-438.

69. Ene L, Goetghebuer T, Hainaut M *et al.* (2007) Prevalence of lipodystrophy in HIV-infected children: a cross-sectional study. *EUROPEAN JOURNAL OF PEDIATRICS* **166**, 13-21.

70. Kim RJ, Carlow DC, Rutstein JH, Rutstein RM (2007) Hypoadiponectinemia, dyslipidemia, and impaired growth in children with HIV-associated facial lipoatrophy. *JOURNAL OF PEDIATRIC ENDOCRINOLOGY & METABOLISM* **20**, 65-74.

71. McComsey GA, O'Riordan M, Hazen SL *et al.* (2007) Increased carotid intima media thickness and cardiac biomarkers in HIV infected children. *Aids* **21**, 921-927.

72. Mora S, Zamproni I, Cafarelli L *et al.* (2007) Alterations in circulating osteoimmune factors may be responsible for high bone resorption rate in HIV-infected children and adolescents. *Aids* **21**, 1129-1135.

73. Papaevangelou V, Papassotiriou I, Vounatsou M *et al.* (2007) Changes in leptin serum levels in HIV-infected children receiving highly active antiretroviral therapy. *Scand J Clin Lab Invest* **67**, 291-296.

74. Tremeschin MH, Cervi MC, Camelo Júnior JS *et al.* (2007) Niacin nutritional status in HIV type 1-positive children: preliminary data. *J Pediatr Gastroenterol Nutr* **44**, 629-633.

75. Viganò A, Brambilla P, Cafarelli L *et al.* (2007) Normalization of fat accrual in lipoatrophic, HIV-infected children switched from stavudine to tenofovir and from protease inhibitor to efavirenz. *Antiviral Therapy* **12**, 297-302.

76. Viganò A, Zuccotti GV, Martelli L *et al.* (2007) Renal safety of tenofovir in HIV-infected children: A prospective, 96-week longitudinal study. *Clinical Drug Investigation* **27**, 573-581.

77. Chantry CJ, Hughes MD, Alvero C *et al.* (2008) Insulin-like growth factor-1 and lean body mass in HIV-infected children. *JAIDS-JOURNAL OF ACQUIRED IMMUNE DEFICIENCY SYNDROMES* **48**, 437-443.

78. Gonzalez-Tome MI, Amador JTR, Peña JM *et al.* (2008) Outcome of protease inhibitor substitution with nevirapine in HIV-1 infected children. *BMC Infectious Diseases* **8**.

79. Miller TL, Orav EJ, Lipshultz SE *et al.* (2008) Risk Factors for Cardiovascular Disease in Children Infected with Human Immunodeficiency Virus-1. *JOURNAL OF PEDIATRICS* **153**, 491-497.

80. Purdy JB, Gafni RI, Reynolds JC *et al.* (2008) Decreased bone mineral density with off-label use of tenofovir in children and adolescents infected with human immunodeficiency virus. *Journal of Pediatrics* **152**, 582-584.

81. Sharma TS, Kinnamon DD, Duggan C *et al.* (2008) Changes in macronutrient intake among HIV-infected children between 1995 and 2004. *AMERICAN JOURNAL OF CLINICAL NUTRITION* **88**, 384-391.

82. Spagnuolo MI, Bruzzese E, Vallone GF *et al.* (2008) Is resistin a link between highly active antiretroviral therapy and fat redistribution in HIV-infected children? *J Endocrinol Invest* **31**, 592-596.

83. Aldrovandi GM, Lindsey JC, Jacobson DL *et al.* (2009) Morphologic and metabolic abnormalities in vertically HIV-infected children and youth. *Aids* **23**, 661-672.

84. Arpadi SM, Bethel J, Horlick M *et al.* (2009) Longitudinal changes in regional fat content in HIV-infected children and adolescents. *Aids* **23**, 1501-1509.

85. López P, Caicedo Y, Rubiano LC *et al.* (2009) Alteraciones metabólicas con terapia antirretroviral altamente efectiva en niños positivos para VIH, Cali, Colombia. *Infectio* **13**, 283-292.

86. Mora S, Viganò A, Cafarelli L *et al.* (2009) Applicability of quantitative ultrasonography of the radius and tibia in HIV-infected children and adolescents. *J Acquir Immune Defic Syndr* **51**, 588-592.

87. Sarni ROS, Souza FISd, Battistini TRB *et al.* (2009) Lipodistrofia em crianças e adolescentes com síndrome da imunodeficiência adquirida e sua relação com a terapia antirretroviral empregada. *J pediatr (Rio J)* **85**, 329-334.

88. Viganò A, Brambilla P, Pattarino G *et al.* (2009) Long-Term Evaluation of Glucose Homeostasis in a Cohort of HAART-Treated HIV-Infected Children: A Longitudinal, Observational Cohort Study. *Clinical Drug Investigation* **29**, 101-109.

89. Cervia JS, Chantry CJ, Hughes MD *et al.* (2010) Associations of Proinflammatory Cytokine Levels With Lipid Profiles, Growth, and Body Composition in HIV-infected Children Initiating or Changing Antiretroviral Therapy. *PEDIATRIC INFECTIOUS DISEASE JOURNAL* **29**, 1118-1122.

90. Chantry CJ, Cervia JS, Hughes MD *et al.* (2010) Predictors of growth and body composition in HIV-infected children beginning or changing antiretroviral therapy. *HIV MEDICINE* **11**, 573-583.

91. Jacobson DL, Lindsey JC, Gordon CM *et al.* (2010) Total body and spinal bone mineral density across Tanner stage in perinatally HIV-infected and uninfected children and youth in PACTG 1045. *AIDS* **24**, 687-696.

92. Miller TL, Somarriba G, Kinnamon DD *et al.* (2010) The effect of a structured exercise program on nutrition and fitness outcomes in human immunodeficiency virus-infected children. *AIDS Res Hum Retroviruses* **26**, 313-319.

93. Miller TL, Somarriba G, Orav EJ *et al.* (2010) Biomarkers of vascular dysfunction in children infected with human immunodeficiency virus-1. *J Acquir Immune Defic Syndr* **55**, 182-188.

94. Stagi S, Galli L, Cecchi C *et al.* (2010) Final Height in Patients Perinatally Infected with the Human Immunodeficiency Virus. *HORMONE RESEARCH IN PAEDIATRICS* **74**, 165-171.

95. Viganò A, Zuccotti GV, Puzzovio M *et al.* (2010) Tenofovir disoproxil fumarate and bone mineral density: a 60-month longitudinal study in a cohort of HIV-infected youths. *Antivir Ther* **15**, 1053-1058.

96. Werner MLF, Pone MVdS, Fonseca VM, Chaves CRMdM (2010) Síndrome da lipodistrofia e fatores de risco cardiovasculares em crianças e adolescentes infectados pelo HIV/AIDS em uso de terapia antirretroviral de alta potência. *J pediatr (Rio J)* **86**, 27-32.

97. Zuccotti G, Viganò A, Gabiano C *et al.* (2010) Antiretroviral therapy and bone mineral measurements in HIV-infected youths. *Bone* **46**, 1633-1638.

98. Contri PV, Berchielli ÉM, Tremeschin MH *et al.* (2011) Nutritional status and lipid profile of HIV-positive children and adolescents using antiretroviral therapy. *Clinics* **66**, 997-1002.

99. da Silva QH, Pedro FL, Kirsten VR (2011) Body satisfaction and lipodystrophy characteristics in HIV/AIDS children and teenagers undergoing highly active antiretroviral therapy. *Revista Paulista de Pediatria* **29**, 357-363.

100. Dimock D, Thomas V, Cushing A *et al.* (2011) Longitudinal assessment of metabolic abnormalities in adolescents and young adults with HIV-infection acquired perinatally or in early childhood. *Metabolism* **60**, 874-880.

101. Geffner ME, Patel K, Miller TL *et al.* (2011) Factors Associated with Insulin Resistance among Children and Adolescents Perinatally Infected with HIV-1 in the Pediatric HIV/AIDS Cohort Study. *HORMONE RESEARCH IN PAEDIATRICS* **76**, 386-391.

102. Jacobson DL, Patel K, Siberry GK *et al.* (2011) Body fat distribution in perinatally HIV-infected and HIV-exposed but uninfected children in the era of highly active antiretroviral therapy: outcomes from the Pediatric HIV/AIDS Cohort Study. *AMERICAN JOURNAL OF CLINICAL NUTRITION* **94**, 1485-1495.

103. Mohd NM, Yeo J, Huang MS *et al.* (2011) Nutritional status of children living with HIV and receiving antiretroviral (ARV) medication in the Klang Valley, Malaysia. *Malays J Nutr* **17**, 19-30.

104. Morén C, Noguera-Julian A, Rovira N *et al.* (2011) Mitochondrial impact of human immunodeficiency virus and antiretrovirals on infected pediatric patients with or without lipodystrophy. *Pediatr Infect Dis J* **30**, 992-995.

105. Ramalho LCD, Gonçalves EM, de Carvalho WRG *et al.* (2011) Abnormalities in body composition and nutritional status in HIV-infected children and adolescents on antiretroviral therapy. *INTERNATIONAL JOURNAL OF STD & AIDS* **22**, 453-456.

106. Resino S, Micheloud D, Lorente R *et al.* (2011) Adipokine profiles and lipodystrophy in HIV-infected children during the first 4 years on highly active antiretroviral therapy. *HIV MEDICINE* **12**, 54-60.

107. Spoulou V, Kanaka-Gantenbein C, Bathrellou I *et al.* (2011) Monitoring of lipodystrophic and metabolic abnormalities in HIV-1 infected children on antiretroviral therapy. *Hormones (Athens)* **10**, 149-155.

108. Tremeschin MH, Sartorelli DS, Cervi MC *et al.* (2011) Nutritional assessment and lipid profile in HIV-infected children and adolescents treated with highly active antiretroviral therapy. *Rev Soc Bras Med Trop* **44**, 274-281.

109. Viganò A, Zuccotti GV, Cerini C *et al.* (2011) Lipodystrophy, insulin resistance, and adiponectin concentration in HIV-infected children and adolescents. *Curr HIV Res* **9**, 321-326.

110. Alam N, Cortina-Borja M, Goetghebuer T *et al.* (2012) Body Fat Abnormality in HIV-Infected Children and Adolescents Living in Europe: Prevalence and Risk Factors. *JAIDS-JOURNAL OF ACQUIRED IMMUNE DEFICIENCY SYNDROMES* **59**, 314-324.

111. Arpadi SM, McMahon DJ, Abrams EJ *et al.* (2012) Effect of supplementation with cholecalciferol and calcium on 2-y bone mass accrual in HIV-infected children and adolescents: a randomized clinical trial. *Am J Clin Nutr* **95**, 678-685.

112. Bhargav H, Huilgol V, Metri K *et al.* (2012) Evidence for extended age dependent maternal immunity in infected children: mother to child transmission of HIV infection and potential interventions including sulfatides of the human fetal adnexa and complementary or alternative medicines. *J Stem Cells* **7**, 127-153.

113. Innes S, Cotton MF, Haubrich R *et al.* (2012) High prevalence of lipoatrophy in pre-pubertal South African children on antiretroviral therapy: a cross-sectional study. *BMC PEDIATRICS* **12**.

114. Lindsey JC, Jacobson DL, Li H *et al.* (2012) Using cluster heat maps to investigate relationships between body composition and laboratory measurements in HIV-infected and HIV-uninfected children and young adults. *J Acquir Immune Defic Syndr* **59**, 325-328.

115. Miller TL, Borkowsky W, DiMeglio LA *et al.* (2012) Metabolic abnormalities and viral replication are associated with biomarkers of vascular dysfunction in HIV-infected children. *HIV MEDICINE* **13**, 264-275.

116. Negra MD, De Carvalho AP, De Aquino MZ *et al.* (2012) A randomized study of tenofovir disoproxil fumarate in treatment-experienced HIV-1 infected adolescents. *Pediatric infectious disease journal* **31**, 469-473.

117. Puthanakit T, Saksawad R, Bunupuradah T *et al.* (2012) Prevalence and risk factors of low bone mineral density among perinatally HIV-infected Thai adolescents receiving antiretroviral therapy. *J Acquir Immune Defic Syndr* **61**, 477-483.

118. Ramos E, Guttierrez-Teissoonniere S, Conde JG *et al.* (2012) Anaerobic power and muscle strength in human immunodeficiency virus-positive preadolescents. *PM & R: Journal of Injury, Function & Rehabilitation* **4**, 171-175.

119. Schtscherbyna A, Pinheiro MFMC, Mendonça LMC *et al.* (2012) Factors associated with low bone mineral density in a Brazilian cohort of vertically HIV-infected adolescents. *International Journal of Infectious Diseases* **16**, e872-e878.

120. Arpadi S, Shiau S, Strehlau R *et al.* (2013) Metabolic abnormalities and body composition of HIV-infected children on Lopinavir or Nevirapinebased antiretroviral therapy. *Archives of Disease in Childhood: Education and Practice Edition* **98**, 258-264.

121. Bunders MJ, Frinking O, Scherpbier HJ *et al.* (2013) Bone mineral density increases in HIV-infected children treated with long-term combination antiretroviral therapy. *Clin Infect Dis* **56**, 583-586.

122. Chokephaibulkit K, Saksawad R, Bunupuradah T *et al.* (2013) Prevalence of vitamin d deficiency among perinatally HIV-infected thai adolescents receiving antiretroviral therapy. *Pediatric Infectious Disease Journal* **32**, 1237-1239.

123. DiMeglio LA, Wang J, Siberry GK *et al.* (2013) Bone mineral density in children and adolescents with perinatal HIV infection. *Aids* **27**, 211-220.

124. Dos Santos FF, Pereira FB, da Silva CLO *et al.* (2013) Immunological and virological characteristics and performance in the variables flexibility and abdominal resistence strength of HIV/AIDS adolescents under highly active antirretroviral therapy. *Revista Brasileira de Medicina do Esporte* **19**, 40-43.

125. Fabiano V, Giacomet V, Viganò A *et al.* (2013) Long-term body composition and metabolic changes in HIV-infected children switched from stavudine to tenofovir and from protease inhibitors to efavirenz. *Eur J Pediatr* **172**, 1089-1096.

126. Innes S, Schulte-Kemna E, Cotton MF *et al.* (2013) Biceps skin-fold thickness may detect and predict early lipoatrophy in HIV-infected children. *Pediatr Infect Dis J* **32**, e254-262.

127. Lima LR, Silva RC, Giuliano Ide C *et al.* (2013) Bone mass in children and adolescents infected with human immunodeficiency virus. *J Pediatr (Rio J)* **89**, 91-99.

128. Macdonald HM, Chu J, Nettlefold L *et al.* (2013) Bone geometry and strength are adapted to muscle force in children and adolescents perinatally infected with HIV. *JOURNAL OF MUSCULOSKELETAL & NEURONAL INTERACTIONS* **13**, 53-65.

129. Palchetti CZ, Patin RV, Gouvêa AdFTB *et al.* (2013) Body composition and lipodystrophy in prepubertal HIV-infected children. *Braz j infect dis* **17**, 1-6.

130. Palchetti CZ, Patin RV, MacHado DM *et al.* (2013) Body composition in prepubertal, HIV-infected children: A comparison of bioelectrical impedance analysis and dual-energy X-ray absorptiometry. *Nutrition in Clinical Practice* **28**, 247-252.

131. Sharma TS, Jacobson DL, Anderson L *et al.* (2013) Short Communication: The Relationship Between Mitochondrial Dysfunction and Insulin Resistance in HIV-Infected Children Receiving Antiretroviral Therapy. *AIDS RESEARCH AND HUMAN RETROVIRUSES* **29**, 1211-1217.

132. Somarriba G, Lopez-Mitnik G, Ludwig DA *et al.* (2013) Physical fitness in children infected with the human immunodeficiency virus: associations with highly active antiretroviral therapy. *AIDS Res Hum Retroviruses* **29**, 112-120.

133. Agustinho A, Escobal N, Bologna R *et al.* (2014) Prevalencia de factores de riesgo de enfermedad cardiovascular en niños y adolescentes con infección por HIV. *Med infant* **21**, 301-309.

134. Dejkhamron P, Unachak K, Aurpibul L, Sirisanthana V (2014) Insulin resistance and lipid profiles in HIV-infected Thai children receiving lopinavir/ritonavir-based highly active antiretroviral therapy. *JOURNAL OF PEDIATRIC ENDOCRINOLOGY & METABOLISM* **27**, 403-412.

135. Foissac F, Meyzer C, Frange P *et al.* (2014) Determination of optimal vitamin D3 dosing regimens in HIV-infected paediatric patients using a population pharmacokinetic approach. *Br J Clin Pharmacol* **78**, 1113-1121.

136. Hillesheim E, Lima LRA, Silva RCR, Trindade EBSM (2014) Dietary intake and nutritional status of HIV-1-infected children and adolescents in Florianópolis, Brazil. *International Journal of STD and AIDS* **25**, 439-447.

137. Humphries C, Potterton J, Mudzi W (2014) A pilot study to investigate the muscle strength of children infected with HIV. *International Journal of Therapy and Rehabilitation* **21**, 19-24.

138. Musiime V, Cook A, Kayiwa J *et al.* (2014) Anthropometric measurements and lipid profiles to detect early lipodystrophy in antiretroviral therapy experienced HIV-infected children in the CHAPAS-3 trial. *ANTIVIRAL THERAPY* **19**, 269-276.

139. Theodoridou K, Margeli A, Spoulou V *et al.* (2014) Non-traditional adipokines in pediatric HIV-related lipodystrophy: a-FABP as a biomarker of central fat accumulation. *Scand J Clin Lab Invest* **74**, 67-73.

140. Vreeman RC, Nyandiko WM, Liechty EA *et al.* (2014) Impact of adherence and anthropometric characteristics on nevirapine pharmacokinetics and exposure among HIV-infected Kenyan children. *J Acquir Immune Defic Syndr* **67**, 277-286.

141. Aurpibul L, Cressey TR, Sricharoenchai S *et al.* (2015) Efficacy, safety and pharmacokinetics of tenofovir disoproxil fumarate in virologic-suppressed HIV-infected children using weight-band dosing. *Pediatr Infect Dis J* **34**, 392-397.

142. Cohen S, Innes S, Geelen SPM *et al.* (2015) Long-Term Changes of Subcutaneous Fat Mass in HIV-Infected Children on Antiretroviral Therapy: A Retrospective Analysis of Longitudinal Data from Two Pediatric HIV-Cohorts. *PLOS ONE* **10**.

143. Della Negra M, De Carvalho AP, De Aquino MZ *et al.* (2015) Long-term efficacy and safety of tenofovir disoproxil fumarate in HIV-1-infected adolescents failing antiretroviral therapy: the final results of study GS-US-104-0321. *Pediatr Infect Dis J* **34**, 398-405.

144. dos Reis LC, Rondó PHD, Marques HHD, Segri NJ (2015) Anthropometry and body composition of vertically HIV-infected children and adolescents under therapy with and without protease inhibitors. *PUBLIC HEALTH NUTRITION* **18**, 1255-1261.

145. Mora S, Puzzovio M, Giacomet V *et al.* (2015) Sclerostin and DKK-1: two important regulators of bone metabolism in HIV-infected youths. *Endocrine* **49**, 783-790.

146. Palchetti CZ, Szejnfeld VL, Succi RCdM *et al.* (2015) Impaired bone mineral accrual in prepubertal HIV-infected children: a cohort study. *Braz j infect dis* **19**, 623-630.

147. Swetha GK, Hemalatha R, Prasad UV *et al.* (2015) Health & nutritional status of HIV infected children in Hyderabad, India. *Indian J Med Res* **141**, 46-54.

148. Arpadi SM, Shiau S, Strehlau R *et al.* (2016) Efavirenz is associated with higher bone mass in South African children with HIV. *Aids* **30**, 2459-2467.

149. Gaur AH, Kizito H, Prasitsueubsai W *et al.* (2016) Safety, efficacy, and pharmacokinetics of a single-tablet regimen containing elvitegravir, cobicistat, emtricitabine, and tenofovir alafenamide in treatment-naive, HIV-infected adolescents: a single-arm, open-label trial. *The Lancet HIV* **3**, e561-e568.

150. Lima LRAD, Krug RDR, Silva RCRD *et al.* (2016) Prediction of Areal Bone Mineral Density and Bone Mineral Content in Children and Adolescents Living With HIV Based on Anthropometric Variables. *Journal of Clinical Densitometry* **19**, 457-464.

151. Sonego M, Sagrado MJ, Escobar G *et al.* (2016) Dyslipidemia, Diet and Physical Exercise in Children on Treatment With Antiretroviral Medication in El Salvador: A Cross-sectional Study. *PEDIATRIC INFECTIOUS DISEASE JOURNAL* **35**, 1111-1116.

152. Sudjaritruk T, Bunupuradah T, Aurpibul L *et al.* (2016) Hypovitaminosis D and hyperparathyroidism: effects on bone turnover and bone mineral density among perinatally HIV-infected adolescents. *AIDS* **30**, 1059-1067.

153. Wong M, Shiau S, Yin MT *et al.* (2016) Decreased Vigorous Physical Activity in School-Aged Children with Human Immunodeficiency Virus in Johannesburg, South Africa. *JOURNAL OF PEDIATRICS* **172**, 103-109.

154. Carmo FB, Terreri MT, Succi RCM *et al.* (2017) Bone mineral density and vitamin D concentration: the challenges in taking care of children and adolescents infected with HIV. *Braz J Infect Dis* **21**, 270-275.

155. de Lima LRA, Santos Silva DA, Samara da Silva K *et al.* (2017) Aerobic Fitness and Moderate to Vigorous Physical Activity in Children and Adolescents Living with HIV. *Pediatric Exercise Science* **29**, 377-387.

156. Giacomet V, Maruca K, Ambrosi A *et al.* (2017) A 10−year follow−up of bone mineral density in HIV−infected youths receiving tenofovir disoproxil fumarate. *International Journal of Antimicrobial Agents* **50**, 365-370.

157. Jacobson DL, Stephensen CB, Miller TL *et al.* (2017) Associations of Low Vitamin D and Elevated Parathyroid Hormone Concentrations With Bone Mineral Density in Perinatally HIV-Infected Children. *J Acquir Immune Defic Syndr* **76**, 33-42.

158. Jiménez B, Sainz T, Díaz L *et al.* (2017) Low Bone Mineral Density in Vertically HIV-infected Children and Adolescents: Risk Factors and the Role of T-cell Activation and Senescence. *Pediatric Infectious Disease Journal* **36**, 578-583.

159. Lima LRAd, Back IdC, Beck CC, Caramelli B (2017) Exercise Improves Cardiovascular Risk Factors, Fitness, and Quality Of Life in Hiv+ Children and Adolescents: Pilot Study. *International Journal of Cardiovascular Sciences* **30**, 171-176.

160. Lima LRAD, Martins PC, Junior CASA *et al.* (2017) Are traditional body fat equations and anthropometry valid to estimate body fat in children and adolescents living with HIV? *Brazilian Journal of Infectious Diseases* **21**, 448-456.

161. Macdonald H, Nettlefold L, Maan EJ *et al.* (2017) Muscle power in children, youth and young adults who acquired HIV perinatally. *J Musculoskelet Neuronal Interact* **17**, 27-37.

162. Martins PC, De Lima LRA, Teixeira DM *et al.* (2017) Physical activity and body fat in adolescents living with HIV: A comparative study. *Revista Paulista de Pediatria* **35**, 69-77.

163. Risti Saptarini P, Riyanti E, Sufiawati I, Sasmita IS (2017) Level vitamin D, calcium serum and mandibular bone density in HIV/AIDS children. *Journal of International Dental and Medical Research* **10**, 313-317.

164. Sudjaritruk T, Bunupuradah T, Aurpibul L *et al.* (2017) Adverse bone health and abnormal bone turnover among perinatally HIV-infected Asian adolescents with virological suppression. *HIV Medicine* **18**, 235-244.

165. Sudjaritruk T, Bunupuradah T, Aurpibul L *et al.* (2017) Impact of tenofovir disoproxil fumarate on bone metabolism and bone mass among perinatally HIV-infected Asian adolescents. *ANTIVIRAL THERAPY* **22**, 471-479.

166. Ziegler TR, Judd SE, Ruff JH *et al.* (2017) Amino Acid Concentrations in HIV-Infected Youth Compared to Healthy Controls and Associations with CD4 Counts and Inflammation. *AIDS Res Hum Retroviruses* **33**, 681-689.

167. Archary M, McLlleron H, Bobat R *et al.* (2018) Population Pharmacokinetics of Lopinavir in Severely Malnourished HIV-infected Children and the Effect on Treatment Outcomes. *Pediatric Infectious Disease Journal* **37**, 349-355.

168. Cames C, Pascal L, Ba A *et al.* (2018) Low prevalence of lipodystrophy in HIV-infected Senegalese children on long-term antiretroviral treatment: the ANRS 12279 MAGGSEN Pediatric Cohort Study. *BMC INFECTIOUS DISEASES* **18**.

169. de Castro JAC, de Lima LRA, Silva DAS (2018) Accuracy of octa-polar bioelectrical impedance analysis for the assessment of total and appendicular body composition in children and adolescents with HIV: comparison with dual energy X-ray absorptiometry and air displacement plethysmography. *Journal of Human Nutrition & Dietetics* **31**, 276-285.

170. de Lima LRA, Monteiro Teixeira D, Custódio Martins P *et al.* (2018) Body image and anthropometric indicators in adolescents living with HIV. *Brazilian Journal of Kineanthropometry & Human Performance* **20**, 53-63.

171. De Lima LRA, Petroski EL, Moreno YMF *et al.* (2018) Dyslipidemia, chronic inflammation, and subclinical atherosclerosis in children and adolescents infected with HIV: The PositHIVe Health Study. *PLoS ONE* **13**.

172. Innes S, van der Laan L, Anderson PL *et al.* (2018) Can We Improve Stavudine's Safety Profile in Children? Pharmacokinetics of Intracellular Stavudine Triphosphate with Reduced Dosing. *Antimicrob Agents Chemother* **62**.

173. Jacobson DL, Lindsey JC, Coull BA *et al.* (2018) The Association of Fat and Lean Tissue with Whole Body and Spine Bone Mineral Density Is Modified by HIV Status and Sex in Children and Youth. *Pediatric Infectious Disease Journal* **37**, 71-77.

174. Puthanakit T, Wittawatmongkol O, Poomlek V *et al.* (2018) Effect of calcium and vitamin D supplementation on bone mineral accrual among HIV-infected Thai adolescents with low bone mineral density. *J Virus Erad* **4**, 6-11.

175. Ramteke SM, Shiau S, Foca M *et al.* (2018) Patterns of Growth, Body Composition, and Lipid Profiles in a South African Cohort of Human Immunodeficiency Virus-Infected and Uninfected Children: A Cross-Sectional Study. *JOURNAL OF THE PEDIATRIC INFECTIOUS DISEASES SOCIETY* **7**, 143-150.

176. Rosales JGV, Juárez Moya A, García Samano VM, Solórzano Santos F (2018) Lipodystrophy syndrome in HIV-1 infected pediatric patients, under highly effective antiretroviral therapy (HAART), attending at a high specialty hospital. *Enfermedades Infecciosas y Microbiologia* **38**, 123-130.

177. Sharma TS, Somarriba G, Arheart KL *et al.* (2018) Longitudinal Changes in Body Composition by Dual-energy Radiograph Absorptiometry Among Perinatally HIV-infected and HIV-uninfected Youth: Increased Risk of Adiposity Among HIV-infected Female Youth. *Pediatr Infect Dis J* **37**, 1002-1007.

178. Shiau S, Yin MT, Strehlau R *et al.* (2018) Decreased bone turnover in HIV-infected children on antiretroviral therapy. *Arch Osteoporos* **13**, 40.

179. Strehlau R, Shiau S, Arpadi S *et al.* (2018) Substituting Abacavir for Stavudine in Children Who Are Virally Suppressed Without Lipodystrophy: Randomized Clinical Trial in Johannesburg, South Africa. *J Pediatric Infect Dis Soc* **7**, e70-e77.

180. Torrejón C, Galaz MI, Vizueta E *et al.* (2018) Evaluation of bone mineral density in children with vertical infection by HIV. *Rev Chilena Infectol* **35**, 634-641.

181. Alves Junior CAS, de Lima LRA, de Souza MC, Silva DAS (2019) Anthropometric measures associated with fat mass estimation in children and adolescents with HIV. *Appl Physiol Nutr Metab* **44**, 493-498.

182. Arpadi SM, Thurman CB, Patel F *et al.* (2019) Bone Quality Measured Using Calcaneal Quantitative Ultrasonography Is Reduced Among Children with HIV in Johannesburg, South Africa. *J Pediatr* **215**, 267-271.e262.

183. de Lima LRA, Silva DAS, do Nascimento Salvador PC *et al.* (2019) Prediction of peak V˙ O(2) in Children and Adolescents With HIV From an Incremental Cycle Ergometer Test. *Res Q Exerc Sport* **90**, 163-171.

184. de Lima LRA, Silva DAS, Petroski EL *et al.* (2019) Aerobic fitness and physical activity are inversely associated with body fat, dyslipidemia and inflammatory mediators in children and adolescents living with HIV. *Journal of Sports Sciences* **37**, 50-58.

185. Donà D, Mozzo E, Luise D *et al.* (2019) Impact of HIV-1 Infection and Antiretroviral Therapy on Bone Homeostasis and Mineral Density in Vertically Infected Patients. *Journal of Osteoporosis* **2019**.

186. Gregson CL, Hartley A, Majonga E *et al.* (2019) Older age at initiation of antiretroviral therapy predicts low bone mineral density in children with perinatally-infected HIV in Zimbabwe. *Bone* **125**, 96-102.

187. Malete L, Etnier JL, Tladi DM *et al.* (2019) Predicting cognitive performance from physical activity and fitness in adolescents and young adults in Botswana relative to HIV status. *Sci Rep* **9**, 19583.

188. Malete L, Tladi DM, Etnier JL *et al.* (2019) Examining psychosocial correlates of physical activity and sedentary behavior in youth with and without HIV. *PLoS One* **14**, e0225890.

189. Margossian R, Williams PL, Yu W *et al.* (2019) Markers of Bone Mineral Metabolism and Cardiac Structure and Function in Perinatally HIV-Infected and HIV-Exposed but Uninfected Children and Adolescents. *J Acquir Immune Defic Syndr* **81**, 238-246.

190. Marsico F, Lo Vecchio A, Paolillo S *et al.* (2019) Left Ventricular Function, Epicardial Adipose Tissue, and Carotid Intima-Media Thickness in Children and Adolescents With Vertical HIV Infection. *J Acquir Immune Defic Syndr* **82**, 462-467.

191. Martins PC, Lima LRA, Silva AM *et al.* (2019) Phase angle is associated with the physical fitness of HIV-infected children and adolescents. *Scandinavian Journal of Medicine & Science in Sports* **29**, 1006-1012.

192. Souza LAAd, Nogueira MM, Vianna TdS *et al.* (2019) Influence of body composition on the respiratory muscle strength of children exposed to antiretroviral therapy forhuman immunodeficiency virus. *Mundo saúde (Impr)* **43**, [955-975].

193. Jacobson DL, Lindsey JC, Gordon C *et al.* (2020) Alendronate Improves Bone Mineral Density in Children and Adolescents Perinatally Infected With Human Immunodeficiency Virus With Low Bone Mineral Density for Age. *Clinical infectious diseases* **71**, 1281-1288.

194. Jacobson DL, Yu W, Hazra R *et al.* (2020) Fractures in children and adolescents living with perinatally acquired HIV. *Bone* **139**, 115515.

195. Mahtab S, Scott C, Asafu-Agyei NAA *et al.* (2020) Prevalence and predictors of bone health among perinatally HIV-infected adolescents. *Aids* **34**, 2061-2070.

196. McHugh G, Rehman AM, Simms V *et al.* (2020) Chronic lung disease in children and adolescents with HIV: a case–control study. *Tropical Medicine and International Health* **25**, 590-599.

197. Naidoo CN, Benjamin-Damons N, Strehlau R, Potterton J (2020) The effects of a home exercise programme on the exercise endurance of children infected with HIV. *SOUTH AFRICAN JOURNAL OF CHILD HEALTH* **14**, 174-179.

198. Shiau S, Yin MT, Strehlau R *et al.* (2020) Deficits in Bone Architecture and Strength in Children Living With HIV on Antiretroviral Therapy. *JAIDS-JOURNAL OF ACQUIRED IMMUNE DEFICIENCY SYNDROMES* **84**, 101-106.

199. Shiau S, Yin MT, Strehlau R *et al.* (2020) Bone turnover markers in children living with HIV remaining on ritonavir-boosted lopinavir or switching to efavirenz. *Bone* **138**, 115500.

200. Alves Junior CAS, Martins PC, de Andrade Gonçalves EC *et al.* (2021) Association Between Body Fat Distribution Assessed by Different Techniques and Body Image Perception in HIV-Infected Children and Adolescents. *J Pediatr Nurs* **60**, e74-e79.

201. Andrade LBd, Nogueira TF, Vargas DM (2021) Height adjustment reduces occurrence of low bone mineral density in children and adolescents with HIV. *Rev Assoc Med Bras (1992)* **67**, 1240-1245.

202. Bhise S, Jain A, Savardekar L *et al.* (2021) Bone health in HIV-infected children on antiretroviral therapy: An Indian study. *Indian Journal of Sexually Transmitted Diseases and AIDS* **42**, 138-143.

203. Braithwaite K, McPherson TD, Shen YH *et al.* (2021) Bone outcomes in virally suppressed youth with HIV switching to tenofovir disoproxil fumarate. *SOUTHERN AFRICAN JOURNAL OF HIV MEDICINE* **22**.

204. de Medeiros RCdSC, da Silva TAL, de Oliveira ALV *et al.* (2021) Influence of Healthy Habits Counseling on Biochemical and Metabolic Parameters in Children and Adolescents with HIV: Longitudinal Study. *Nutrients* **13**, 3237-3237.

205. Dobe IS, Mocumbi AO, Majid N *et al.* (2021) Earlier antiretroviral initiation is independently associated with better arterial stiffness in children living with perinatally acquired hiv with sustained viral suppression in mozambique. *Southern African Journal of HIV Medicine* **22**.

206. Giacomet V, Lazzarin S, Manzo A *et al.* (2021) Body Fat Distribution and Metabolic Changes in a Cohort of Adolescents Living With HIV Switched to an Antiretroviral Regimen Containing Dolutegravir. *Pediatr Infect Dis J* **40**, 457-459.

207. Jacobson D, Liu JZ, Lindsey JC *et al.* (2021) Immune Markers and Their Association with Bone Density in Children, Adolescents, and Young Adults with Perinatally Acquired HIV. *AIDS Res Hum Retroviruses* **37**, 122-129.

208. Lindsey JC, Jacobson DL, Spiegel HM *et al.* (2021) Safety and Efficacy of 48 and 96 Weeks of Alendronate in Children and Adolescents With Perinatal Human Immunodeficiency Virus Infection and Low Bone Mineral Density for Age. *Clin Infect Dis* **72**, 1059-1063.

209. Martins IDC, Asseiceira I, Policarpo S *et al.* (2021) Nutritional status, physical activity and quality of life in children and adolescents with human immunodeficiency virus infection. *Portuguese Journal of Pediatrics* **52**, 98-106.

210. Martins PC, de Lima LRA, de Lima TR *et al.* (2021) Association between handgrip strength and bone mass parameters in HIV-infected children and adolescents. A cross-sectional study. *Sao Paulo Med J* **139**, 405-411.

211. Potterton J, Strehlau R, Shiau S *et al.* (2021) Muscle strength in young children perinatally infected with HIV who were initiated on antiretroviral therapy early. *SOUTH AFRICAN JOURNAL OF CHILD HEALTH* **15**, 107-111.

212. Rukuni R, Rehman AM, Mukwasi-Kahari C *et al.* (2021) Effect of HIV infection on growth and bone density in peripubertal children in the era of antiretroviral therapy: a cross-sectional study in Zimbabwe. *Lancet Child Adolesc Health* **5**, 569-581.

213. Shen YH, Shiau S, Strehlau R *et al.* (2021) Persistently lower bone mass and bone turnover among South African children living with well controlled HIV. *AIDS* **35**, 2137-2147.

214. Su JW, Shiau S, Arpadi SM *et al.* (2021) Switch to Efavirenz Attenuates Lipoatrophy in Girls With Perinatal HIV. *JOURNAL OF PEDIATRIC GASTROENTEROLOGY AND NUTRITION* **72**, E15-E20.

215. Sudjaritruk T, Bunupuradah T, Aurpibul L *et al.* (2021) Impact of Vitamin D and Calcium Supplementation on Bone Mineral Density and Bone Metabolism Among Thai Adolescents With Perinatally Acquired Human Immunodeficiency Virus (HIV) Infection: A Randomized Clinical Trial. *Clinical Infectious Diseases* **73**, 1555-1564.

216. Alves Junior CAS, Martins PC, Lima LRAd, Silva DAS (2022) What anthropometric indicators are associated with insulin resistance? Cross-sectional study on children and adolescents with diagnosed human immunodeficiency virus. *Sao Paulo Medical Journal* **140**, 94-100.

217. Chirindza N, Leach L, Mangona L *et al.* (2022) Body composition, physical fitness and physical activity in Mozambican children and adolescents living with HIV. *PLoS One* **17**, e0275963.

218. de Castro JAC, de Lima LRA, Silva DAS (2022) Bone Mineral Content Prediction by Bioelectrical Impedance Analysis in Children and Adolescents Diagnosed with HIV Infection: Comparison with Dual Energy X-ray Absorptiometry: A Cross-Sectional Study. *APPLIED SCIENCES-BASEL* **12**.

219. Dirajlal-Fargo S, Jacobson DL, Yu W *et al.* (2022) Gut Dysfunction Markers Are Associated With Body Composition in Youth Living With Perinatally Acquired Human Immunodeficiency Virus. *Clin Infect Dis* **75**, 945-952.

220. Mahtab S, Jao J, Myer L *et al.* (2022) The association between mental health and metabolic outcomes in youth living with perinatally acquired HIV in the Cape Town Adolescent Antiretroviral Cohort. *AIDS Care* **34**, 1151-1158.

221. Martins PC, Alves Junior CAS, Lima LRAD *et al.* (2022) Does antiretroviral therapy change the relationship between body composition and muscle strength in children and adolescents diagnosed with HIV? *HIV Research and Clinical Practice* **23**, 22-27.

222. Martins PC, Souza Alves Junior CA, Augustemak de Lima LR *et al.* (2022) Muscle mass indicators as fat-free mass and lean soft tissue mass are associated with handgrip strength in HIV-diagnosed children and adolescents. *J Bodyw Mov Ther* **30**, 76-81.

223. Mellin J, Le Prevost M, Kenny J *et al.* (2022) Arterial Stiffness in a Cohort of Young People Living With Perinatal HIV and HIV Negative Young People in England. *Frontiers in Cardiovascular Medicine* **9**.

224. Metgud DC, Chheda RJ (2022) Muscle strength, flexibility and cardiorespiratory endurance in children with human immunodeficiency virus on antiretroviral therapy: A case control study. *Sri Lanka Journal of Child Health* **51**, 560-564.

225. Potterton J, Strehlau R, Shiau S *et al.* (2022) Evaluation of submaximal endurance in young children living with HIV. *South African Journal of Physiotherapy* **78**, 1-6.

226. Rego CV, Potterton JL (2022) Motor function, muscle strength and health-related quality of life of children perinatally infected with HIV. *S Afr J Physiother* **78**, 1812.

227. Roberts JA, Shen Y, Strehlau R *et al.* (2022) Comparison of quantitative ultrasonography and dual X-ray absorptiometry for bone status assessment in South African children living with HIV. *PLoS One* **17**, e0276290.

228. Rose PC, Nel ED, Cotton MF *et al.* (2022) Prevalence and Risk Factors for Hepatic Steatosis in Children With Perinatal HIV on Early Antiretroviral Therapy Compared to HIV-Exposed Uninfected and HIV-Unexposed Children. *Front Pediatr* **10**, 893579.

229. Vargas DM, Daniela de Oliveira P, José Carlos Pereira G *et al.* (2022) Massa óssea em crianças e adolescentes com infecção vertical pelo HIV: uma série de casos. *Rev Assoc Méd Rio Gd do Sul* **66**, 01022105-01022105.

230. Zanlorenci S, de Souza MC, Martins CR *et al.* (2022) Factors Correlated with Body Image Dissatisfaction in Children and Adolescents Diagnosed with HIV: A Cross-Sectional Study. *Int J Environ Res Public Health* **19**.

231. Zanlorenci S, Martins PC, Junior CASA *et al.* (2022) Association between bone mineral density and content and physical growth parameters among children and adolescents diagnosed with HIV: a cross-sectional study. *Sao Paulo Medical Journal* **140**, 682-690.

232. Alves CAS, Augustemak De Lima LR, Franco Moreno YM, Santos Silva DA (2023) Anthropometric indicators as discriminators of high body fat in children and adolescents with HIV: comparison with reference methods. *Minerva Pediatrics* **75**, 828-835.

233. Alves Junior CAS, Martins PC, Gonçalves ECA *et al.* (2023) Association between lipid and glycemic profile and total body and trunk fat in children and adolescents diagnosed with HIV. *Clin Nutr ESPEN* **53**, 7-12.

234. Comley-White N, Ntsiea V, Potterton J (2023) Physical functioning in adolescents with perinatal HIV. *AIDS Care*, 1-10.

235. Davies C, Vaida F, Otwombe K *et al.* (2023) Longitudinal comparison of insulin resistance and dyslipidemia in children with and without perinatal HIV infection in South Africa. *Aids* **37**, 523-533.

236. Dirajlal-Fargo S, Jacobson DL, Yu W *et al.* (2023) Longitudinal changes in body fat and metabolic complications in young people with perinatally acquired HIV. *HIV Med*.

237. Franco-Oliva A, Pinzón-Navarro BA, Martínez-Soto-Holguín MC *et al.* (2023) High resting energy expenditure, less fat-free mass, and less muscle strength in HIV-infected children: a matched, cross-sectional study. *Front Nutr* **10**, 1220013.

238. Gregson CL, Rehman AM, Rukuni R *et al.* (2023) Perinatal HIV infection is associated with deficits in muscle function in children and adolescents: a cross-sectional study in Zimbabwe. *Aids*.

239. Iheme GO (2023) Health-related quality of life and nutritional status of people living with HIV/AIDS in South-East Nigeria; a facility-based study. *Human Nutrition and Metabolism* **32**.

240. Maina R, He J, Abubakar A *et al.* (2023) The effects of height-for-age and HIV on cognitive development of school-aged children in Nairobi, Kenya: a structural equation modelling analysis. *Front Public Health* **11**, 1171851.

241. Martins PC, de Lima LRA, Silva AM, Silva DAS (2023) Association between Phase Angle and Body Composition of Children and Adolescents Diagnosed with HIV Infection. *Children (Basel)* **10**.

242. Mukwasi-Kahari C, Rehman AM, Ó Breasail M *et al.* (2023) Impaired Bone Architecture in Peripubertal Children With HIV, Despite Treatment With Antiretroviral Therapy: A Cross-Sectional Study From Zimbabwe. *Journal of Bone & Mineral Research* **38**, 248-260.

243. Natukunda E, Szubert A, Otike C *et al.* (2023) Bone mineral density among children living with HIV failing first-line anti-retroviral therapy in Uganda: A sub-study of the CHAPAS-4 trial. *PLoS One* **18**, e0288877.

244. Olibamoyo OB, Akintan PE, Adeniyi OF, Soriyan OO (2023) Serum vitamin E levels in children with human immunodeficiency virus infection in Lagos Nigeria. *Egyptian Pediatric Association Gazette* **71**.

245. Rehman AM, Sekitoleko I, Rukuni R *et al.* (2023) Growth Profiles of Children and Adolescents Living with and without Perinatal HIV Infection in Southern Africa: A Secondary Analysis of Cohort Data. *Nutrients* **15**.

246. Rukuni R, Simms V, Rehman AM *et al.* (2023) Fracture prevalence and its association with bone density among children living with HIV in Zimbabwe. *Aids* **37**, 759-767.
